# Design, synthesis, and biological evaluation of benzoheterocyclic sulfoxide derivatives as quorum sensing inhibitors in *Pseudomonas aeruginosa*

**DOI:** 10.1080/14756366.2023.2175820

**Published:** 2023-02-07

**Authors:** Shen Mao, Qiaoqiang Li, Zhikun Yang, Yasheng Li, Xinyi Ye, Hong Wang

**Affiliations:** a College of Pharmaceutical Science & Collaborative Innovation Center of Yangtze River Delta Region Green Pharmaceuticals, Zhejiang University of Technology, Hangzhou, P. R. China; b Department of Infectious Diseases, The First Affiliated Hospital of Anhui Medical University & Anhui Center for Surveillance of Bacterial Resistance, Hefei, P. R. China

**Keywords:** Sulfoxide, *Pseudomonas aeruginosa*, biofilm, quorum sensing, inhibitors

## Abstract

Six series of benzoheterocyclic sulfoxide derivatives were designed and synthesised as *Pseudomonas aeruginosa* (*P. aeruginosa*) quorum sensing inhibitors in this paper. We experimentally demonstrated that **6b** significantly inhibited the formation of *P. aeruginosa* PAO1 biofilm without affecting the growth. Further mechanistic studies showed that **6b** affected the luminescence of quorum sensing reported strain PAO1-*lasB*-*gfp* and the production of *P. aeruginosa* PAO1 elastase virulence factor which was regulated by *las* system. These experimental results indicate that **6b** acts as a quorum sensing inhibitor mainly through the *las* system. Furthermore, silico molecular docking studies demonstrated that **6b** and the *P. aeruginosa* quorum sensing receptor LasR were molecularly bound via hydrogen bonding interactions. Preliminary structure-activity relationship and docking studies illustrated that **6b** shows great promise as anti-biofilm compounds for further studies in order to solve the problem of microbial resistance in future.

## Introduction

With the excessive and indiscriminate abuse of antibiotics, the emergence of multiple drug resistant (MDR) bacterial strains has been bred. The abuse of antibiotics directly results in the death of 16 million human beings annually due to infections.^1–2^ It is necessary to emphasise that approximately 65% of infectious diseases are related to the proliferation of bacterial communities by forming biofilm.^3^ The bacteria in biofilms show more significant resistance to antibiotics and to the host immune responses than their plankton counterparts.^4^ In modern clinical microbiology, formation of biofilm is generally considered a pathogenicity characteristic of chronic infection.^5–6^ Biofilm formation is regulated by a phenomenon commonly known as quorum sensing (QS), in which bacteria release small signalling molecules, produce virulence factors, and form biofilms in a cell density-based manner.^2^^,^^7^^,^^8^ Therefore, inhibiting the formation of bacterial biofilms by interfering with bacterial quorum sensing is an efficient strategy to develop alternative therapies for the control and prevention of bacterial infections.

**Figure 1.**
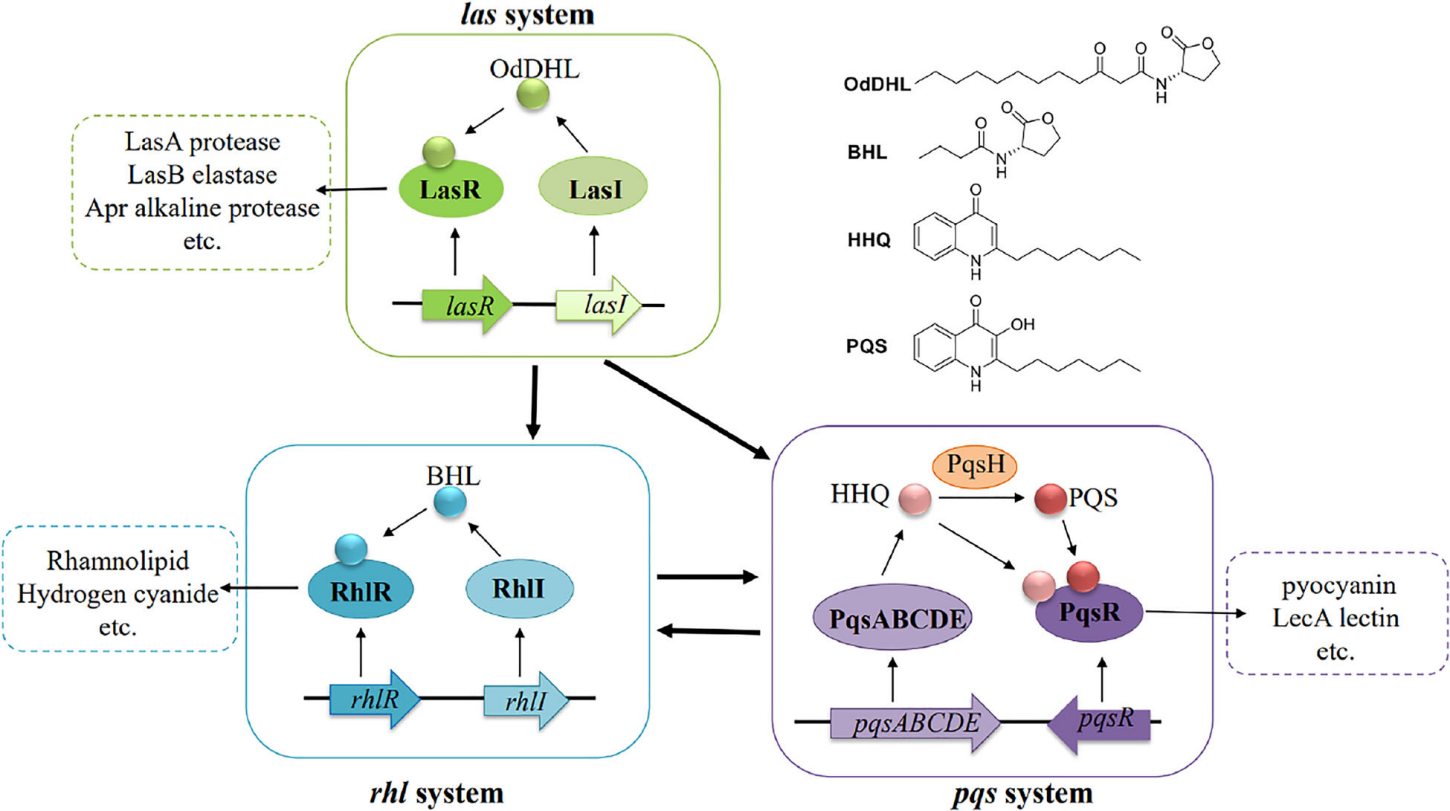
The main three QS signalling networks in *P. aeruginosa.*

Thus far, the QS network of *P. aeruginosa* is one of the most classical networks of quorum sensing and has been widely used in clinical studies.^9^ The QS network is mainly composed of three subsystems, *las*, *rhl* and *pqs*, which are interrelated and mutually regulated, leading to bacterial infection in the host (Figure 1).^10^ The *las* and *rhl* systems rely on two different *N*-acyl-L-homoserine lactone (AHL) type signal molecules, *N*-3-oxo-dodecanoyl homoserine lactone (3-oxo-C12-HSL, OdDHL) and *N*-butanoyl homoserine lactone (C4-HSL, BHL). The third system, *pqs*, employs 2-alkyl-4-quinolones (3, 2-hepyl-4-hydroxyquinoline (HHQ) or 2-heptyl-3- hydroxy-4-quinolone (PQS)) as the quorum sensing signal molecule (QSSMs).^11–13^ QS signals control about 6% of the *P. aeruginosa* genome through its complex but finely tuned mechanism.^14^

By analysing the quorum sensing signal system of *P. aeruginosa*, the receptor proteins LasR, RhlR, and PqsR, that specifically bind to signal molecules have been identified, which are closely related to the pathogenicity of *P. aeruginosa*.^15–18^ Among these, the LasR transcription activator protein controls the production and expression of numerous *P. aeruginosa* exoproducts, which is a hot topic in this field.^19^ Reports show that after the quorum sensing system of *P. aeruginosa* is blocked, the secretion of virulence factors and biofilm formation ability of *P. aeruginosa* are significantly reduced.^10^^,^^20^^,^^21^ Therefore, quorum sensing inhibitors (QSIs) are expected to become a novel drug against *P. aeruginosa* infections.

**Figure 2.**
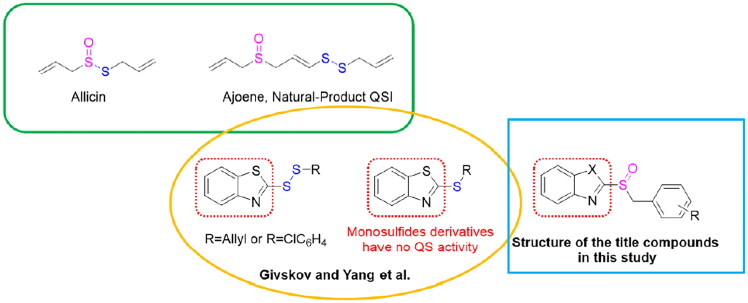
The design of the title compounds.

Studies have revealed that crude garlic extract had significant inhibitory effect on *P. aeruginosa* QS. The sulfur-containing compound ajoene was identified as QSIs in garlic extract by further bioassays.^22^ Givskov and Yang et al.^23^ first reported the synthesis of compounds with disulphide bond framework as QSIs in addition to the natural ajoene, which showed excellent quorum-sensing activity against *P. aeruginosa* and could reduce the production of QS-regulated virulence factors. They confirmed that disulphide derivatives with benzothiazole skeletons were pivotal functional groups for quorum sensing activity, but monosubstituted thioethers with benzothiazole skeletons did not show QS activity. It is well known that sulfoxides have a wide range of biological activities, as well as benzoheterocyclic skeletons,^24–25^ and both allicin and ajoene have sulfoxide structures. We hypothesised that benzoheterocyclic monosulfide may have quorum sensing activity after being oxidised to sulfoxides. We attempted to replace the disulphide bond by sulfoxide in hope of finding new lead compound to inhibit the quorum sensing of *P. aeruginosa* (Figure 2). Hence, we designed and synthesised a series of benzoheterocyclic sulfoxide compounds and evaluated the quorum sensing bioactivity against *P. aeruginosa*.

In this study, we report a series of benzoheterocyclic sulfoxide derivatives against biofilm inhibitory activity of *P. aeruginosa.* (A part of structures have been reported without bioactive results^26^). According to the expression of green fluorescent protein (GFP) reporter strains (PAO1-*lasB*-*gfp*, PAO1-*rhlA*-*gfp*, PAO1-*pqsA*-*gfp*), **6b** showed excellent biofilm inhibitory activities and significantly inhibited the expression of PAO1-*lasB*-*gfp* strains. Furthermore, we analysed the experimental results of *P. aeruginosa* PAO1 virulence factors including elastase, pyocyanin and rhamnolipid, as well as the silico molecular docking results with LasR receptor protein in quorum sensing. In summary, it was described that **6b** could reduce biofilm formation and virulence factor production by hydrogen bond interaction antagonistically binding to *P. aeruginosa* QS receptor protein.

## Results and discussion

### Chemistry

**Scheme 1.**
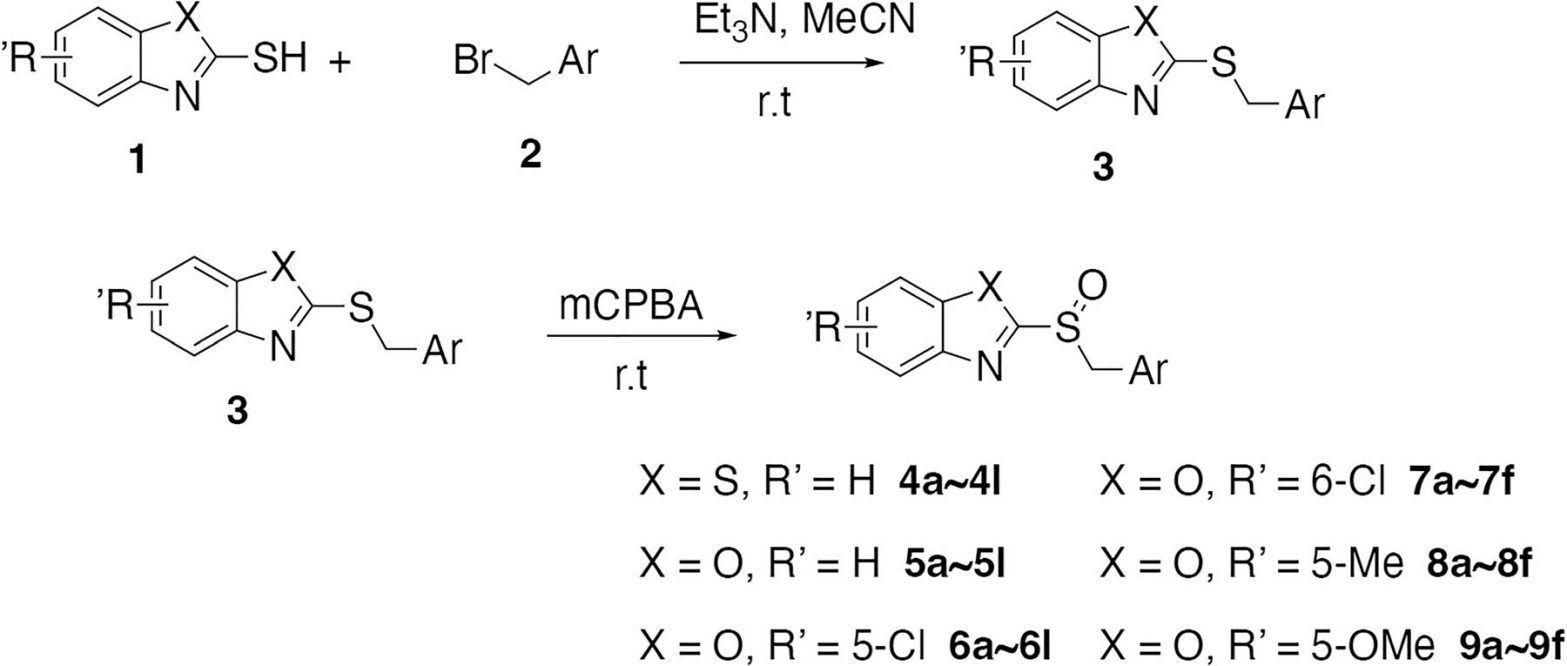
The synthetic route of the title compounds.

To verify the above hypothesis, we referred to 2-mercaptobenzothiazole disulphide with the excellent *P. aeruginosa* quorum-sensing activity to design the new candidate compounds.^23^ We retained the core part of the benzothiazole and replaced the disulphide bond with sulfoxide to synthesise a series of benzoheterocyclic sulfoxide derivatives. The synthetic routes of titled compounds were shown in Scheme 1. The key intermediates **3** (the different heterocyclic thioethers) were synthesised from benzyl bromides **1** and heterocyclic thiols **2** in the presence of triethylamine in acetonitrile. Benzyl bromide **1** was added dropwise into the mixture at room temperature.^26^ Then intermediates **3** were oxidised by *meta*-chloroperoxybenzoic acid in dichloromethane to obtain the different types of compounds. We first synthesised **4a-4l** (containing benzo thiazole), **5a-5l** (containing benzoxazole), the preliminary screening of *P. aeruginosa* biofilm activity showed that compounds with benzoxazole have good inhibitory effects. Furthermore, we considered chloro-substitution as an important halogen group for QS activity, a series of **6a-6l** (containing 5-chlorobenzoxazole) and **7a-7f** (containing 6-chlorobenzooxazole) were synthesised. To investigate whether the chloro-substitution on the benzoxazole ring was the key active site, we synthesised the series **8a-8f** (containing 5-methylbenzoxazole) and **9a-9f** (containing 5-methoxybenzoxazole).

### Biological evaluation

#### Evaluation of inhibition of P. aeruginosa biofilm and SAR studies

We tested the inhibitory activity of **4**, **5**, and **6** against *P. aeruginosa* PAO1 biofilm, benzimidazole used as positive control.^27–28^ As shown in Table 1, the **4a-4l** series compounds showed little, or no biofilm inhibitory effect compared to other groups. We also tested 5-chloro-substituted benzothiazole derivatives during the experiments, however, they showed little biofilm inhibitory activity (insignificant and not shown in results). The anti-biofilm results showed that **5b** and **6b** were more active than **4b**, while **5d** and **6d** were more active than **4d**. Moreover, **5e** and **6e** were more active than **4e**. All of above indicates that compounds containing benzoheterocyclic oxazole had better inhibitory activities of *P. aeruginos* PAO1 biofilm than benzoheterocyclic thiazole, and the benzoxazole ring might be the active functional group that plays a key role in biofilm activity. When the heterocyclic rings were benzoxazole structure, **5c**, **5d, 6c**, and **6d** showed relatively weaker activity than **5b** and **6b**. It indicated that the Rʺ might lead to better inhibitory activity when it was substituted at *para*-position. By analysing compounds with various substituents on the Rʺ phenyl ring, including those with electron-withdrawing groups (**5e**, **5f**), or with electronic donating groups (**6a**, **6 g**), they showed only a slight impact on the anti-biofilm activity. Moreover, compound **6b** with chloro-substitution had the best anti-biofilm effect, with an inhibition rate of 46.13 ± 0.79%. The inhibition activity of biofilm was better than fluoro- (**6e**, 38.64 ± 0.32%) and bromo- (**6f**, 14.60 ± 1.23%) substitutions at the *para*-position. We also tested the biofilm inhibitory activity of all compounds against *Pseudomonas aeruginosa* at 100 μM, and the SAR was basically consistent with the concentration of 50 μM.

**Table 1.** Biofilm inhibition rates of derivatives against the *P. aeruginosa* PAO1.

Entry	Compound.	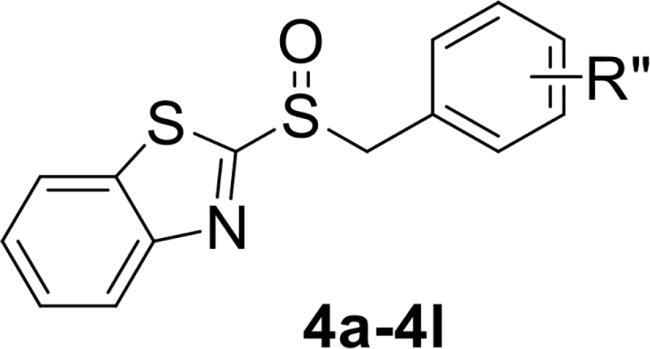	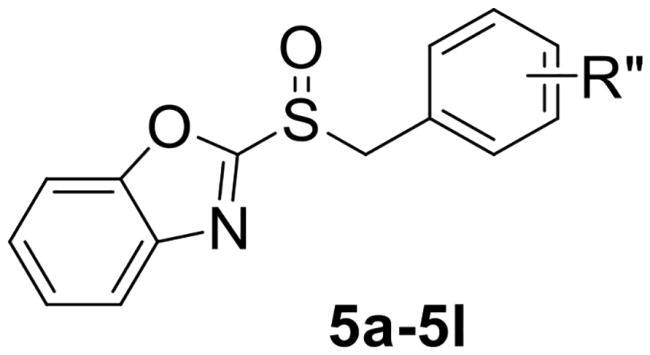	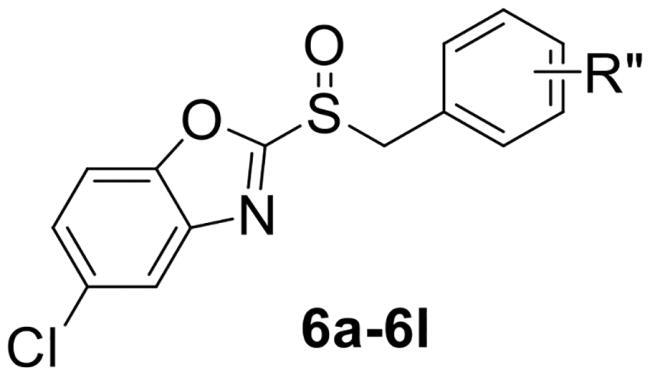
	R”	Inhibition rate^a.b^ (%)		
a	H	–4.75 ± 0.54	19.8 ± 0.70	12.84 ± 4.17
b	4-Cl	3.93 ± 1.07	39.18 ± 1.84	46.13 ± 0.72
c	3-Cl	16.64 ± 1.67	26.00 ± 0.69	13.77 ± 0.27
d	2-Cl	5.32 ± 2.00	24.25 ± 2.31	15.14 ± 2.25
e	4-F	–3.26 ± 0.76	15.94 ± 0.56	38.64 ± 0.32
f	4-Br	14.52 ± 3.50	26.06 ± 1.54	14.60 ± 1.23
g	4-Me	3.25 ± 0.55	31.39 ± 2.40	16.27 ± 1.68
h	4-napthyl	–8.59 ± 1.34	36.95 ± 0.57	9.95 ± 0.59
i	4-NO_2_	2.59 ± 0.05	7.74 ± 2.10	16.45 ± 1.69
j	4-CF_3_	–6.28 ± 1.07	26.24 ± 1.42	15.65 ± 0.84
k	3-MeO	6.15 ± 0.33	14.64 ± 3.12	15.85 ± 3.33
l	4-MeO	5.48 ± 0.32	20.99 ± 2.19	12.51 ± 1.41
Benzimidazole	16.78 ± 1.07			

^a^All data represent mean ± SD from different experiments performed in triplicate.

^b^The concentration of compounds were 50 μM, benzimidazole used as positive control. We also tested other concentrations, and the data was supplemented in supporting information.

Next, to further explore the effect of Rʺ as substitution on the anti-biofilm activity, compounds **7**, **8**, and **9** were synthesised. Antibiofilm activity was enhanced when chloro-substitution on aromatic ring involved. When 5- or 6-position of benzoxazole ring was substituted by chloro, **6b** and **7b** showed good inhibitory activities (Table 2). Especially, the inhibition rate of **7b** was 43.64 ± 2.49%, which meant that there was no difference between the chloro-substitutions at the 5- and 6-position on benzoxazole ring. However, when we changed the chloro-substitution on 5-position of benzoxazole ring to methyl or methoxy group, the inhibition rate of **8b** and **9b** were 19.93 ± 0.57% and 29.44 ± 0.39%. **8** and **9** with various substitutions had no particularly excellent inhibition effects except for **9b** and **9f**. A comparison with the serials data of **6** indicated that both the benzoxazole ring and the chloro-substitution were the key active functional group. Preliminary structure-activity relationships indicated that the benzoxazole heterocyclic ring was critical for optimal activity. In addition, the *para*-position on aromatic ring of benzyl group was an excellent choice for inhibitors design.

**Table 2.** Biofilm inhibition rates of derivatives against the *P. aeruginosa* PAO1.

Entry	Compound.	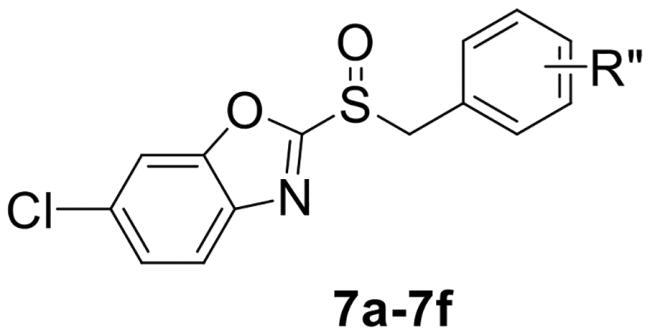	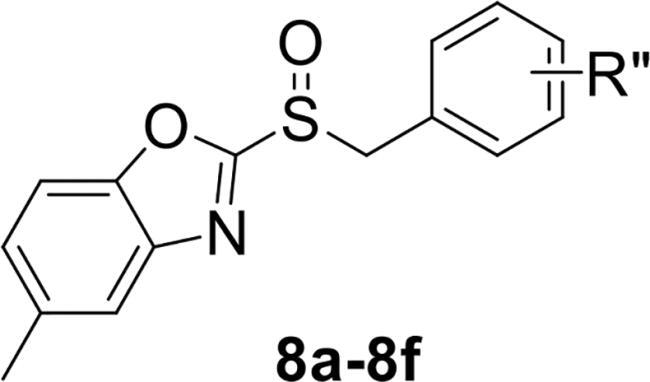	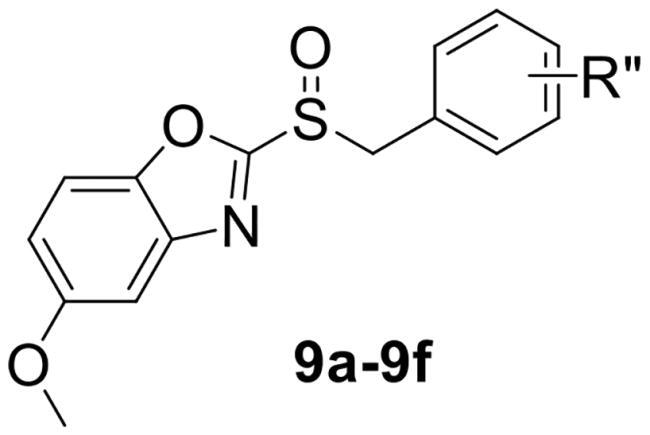
	R”	Inhibition rate^a.b^ (%)		
a	H	19.11 ± 2.12	11.92 ± 0.58	13.44 ± 0.37
b	4-Cl	43.64 ± 2.49	19.93 ± 0.57	29.44 ± 0.39
c	4-Me	23.75 ± 0.52	9.52 ± 1.7	13.33 + 0.96
d	4- NO_2_	22.30 ± 3.69	12.38 ± 2.54	13.18 + 0.69
e	4-CF_3_	26.45 ± 1.21	22.07 ± 0.82	18.75 ± 0.52
f	4-F	19.09 ± 1.16	14.19 ± 1.83	28.56 ± 0.45
Benzimidazole	16.78 ± 1.07			

^a^All data represent mean ± SD from different experiments performed in triplicate.

^b^The concentrations of compounds were 50 μM, benzimidazole used as positive control. We also tested other concentrations, the data supplemented in supporting information.

Previous reports have confirmed that disulphide bonds are bioactive, while, without the disulphide bonds in garlic analogues will render the QSI ineffective.^21^ The above experimental results verified our speculation that although the monosubstituted thioethers of the benzothiazole skeleton have no QS activity, they can inhibit the formation of *P. aeruginosa* PAO1 biofilm after oxidation to sulfoxide. Therefore, the sulfoxide bond is also an important framework for inhibitory biofilm and the sulfoxide derivatives also show quorum sensing activity, which has never been reported before. The structure-activity relationships indicated that the benzoxazole heterocycle and the sulfoxide bonds were the keys to the optimal activity, and the *para*-position of the benzene ring was the best choice for designing inhibitors.

#### Effect of 6b on biofilm growth and formation

**Figure 3.**
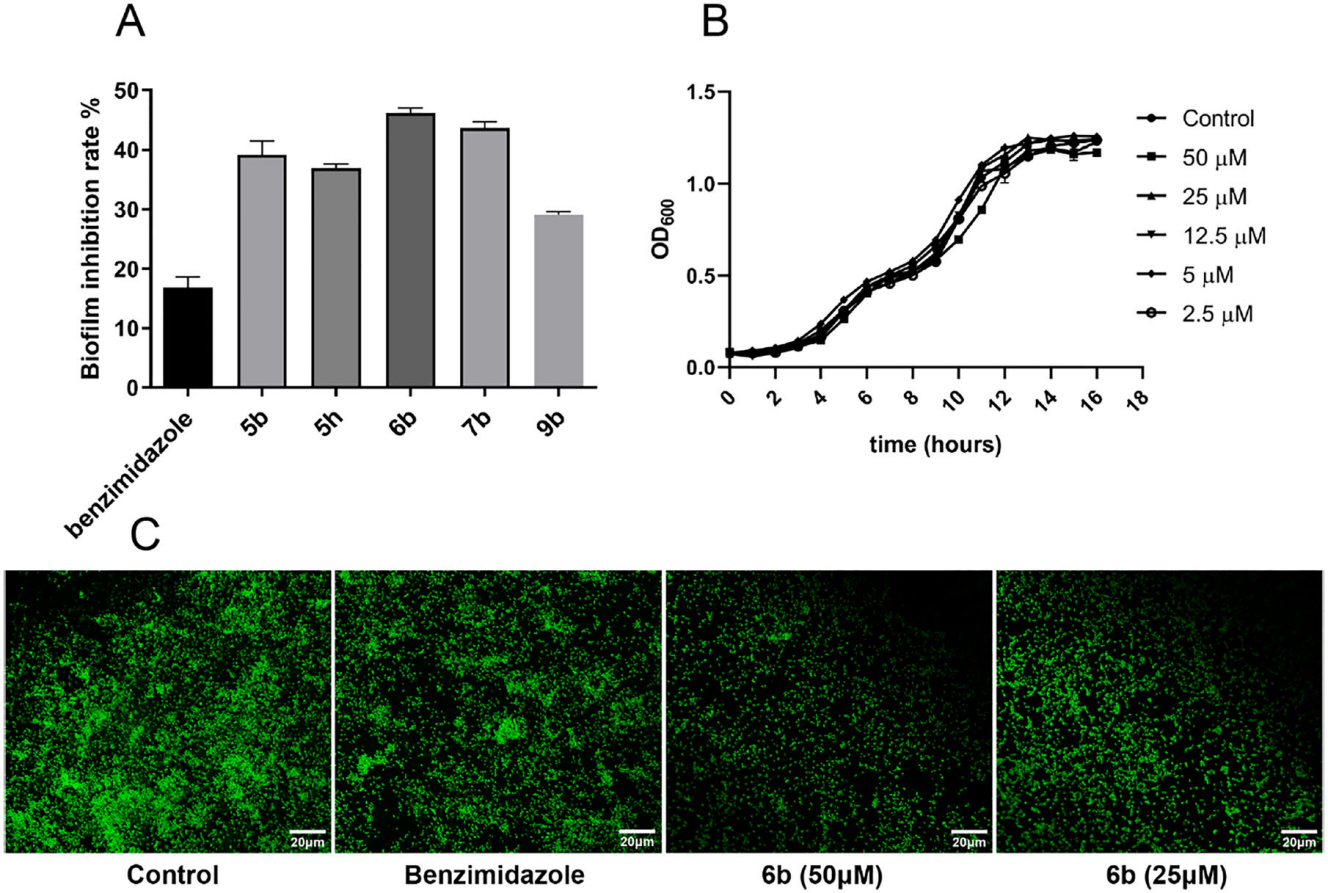
Effects of **6b** on *P. aeruginosa* PAO1 biofilm growth and formation. (A) Biofilm inhibition at 50 μM of **5b**, **5h**, **6d**, **7b**, **9b** for 24 h in microtiter plate. (B) Growth at different concentrations of **6b** (50, 25, 12.5, 5, 2.5 μM) for 16 h. (C) CLSM images of biofilm formed for 24 h with 50 μM and 25 μM of **6b**, and benzimidazole used as positive control (An equal amount of dimethyl sulfoxide solvent was set as control group). Data represents the average of three-independent determinations of triplicate samples.

Based on the above results, compounds **5b**, **5h**, **6b**, **7b** and **9b** with better biofilm inhibitory activities were selected for further study (Figure 3A). All compounds were cultured for 24 h and the OD value at 600 nm was evaluated before biofilm experiment. The normal growth of *P. aeruginosa* PAO1 was unaffected at concentration of 50 μM. **6b** was chosen as an example and tested at concentrations of 50 μM, 25 μM, 12.5 μM, 5 μM and 2.5 μM. An equal amount of dimethyl sulfoxide (DMSO) was added to the control group. The effect of **6b** on *P. aeruginosa* PAO1 growth was assessed hourly by monitoring OD_600_ culture (Figure 3B). As a result, **6b** had no effect on the growth of *P. aeruginosa* PAO1. Cytotoxicity of **6b** was also evaluated (see in Supporting information Figure S-1). In addition, it was observed that **6b** impeded biofilm formation in the images taken by confocal laser scanning microscopy (CLSM) (Figure 3C). The density of the biofilm formed by **6b** at 50 μM was lower than that of the control group, and the biofilm formation was also inhibited at 25 μM.

#### Effect of 6b on QS system report strains

According to the regulation process of the QS system of *P. aeruginosa*, Givskov et al.^21^^,^^29–31^ fused their respective promoters in the *las*, *rhl* and *pqs* pathways with unstable green fluorescent protein (GFP) to construct three reporter strains PAO1-*lasB*-*gfp*, PAO1-*rhlA*-*gfp* and PAO1-*pqsA*-*gfp*, and a screening system for detecting small molecule QSI based on the growth of bacteria was established. *LasB* encodes the virulence factor elastase, which has been shown to be transcriptionally controlled by LasR.^32^ In *rhl* system, *rhlA* is the first gene of *rhlA* and *rhlB* operons, encoding rhamnotransferase required for the synthesis of rhamnolipid; while *pqsA* is the operation required by the first gene of *pqsABCDE* to produce *pqs* signal, and the *pqs* system can enhance the pyocyanin expression of *P. aeruginosa*.^33–34^

**Figure 4.**
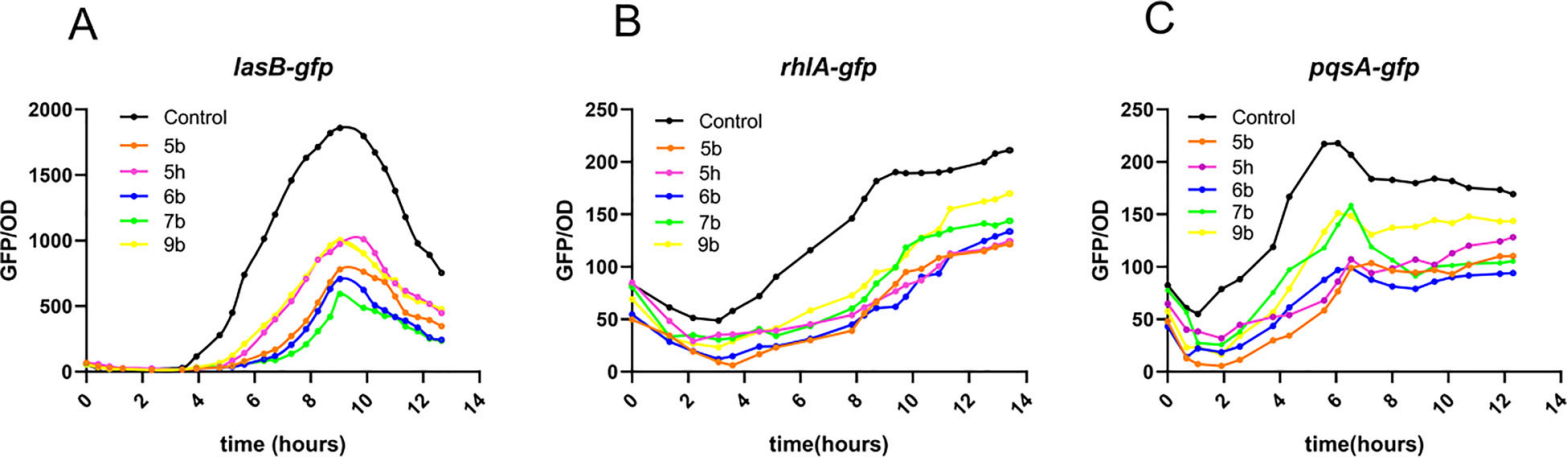
The effects of **5b**, **5h**, **6d**, **7b**, **9b** on QS system report strain (A) PAO1-*lasB*-*gfp*, (B) PAO1-*rhlA*-*gfp*, and (C) PAO1-*pqsA*-*gfp*. The experiments were done triplicate.

**Figure 5.**
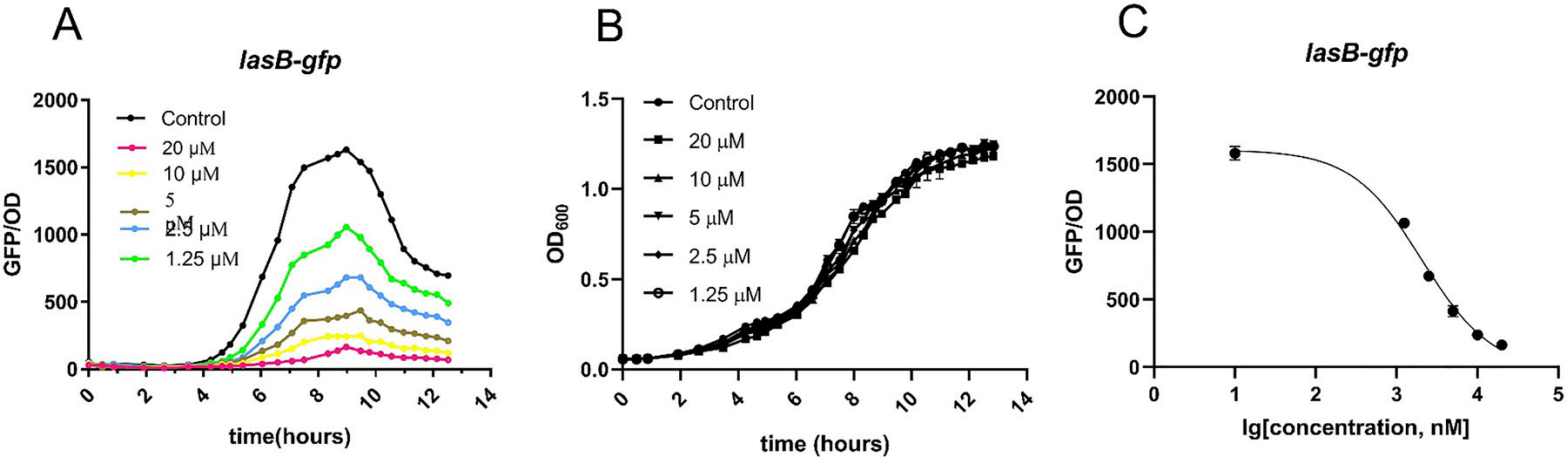
(A) Dose-dependent inhibition curves of **6b** incubated with the QS monitors PAO1-*lasB*-*gfp*. (B) Growth at different concentrations of **6b** (20, 10, 5, 2.5, 1.25 μM). (C) IC_50_ values calculations were based on three biological replicates and performed by nonlinear fitting, using Graphpad Prism 6 software. The IC_50_ values of **6b** was 2.08 ± 0.25 μM for PAO1-*lasB*-*gfp*. The experiments were also done in triplicate.

**Figure 6.**
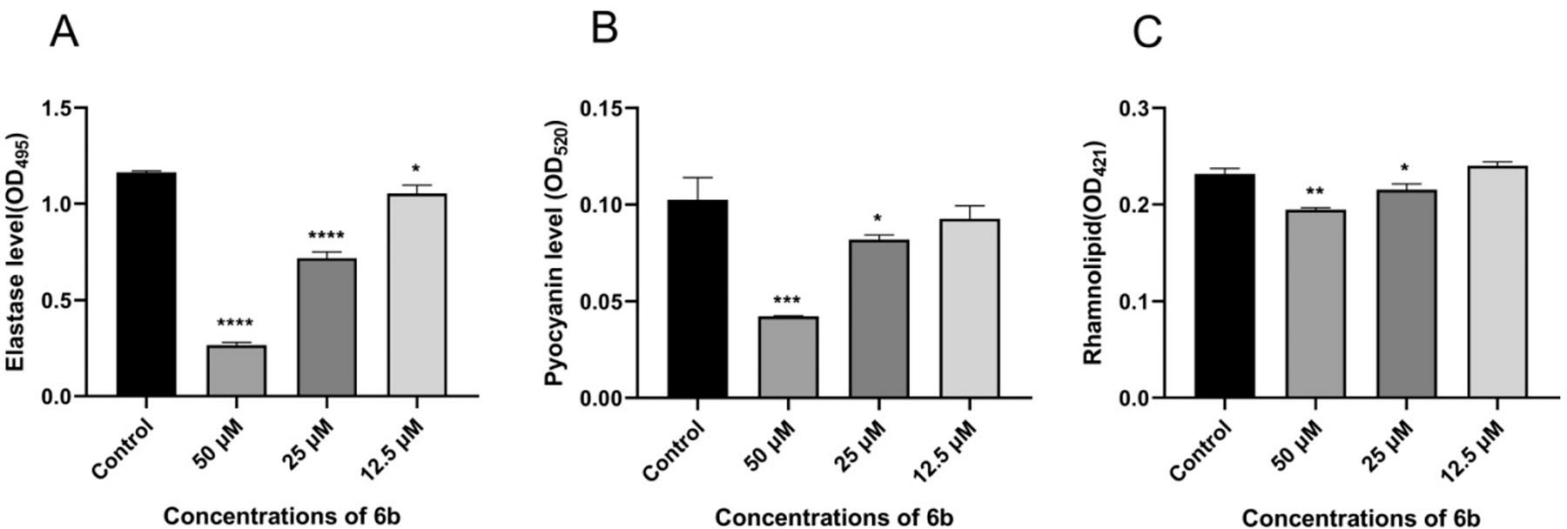
Effects of **6b** on the production of (A) elastase (B) pyocyanin (C) rhamnolipid at different concentrations (50, 25, 12.5 and 0 μM). Error bars are means ± SDs. * = *p* < 0.05 versus the control, ** = *p* < 0.01 versus the control, *** = *p* < 0.001 versus the control.

**Figure 7.**
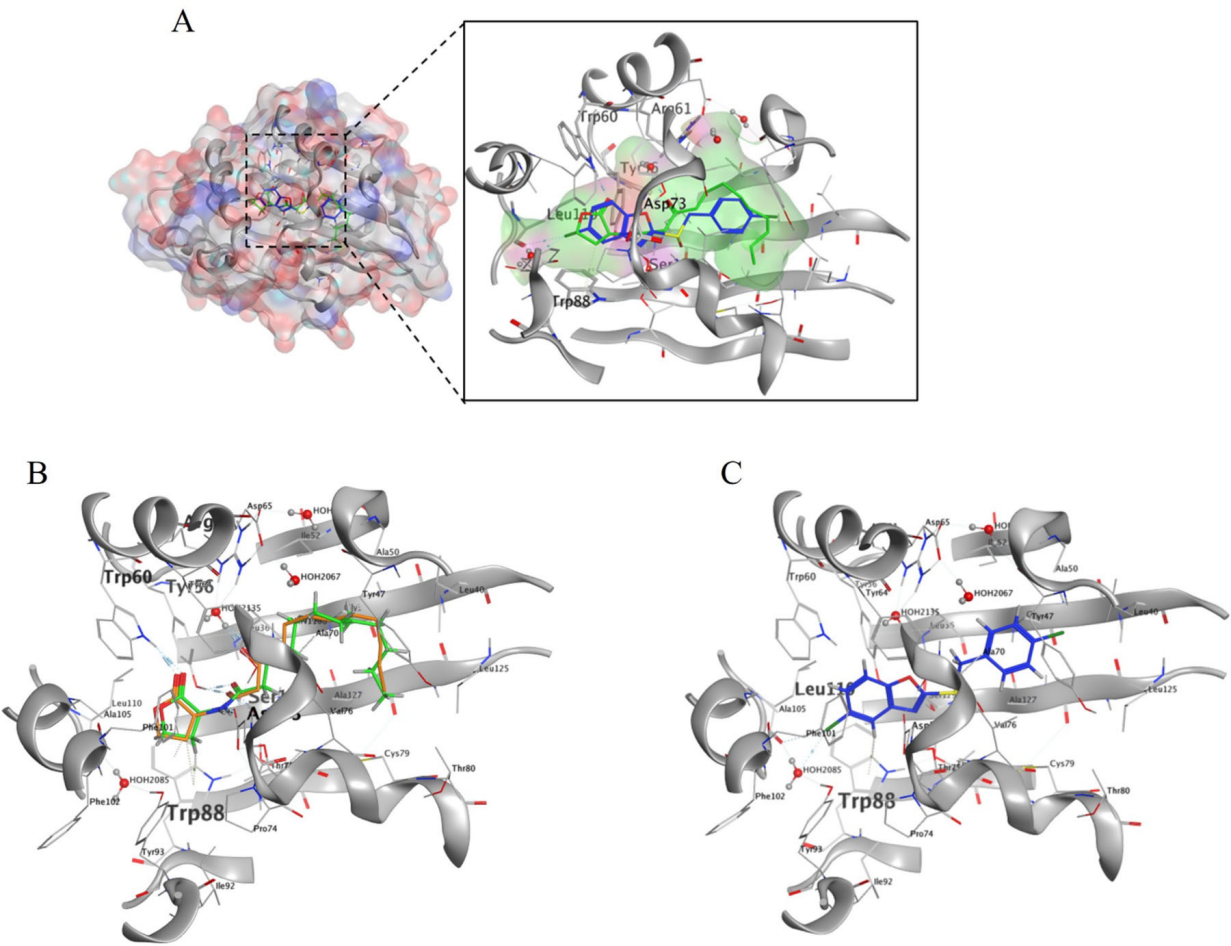
Predicted binding model of compounds (OdDHL, **6b**) and LasR (PDB code: 2uv0). (A) The pocket surface view of interactions between OdDHL, **6b** with receptor protein lasR; (B) Superposition of native docking of OdDHL and OdDHL in LasR X-ray crystal structure, showing as OdDHL in green, OdDHL in LasR X-ray crystal structure in orange; (C) Details of **6b** binding. **6b** was shown in blue. The Residues involved in interactions with compounds are depicted as sticks in black and named in red. The hydrogen bonds are shown as aqua dashed lines.

To further verify the QS activity of **5b**, **5h**, **6b**, **7b** and **9b**, the PAO1-*lasB*-*gfp*, PAO1-*rhlA*-*gfp* and PAO1-*pqsA*-*gfp* strains were introduced for screening. As shown in Figure 4, **6b** and **7b** could significantly inhibit the fluorescence expression of PAO1-*lasB*-*gfp* strain at 20 μM. **5b**, **5h**, **6b**, **7b**, **9b** also had a little inhibitory effect on PAO1-*rhlA*-*gfp* and PAO1-*pqsA*-*gfp* strains, but the effect was not as significant as PAO1-*lasB*-*gfp*. Furthermore, **6b** was selected as the key research object. Under the premise of function-permitting growth of the reporter strain (Figure 5B), **6b** showed a significant dose-dependence on PAO1-*lasB*-*gfp* strain at different concentrations of 20 μM, 10 μM, 5 μM, 2.5 μM and 1.25 μM (Figure 5A). From the obtained dose-response curve, the IC_50_ value was estimated to be 2.08 ± 0.25 μM (Figure 5C).

#### Effect of 6b on virulence factors

As it is mentioned above, the QS system of *P. aeruginosa* controls the production of various virulence factors and biofilm formation. To further verify the active mechanism of **6b** inhibiting biofilm to *las* system, we measured the effects of **6b** on the production of three virulence factors, elastase, pyocyanin, and rhamnolipid. The results were showed that **6b** significantly reduced the production of elastase at 50 μM, 25 μM and 12.5 μM in a concentration-dependent manner (Figure 6A). **6b** only inhibited the production of pyocyanin at high concentrations, while **6b** has almost no effect on the production of rhamnolipid. *Las*, *rhl* and *pqs* systems control elastase, rhamnolipid and pyocyanin respectively. These results suggested that the presence of **6b** inhibited the QS *lasB* gene and reduced the production of the virulence factor elastase. It was further demonstrated that **6b** inhibited biofilm information through the *las* pathway.

#### Molecular docking study

The *las* QS system uses OdDHL as autoinducer, which is synthesised by the lasI protein. Upon binding to the autoinducer OdDHL, the receptor protein LasR gets activated and forms a complex. The LasR-OdDHL complex bind to conserve *las-rhl* cassettes located in target genes promoters, thereby activating their transcriptional expression.^10^ In order to further explore the molecular mechanism of **6b** regulating QS system, silico molecular docking was performed to predict the binding model of **6b** with its homologous signal receptor protein LasR. The lowest binding energy for the docked conformations was chosen from 30 docking conformations as modes for the corresponding compound. As shown in Figure 7, **6b** was well positioned by the active pocket of LasR, the diaromatic character occupying several cavities in the LasR protein. Specifically, chlorine and benzene part of benzoxazole **6b** formed hydrogen bond and π-H interactions with the residues Leu 110 and Trp 88, while oxygen and nitrogen in OdDHL were observed to form hydrogen bond interactions with Trp 60, Trp 88, Asp 73, Tyr 56 and Ser 129, respectively. It indicated the importance of substituted benzoxazole with chlorine for *P. aeruginosa* biofilm activity. Besides, the benzene substituted with chlorine in **6b** occupied the same cavities in the LasR protein as the long alkyl chain in OdDHL. Docking models revealed that **6b** interacted with the amino acid residues of LasR, which explained the observed SARs.

## Conclusion

To solve the drug resistance problem of *P. aeruginosa* mediated by biofilm, medicinal chemists have proposed an efficient strategy that could reduce the production of pathogen extracellular virulence factors without killing the pathogen, which can also alleviate the drug resistance problem a certain extent. According to the excellent QSIs performance of sulfur-containing compounds, we designed and synthesised a series of benzoheterocyclic sulfoxides and evaluated their ability to inhibit QS *in vitro*. The results showed that **6b** significantly inhibited the biofilm formation of *P. aeruginosa*, with an inhibition rate of 46.13 ± 0.72%. The results of mechanism study confirmed that **6b** could effectively inhibit *las* system in a dose-dependent manner, and the IC_50_ value of inhibitory concentration against PAO1-*lasB*-*gfp* strain was 2.08 ± 0.25 μM. In addition, **6b** attenuated the production of elastase, a virulence factor of *P. aeruginosa* PAO1. Molecular docking analysis showed that **6b** and LasR receptor protein inhibited the expression of *las* system gene by forming hydrogen bonds, thus, inhibiting the production of virulence factors and *P. aeruginosa* biofilm production. Our study provides a new approach for designing quorum sensing inhibitors as new antimicrobial resistance agents. Sulfoxide derivatives have been proposed as a new QSIs model, which will provide a reference for the medical community to solve the global drug resistance problem. In future studies, we will optimise more efficient and practical QSIs based on **6b** to develop new anti-infection drugs.

## Experimental section

### Chemistry

#### Materials and methods

The solvents and reagents used in this experiment were obtained commercially without further purification. All compounds were structurally identified by 1H NMR and 13 C NMR and high-resolution mass spectrometry (ESI-HRMS). Unless otherwise stated, ^1^H and ^13^C NMR spectra were recorded on Bruker Avance III 400 at 600 and 150 MHz or 400 and 100 MHz spectrometer. Chemical shifts are recorded as *δ* in units of parts per million (ppm), while tetramethylsilane (TMS) was used as an internal standard. Compounds were dissolved in CDCl_3_. High resolution mass spectra were obtained on an SCIEX series X500B QTOF mass spectrometer. Thin-layer chromatography (TLC) was performed using Huanghai GF254 Silica gel plates. Column chromatography was performed using silica gel (200–300 mesh, Beijing, China) with a linear solvent gradient.

#### General synthesis of compounds 4a–9f

Benzyl bromide **1** (1.2 mmol) was added dropwise to a solution of heterocyclic thiol **2** (1.0 mmol) and Et_3_N (1.5 mmol) in MeCN (10 mL) at room temperature. The reaction solution was quenched with 6 M HCl aqueous solution, and then extracted with EtOAc (3 × 20 mL). The combined organic phase was dried with anhydrous Na_2_SO_4_, concentrated in vacuum and purified by column chromatography (PE/EA = 50/1 ∼ 30/1 ratio) to obtain intermediates thioether **3**. Then intermediates **3** (1.0 mmol) and *meta*-chloroperoxybenzoic acid (1.0 mmol) are added in dichloromethane to react and extracted with saturated sodium thiosulphate solution, dried and purified by column chromatography to obtain the different title compounds **4a-4l**, **5a-5l**, **6a-6l**, **7a-7f**, **8a-8f**, **9a-9f**.

2-(benzylsulfinyl) benzo[*d*]thiazole (**4a**) white solid, yield 85%. ^1^H NMR (400 MHz, Chloroform-*d*) δ 8.13 − 8.09 (m, 1H), 7.94 (dd, *J* = 8.0, 1.1 Hz, 1H), 7.61 − 7.55 (m, 1H), 7.49 (ddd, *J* = 8.4, 7.2, 1.1 Hz, 1H), 7.29 (ddd, *J* = 9.5, 6.2, 4.3 Hz, 3H), 7.19 − 7.15 (m, 2H), 4.59 − 4.28 (m, 2H). ^13^C NMR (101 MHz, CDCl_3_) δ 176.86, 153.69, 135.99, 130.45, 128.74, 128.69, 128.30, 126.92, 126.15, 123.89, 122.24, 62.77. ESI-HRMS: calcd for C_14_H_11_NOS_2_ [M + H]^+^, 274.0360; found, 274.0361.

2-((4-chlorobenzyl) sulfinyl) benzo[*d*]thiazole (**4b**) white solid, yield 81%. ^1^H NMR (400 MHz, Chloroform-*d*) δ 8.13 − 8.08 (m, 1H), 7.97 − 7.94 (m, 1H), 7.62 − 7.55 (m, 1H), 7.53 − 7.47 (m, 1H), 7.23 (d, *J* = 8.4 Hz, 2H), 7.11 − 7.04 (m, 2H), 4.62 − 4.11 (m, 2H). ^13^C NMR (101 MHz, CDCl_3_) δ 176.30, 153.57, 135.86, 134.88, 131.61, 128.75, 126.91, 126.52, 126.15, 123.79, 122.20, 61.47. ESI-HRMS: calcd for C_14_H_10_ClNOS_2_ [M + H]^+^, 307.9971; found, 307.9969.

2-((3-chlorobenzyl) sulfinyl) benzo[*d*]thiazole (**4c**) white solid, yield 89%. ^1^H NMR (600 MHz, Chloroform-*d*) δ 8.11 (dt, *J* = 8.3, 0.9 Hz, 1H), 7.96 (ddd, *J* = 8.1, 1.2, 0.6 Hz, 1H), 7.59 (ddd, *J* = 8.3, 7.2, 1.2 Hz, 1H), 7.50 (ddd, *J* = 8.3, 7.2, 1.2 Hz, 1H), 7.28 (ddd, *J* = 8.1, 2.1, 1.1 Hz, 1H), 7.22 − 7.15 (m, 2H), 7.06 (dt, *J* = 7.6, 1.3 Hz, 1H), 4.39 (dd, *J* = 107.1, 13.2 Hz, 2H). ^13^C NMR (151 MHz, CDCl_3_) δ 176.48, 153.69, 135.95, 134.54, 130.45, 130.29, 129.84, 128.91, 128.56, 127.02, 126.27, 123.95, 122.26, 62.10. ESI-HRMS: calcd for C_14_H_10_ClNOS_2_ [M + H]^+^, 307.9971; found, 307.9973.

2-((2-chlorobenzyl) sulfinyl) benzo[*d*]thiazole (**4d**) white solid, yield 90%. ^1^H NMR (600 MHz, Chloroform-*d*) δ 8.23 − 7.90 (m, 2H), 7.58 (ddd, *J* = 8.3, 7.2, 1.2 Hz, 1H), 7.50 (ddd, *J* = 8.2, 7.2, 1.2 Hz, 1H), 7.41 (dd, *J* = 8.0, 1.2 Hz, 1H), 7.32 − 7.26 (m, 2H), 7.22 (td, *J* = 7.4, 1.3 Hz, 1H), 4.62 (dd, *J* = 175.6, 12.9 Hz, 2H). ^13^C NMR (151 MHz, CDCl_3_) δ 176.60, 153.70, 136.01, 135.15, 132.91, 130.35, 129.81, 127.07, 127.01, 126.97, 126.31, 124.13, 122.26, 61.11. ESI-HRMS: calcd for C_14_H_10_ClNOS_2_ [M + H]^+^, 307.9971; found, 307.9976.

2-((4-fluorobenzyl) sulfinyl) benzo[*d*]thiazole (**4e**) white solid, yield 86%. ^1^H NMR (600 MHz, Chloroform-*d*) δ 8.10 (dt, *J* = 8.3, 0.9 Hz, 1H), 7.94 (dt, *J* = 8.0, 0.9 Hz, 1H), 7.58 (ddd, *J* = 8.3, 7.2, 1.2 Hz, 1H), 7.49 (ddd, *J* = 8.3, 7.2, 1.2 Hz, 1H), 7.14 − 7.10 (m, 2H), 6.97 − 6.92 (m, 2H), 4.41 (dd, *J* = 101.5, 13.4 Hz, 2H). ^13^C NMR (151 MHz, CDCl_3_) δ 176.60, 163.92, 162.28, 153.71, 135.99, 132.22, 132.16, 127.00, 126.23, 123.91, 122.29, 115.79, 115.64, 61.57. ESI-HRMS: calcd for C_14_H_10_FNOS_2_ [M + H]^+^, 292.0266; found, 292.0272.

2-((4-bromobenzyl) sulfinyl) benzo[*d*]thiazole (**4f**) white solid, yield 85%. ^1^H NMR (600 MHz, Chloroform-*d*) δ 8.10 (dt, *J* = 8.3, 0.9 Hz, 1H), 7.95 (dt, *J* = 8.1, 0.9 Hz, 1H), 7.58 (ddd, *J* = 8.3, 7.2, 1.2 Hz, 1H), 7.49 (ddd, *J* = 8.2, 7.2, 1.1 Hz, 1H), 7.42 − 7.36 (m, 2H), 7.03 − 6.99 (m, 2H), 4.38 (dd, *J* = 101.8, 13.3 Hz, 2H). ^13^C NMR (151 MHz, CDCl_3_) δ 176.45, 153.69, 135.98, 131.99, 131.79, 127.18, 126.99, 126.23, 123.90, 123.21, 122.30, 61.67. ESI-HRMS: calcd for C_14_H_10_BrNOS_2_ [M + H]^+^, 351.9465; found, 351.9469.

2-((4-methylbenzyl) sulfinyl) benzo[*d*]thiazole (**4 g**) white solid, yield 84%. ^1^H NMR (400 MHz, Chloroform-*d*) δ 8.10 (d, *J* = 7.6 Hz, 1H), 7.96 (dd, *J* = 8.1, 1.1 Hz, 1H), 7.57 (dt, *J* = 8.2, 1.0 Hz, 1H), 7.49 (tt, *J* = 7.2, 0.9 Hz, 1H), 7.08 (d, *J* = 1.7 Hz, 4H), 4.58 − 4.20 (m, 2H), 2.31 (s, 3H). ^13^C NMR (101 MHz, CDCl_3_) δ 177.02, 153.71, 138.71, 135.99, 130.35, 129.45, 126.90, 126.12, 125.18, 123.89, 122.26, 62.66, 21.23. ESI-HRMS: calcd for C_15_H_13_NOS_2_ [M + H]^+^, 288.0517; found, 288.0522.

2-((naphthalen-2-ylmethyl) sulfinyl) benzo[*d*]thiazole (**4h**) white solid, yield 79%. ^1^H NMR (400 MHz, Chloroform-*d*) δ 8.11 (dd, *J* = 8.4, 1.0 Hz, 1H), 7.90 (dd, *J* = 8.1, 1.0 Hz, 1H), 7.81 − 7.68 (m, 4H), 7.58 (ddd, *J* = 8.4, 7.2, 1.2 Hz, 1H), 7.50 − 7.41 (m, 3H), 7.25 − 7.22 (m, 1H), 4.73 − 4.46 (m, 2H). ^13^C NMR (101 MHz, CDCl_3_) δ 176.90, 153.70, 135.98, 133.12, 130.25, 128.46, 127.97, 127.66, 127.44, 126.93, 126.54, 126.39, 126.15, 125.80, 123.88, 122.26, 63.24. ESI-HRMS: calcd for C_18_H_13_NOS_2_ [M + H]^+^, 324.0517; found, 324.0519.

2-((4-nitrobenzyl) sulfinyl) benzo[*d*]thiazole (**4i**) yellow solid, yield 84% ^1^H NMR (400 MHz, Chloroform-*d*) δ 8.09 (dd, *J* = 8.9, 2.3 Hz, 3H), 7.93 (dd, *J* = 8.1, 1.1 Hz, 1H), 7.63 − 7.58 (m, 1H), 7.53 − 7.48 (m, 1H), 7.31 − 7.28 (m, 2H), 4.68 − 4.43 (m, 2H). ^13^C NMR (101 MHz, CDCl_3_) δ 175.53, 153.61, 148.00, 135.88, 135.30, 131.37, 127.21, 126.49, 123.94, 123.56, 122.35, 60.92. ESI-HRMS: calcd for C_14_H_10_N_2_O_3_S_2_ [M + H]^+^, 319.0211; found, 319.0198.

2-((4-(trifluoromethyl) benzyl) sulfinyl) benzo[*d*]thiazole (**4j**) white solid, yield 78%. ^1^H NMR (400 MHz, Chloroform-*d*) δ 8.11 (d, *J* = 8.2 Hz, 1H), 7.95 (d, *J* = 8.1 Hz, 1H), 7.61 (dd, *J* = 7.3, 1.0 Hz, 1H), 7.55 − 7.47 (m, 4H), 7.28 (s, 1H), 4.58 (d, *J* = 13.2 Hz, 1H), 4.41 (d, *J* = 13.2 Hz, 1H). ^13^C NMR (101 MHz, CDCl_3_) δ 176.15, 153.68, 135.99, 132.24, 130.83, 127.11, 126.37, 125.57, 125.53, 123.95, 122.32, 61.70. ESI-HRMS: calcd for C_15_H_10_F_3_NOS_2_ [M + H]^+^, 342.0234; found, 342.0232.

2-((3-methoxybenzyl) sulfinyl) benzo[*d*]thiazole (**4k**) white solid, yield 75%. ^1^H NMR (400 MHz, Chloroform-*d*) δ 8.10 (dt, *J* = 8.3, 0.9 Hz, 1H), 7.95 (dt, *J* = 8.1, 0.9 Hz, 1H), 7.57 (ddd, *J* = 8.3, 7.2, 1.3 Hz, 1H), 7.48 (ddd, *J* = 8.3, 7.2, 1.2 Hz, 1H), 7.19 (dd, *J* = 8.3, 7.5 Hz, 1H), 6.85 (ddd, *J* = 8.4, 2.6, 0.9 Hz, 1H), 6.79 (dt, *J* = 7.5, 1.2 Hz, 1H), 6.66 (dd, *J* = 2.5, 1.6 Hz, 1H), 4.40 (dd, *J* = 71.2, 13.1 Hz, 2H), 3.60 (s, 3H). ^13^C NMR (101 MHz, CDCl_3_) δ 177.00, 159.69, 153.71, 136.04, 129.71, 126.96, 126.20, 123.89, 122.85, 122.29, 115.14, 115.11, 63.03, 55.08. ESI-HRMS: calcd for C_15_H_13_NO_2_S_2_ [M + H]^+^, 304.0466; found, 304.0463.

2-((4-methoxybenzyl) sulfinyl) benzo[*d*]thiazole (**4 l**) white solid, yield 78%. ^1^H NMR (400 MHz, Chloroform-*d*) δ 8.10 (dt, *J* = 8.3, 0.9 Hz, 1H), 7.95 (dt, *J* = 8.0, 0.9 Hz, 1H), 7.58 (ddd, *J* = 8.3, 7.2, 1.3 Hz, 1H), 7.48 (ddd, *J* = 8.3, 7.2, 1.2 Hz, 1H), 7.10 − 7.07 (m, 2H), 6.81 − 6.77 (m, 2H), 4.53 − 4.26 (m, 2H), 3.77 (s, 3H). ^13^C NMR (101 MHz, CDCl_3_) δ 177.04, 159.97, 153.69, 135.96, 131.65, 126.85, 126.05, 123.83, 122.22, 120.08, 114.11, 62.24, 55.21. ESI-HRMS: calcd for C_15_H_13_NO_2_S_2_ [M + Na]^+^, 326.0285; found, 326.0258.

2-(benzylsulfinyl) benzo[*d*]oxazole (**5a**) white solid, yield 74%. ^1^H NMR (600 MHz, Chloroform-*d*) δ 7.83 − 7.79 (m, 1H), 7.61 (dt, *J* = 8.2, 0.8 Hz, 1H), 7.49 − 7.42 (m, 2H), 7.34 − 7.27 (m, 3H), 7.24 (dt, *J* = 6.7, 1.6 Hz, 2H), 4.64 − 4.52 (m, 2H). ^13^C NMR (151 MHz, CDCl_3_) δ 163.73, 151.64, 140.40, 130.23, 129.02, 128.26, 127.09, 125.60, 123.98, 122.63, 121.26, 111.46, 110.14, 109.84, 60.80. ESI-HRMS: calcd for C_14_H_11_NO_2_S [M + H]^+^, 258.0589; found, 258.0590.

2-((4-chlorobenzyl) sulfinyl) benzo[*d*]oxazole (**5b**) yellow solid, yield 77%. ^1^H NMR (600 MHz, Chloroform-*d*) δ 7.85 − 7.79 (m, 1H), 7.61 (d, *J* = 7.6 Hz, 2H), 7.46 (ddd, *J* = 12.1, 7.7, 1.1 Hz, 2H), 7.27 (d, *J* = 3.1 Hz, 1H), 7.17 (d, *J* = 8.4 Hz, 2H), 4.65 − 4.48 (m, 2H). ^13^C NMR (151 MHz, CDCl_3_) δ 163.37, 151.66, 140.33, 135.31, 131.55, 129.21, 127.20, 126.71, 125.71, 121.27, 111.48, 110.12, 109.91, 59.76. ESI-HRMS: calcd for C_14_H_10_ClNO_2_S [M + H]^+^, 292.0199; found, 292.0199.

2-((3-chlorobenzyl) sulfinyl) benzo[*d*]oxazole (**5c**) white solid, yield 84%. ^1^H NMR (600 MHz, Chloroform-*d*) δ 7.87 − 7.84 (m, 1H), 7.64 (dd, *J* = 7.8, 1.2 Hz, 1H), 7.52 − 7.46 (m, 2H), 7.33 − 7.31 (m, 1H), 7.26 − 7.23 (m, 1H), 7.18 − 7.14 (m, 2H), 4.63 − 4.52 (m, 2H). ^13^C NMR (151 MHz, CDCl_3_) δ 134.86, 130.30, 130.20, 129.25, 128.43, 127.24, 125.73, 124.06, 122.68, 121.30, 111.49, 110.16, 109.93, 60.05. ESI-HRMS: calcd for C_14_H_10_ClNO_2_S [M + H]^+^, 292.0199; found, 292.0199.

2-((2-chlorobenzyl) sulfinyl) benzo[*d*]oxazole (**5d**) white solid, yield 80%. ^1^H NMR (600 MHz, Chloroform-*d*) δ 7.83 (dt, *J* = 7.9, 0.9 Hz, 1H), 7.66 − 7.63 (m, 1H), 7.50 (td, *J* = 7.8, 1.3 Hz, 1H), 7.47 − 7.41 (m, 2H), 7.30 (ddd, *J* = 7.1, 4.3, 2.5 Hz, 2H), 7.21 (td, *J* = 7.5, 1.2 Hz, 1H), 4.80 (dd, *J* = 119.7, 12.8 Hz, 2H). ^13^C NMR (151 MHz, CDCl_3_) δ 163.83, 151.69, 140.39, 134.99, 132.84, 130.60, 129.95, 127.35, 127.26, 126.66, 125.65, 121.40, 111.52, 58.77. ESI-HRMS: calcd for C_14_H_10_ClNO_2_S [M + H]^+^, 292.0199; found, 292.0199.

2-((4-fluorobenzyl) sulfinyl) benzo[*d*]oxazole (**5e**) yellow solid, yield 68%. ^1^H NMR (600 MHz, Chloroform-*d*) δ 7.82 (d, *J* = 7.4 Hz, 1H), 7.62 − 7.58 (m, 1H), 7.49 − 7.42 (m, 2H), 7.21 (dd, *J* = 8.6, 5.3 Hz, 2H), 6.97 (t, *J* = 8.6 Hz, 2H), 4.63 − 4.49 (m, 2H). ^13^C NMR (151 MHz, CDCl_3_) δ 163.48, 151.65, 140.35, 132.09, 132.03, 127.16, 125.68, 124.11, 121.24, 116.13, 115.99, 111.46, 109.97, 59.68. ESI-HRMS: calcd for C_14_H_10_FNO_2_S [M + H]^+^, 276.0495; found, 276.0503.

2-((4-bromobenzyl) sulfinyl) benzo[*d*]oxazole (**5f**) white solid, yield 79%. ^1^H NMR (600 MHz, Chloroform-*d*) δ 7.84 − 7.80 (m, 1H), 7.64 − 7.60 (m, 1H), 7.50 − 7.40 (m, 4H), 7.13 − 7.09 (m, 2H), 4.62 − 4.38 (m, 2H). ^13^C NMR (151 MHz, CDCl_3_) δ 163.37, 151.64, 140.33, 132.15, 131.82, 127.24, 127.18, 125.70, 123.51, 121.26, 111.47, 59.80. ESI-HRMS: calcd for C_14_H_10_BrNO_2_S [M + Na]^+^, 357.9513; found, 357.9493.

2-((4-methylbenzyl) sulfinyl) benzo[*d*]oxazole (**5 g**) white solid, yield 70%. ^1^H NMR (600 MHz, Chloroform-*d*) δ 7.84 − 7.80 (m, 1H), 7.63 − 7.59 (m, 1H), 7.49 − 7.43 (m, 2H), 7.12 − 7.08 (m, 4H), 4.64 − 4.45 (m, 2H), 2.30 (s, 3H). ^13^C NMR (151 MHz, CDCl_3_) δ 163.87, 151.65, 140.44, 139.04, 130.13, 129.74, 127.05, 125.58, 125.05, 121.26, 111.47, 60.56, 21.21. ESI-HRMS: calcd for C_15_H_13_NO_2_S [M + H]^+^, 272.0745; found, 272.0745.

2-((naphthalen-2-ylmethyl) sulfinyl) benzo[*d*]oxazole (**5h**) yellow solid, yield 81%. ^1^H NMR (600 MHz, Chloroform-*d*) δ 7.82 − 7.76 (m, 2H), 7.75 − 7.70 (m, 2H), 7.58 (dd, *J* = 7.5, 1.7 Hz, 1H), 7.49 − 7.43 (m, 5H), 7.32 − 7.29 (m, 1H), 4.81 − 4.66 (m, 2H). ^13^C NMR (151 MHz, CDCl_3_) δ 163.83, 151.64, 140.41, 133.24, 133.23, 130.12, 128.85, 127.93, 127.68, 127.08, 126.70, 126.52, 125.67, 125.59, 121.23, 111.44, 61.13. ESI-HRMS: calcd for C_18_H_13_NO_2_S [M + Na]^+^, 330.0564; found, 308.0559.

2-((4-nitrobenzyl) sulfinyl) benzo[*d*]oxazole (**5i**) yellow solid, yield 87%. ^1^H NMR (600 MHz, Chloroform-*d*) δ 8.16 − 8.13 (m, 2H), 7.84 − 7.81 (m, 1H), 7.62 − 7.59 (m, 1H), 7.51 − 7.45 (m, 2H), 7.44 − 7.42 (m, 2H), 4.73 − 4.64 (m, 2H). ^13^C NMR (151 MHz, CDCl_3_) δ 162.83, 151.71, 148.27, 140.25, 135.56, 131.31, 127.43, 125.91, 123.96, 121.32, 111.51, 59.34. ESI-HRMS: calcd for C_14_H_10_N_2_O_4_S [M + H]^+^, 303.0440; found, 303.0440.

2-((4-(trifluoromethyl) benzyl) sulfinyl) benzo[*d*]oxazole (**5j**) white solid, yield 78%. ^1^H NMR (600 MHz, Chloroform-*d*) δ 7.84 (dd, *J* = 7.6, 1.5 Hz, 1H), 7.64 − 7.61 (m, 1H), 7.58 (d, *J* = 8.1 Hz, 2H), 7.52 − 7.46 (m, 2H), 7.39 (d, *J* = 8.0 Hz, 2H), 4.70 − 4.61 (m, 2H). ^13^C NMR (151 MHz, CDCl_3_) δ 163.18, 151.69, 140.32, 132.39, 131.29, 131.07, 130.68, 127.29, 125.90, 125.78, 123.98, 121.28, 111.49, 59.84. ESI-HRMS: calcd for C_15_H_10_F_3_NO_2_S [M + H]^+^, 326.0463; found, 326.0461.

2-((3-methoxybenzyl) sulfinyl) benzo[*d*]oxazole (**5k**) yellow solid, yield 69%. ^1^H NMR (600 MHz, Chloroform-*d*) δ 7.86 − 7.82 (m, 1H), 7.64 (dd, *J* = 7.9, 1.2 Hz, 1H), 7.48 (ddd, *J* = 12.9, 7.7, 1.3 Hz, 2H), 7.22 (d, *J* = 7.9 Hz, 1H), 6.88 − 6.83 (m, 2H), 6.75 (t, *J* = 2.1 Hz, 1H), 4.64 − 4.54 (m, 2H), 3.67 (s, 3H). ^13^C NMR (151 MHz, CDCl_3_) δ 163.81, 159.90, 151.64, 140.43, 130.05, 129.58, 127.11, 125.63, 122.50, 121.26, 115.18, 115.15, 111.48, 60.96, 55.12. ESI-HRMS: calcd for C_15_H_13_NO_3_S [M + H]^+^, 288.0694; found, 288.0695.

2-((4-methoxybenzyl) sulfinyl) benzo[*d*]oxazole (**5 l**) yellow solid, yield 63%. ^1^H NMR (600 MHz, Chloroform-*d*) δ 7.81 (dd, *J* = 7.6, 1.5 Hz, 1H), 7.63 − 7.59 (m, 1H), 7.45 (dtd, *J* = 19.3, 7.5, 1.3 Hz, 2H), 7.16 − 7.13 (m, 2H), 6.83 − 6.78 (m, 2H), 4.60 − 4.49 (m, 2H), 3.76 (s, 3H). ^13^C NMR (151 MHz, CDCl_3_) δ 163.88, 160.20, 151.64, 140.44, 131.49, 127.01, 125.56, 121.22, 119.96, 114.48, 111.45, 60.25, 55.27. ESI-HRMS: calcd for C_15_H_13_NO_3_S [M + Na]^+^, 310.0513; found, 310.0491.

2-(benzylsulfinyl)-5-chlorobenzo[*d*]oxazole (**6a**) white solid, yield 80%. ^1^H NMR (600 MHz, Chloroform-*d*) δ 7.80 (s, 1H), 7.55 − 7.50 (m, 1H), 7.44 (d, *J* = 8.8 Hz, 1H), 7.32 − 7.27 (m, 2H), 7.21 (d, *J* = 8.2 Hz, 2H), 7.11 − 7.06 (m, 1H), 4.87 − 4.37 (m, 2H). ^13^C NMR (151 MHz, CDCl_3_) δ 165.20, 150.21, 141.38, 131.29, 130.19, 129.15, 129.06, 127.97, 127.56, 121.15, 112.24, 60.81. ESI-HRMS: calcd for C_14_H_10_ClNO_2_S [M + H]^+^, 292.0199; found, 292.0198.

5-chloro-2-((4-chlorobenzyl) sulfinyl) benzo[*d*]oxazole (**6b**) white solid, yield 85%. ^1^H NMR (600 MHz, Chloroform-*d*) δ 7.80 (t, *J* = 1.5 Hz, 1H), 7.53 (dd, *J* = 8.8, 1.1 Hz, 1H), 7.45 (dt, *J* = 8.8, 1.6 Hz, 1H), 7.26 (s, 2H), 7.18 − 7.11 (m, 2H), 4.61 − 4.47 (m, 2H). ^13^C NMR (151 MHz, CDCl_3_) δ 164.95, 150.21, 141.35, 135.44, 131.51, 131.38, 129.25, 127.65, 126.52, 121.15, 112.27, 59.80. ESI-HRMS: calcd for C_14_H_9_Cl_2_NO_2_S [M + H]^+^, 325.9810; found, 325.9817.

5-chloro-2-((3-chlorobenzyl) sulfinyl) benzo[*d*]oxazole (**6c**) white solid, yield 85%. ^1^H NMR (600 MHz, Chloroform-*d*) δ 7.82 − 7.80 (m, 1H), 7.54 (d, *J* = 8.8 Hz, 1H), 7.45 (dd, *J* = 8.8, 2.1 Hz, 1H), 7.31 (ddd, *J* = 8.1, 2.0, 1.1 Hz, 1H), 7.25 − 7.22 (m, 2H), 7.12 (dt, *J* = 7.7, 1.3 Hz, 1H), 4.59 − 4.49 (m, 2H). ^13^C NMR (151 MHz, CDCl_3_) δ 164.93, 150.22, 141.33, 134.90, 131.39, 130.28, 130.24, 130.05, 129.34, 128.39, 127.69, 121.17, 112.27, 60.07. ESI-HRMS: calcd for C_14_H_9_Cl_2_NO_2_S [M + H]^+^, 325.9810; found, 325.9814.

5-chloro-2-((2-chlorobenzyl) sulfinyl) benzo[*d*]oxazole (**6d**) white solid, yield 82%. ^1^H NMR (600 MHz, Chloroform-*d*) δ 7.88 − 7.79 (m, 1H), 7.57 (dd, *J* = 8.8, 0.5 Hz, 1H), 7.46 (dd, *J* = 8.7, 2.1 Hz, 1H), 7.41 (dd, *J* = 7.9, 1.2 Hz, 1H), 7.32 − 7.30 (m, 1H), 7.22 (td, *J* = 7.5, 1.3 Hz, 1H), 7.10 − 7.08 (m, 1H), 4.80 (dd, *J* = 111.6, 12.8 Hz, 2H). ^13^C NMR (151 MHz, CDCl_3_) δ 165.30, 150.29, 134.98, 132.84, 131.36, 130.73, 130.00, 127.76, 127.39, 126.36, 122.55, 121.30, 112.30, 58.78. ESI-HRMS: calcd for C_14_H_9_Cl_2_NO_2_S [M + H]^+^, 325.9810; found, 325.9822.

5-chloro-2-((4-fluorobenzyl) sulfinyl) benzo[*d*]oxazole (**6e**) yellow solid, yield 79%. ^1^H NMR (600 MHz, Chloroform-*d*) δ 7.80 (t, *J* = 1.6 Hz, 1H), 7.53 (d, *J* = 8.7 Hz, 1H), 7.44 (ddd, *J* = 8.5, 2.1, 0.9 Hz, 1H), 7.23 − 7.16 (m, 2H), 7.04 − 6.94 (m, 2H), 4.63 − 4.42 (m, 2H). ^13^C NMR (151 MHz, CDCl_3_) δ 165.03, 164.08, 162.43, 150.21, 141.36, 132.00, 131.36, 127.61, 123.88, 121.13, 116.20, 116.05, 112.25, 59.73. ESI-HRMS: calcd for C_14_H_9_ClFNO_2_S [M + H]^+^, 310.0105; found, 310.0113.

2-((4-bromobenzyl) sulfinyl)-5-chlorobenzo[*d*]oxazole (**6f**) white solid, yield 81%. ^1^H NMR (600 MHz, Chloroform-*d*) δ 7.80 (d, *J* = 2.0 Hz, 1H), 7.54 (d, *J* = 8.8 Hz, 1H), 7.46 − 7.41 (m, 3H), 7.09 (d, *J* = 8.5 Hz, 2H), 4.63 − 4.37 (m, 2H). ^13^C NMR (151 MHz, CDCl_3_) δ 164.85, 150.21, 141.31, 132.20, 131.78, 131.39, 127.66, 126.95, 123.66, 121.15, 112.27, 59.81. ESI-HRMS: calcd for C_14_H_9_BrClNO_2_S [M + H]^+^, 369.9304; found, 369.9313.

5-chloro-2-((4-methylbenzyl) sulfinyl) benzo[*d*]oxazole (**6 g**) yellow solid, yield 80%. ^1^H NMR (600 MHz, Chloroform-*d*) δ 7.80 (d, *J* = 2.0 Hz, 1H), 7.53 (d, *J* = 8.7 Hz, 1H), 7.44 (dd, *J* = 8.7, 2.0 Hz, 1H), 7.09 (s, 4H), 4.61 − 4.49 (m, 2H), 2.30 (s, 3H). ^13^C NMR (151 MHz, CDCl_3_) δ 165.35, 150.19, 141.42, 139.17, 131.23, 130.08, 129.29, 127.50, 124.75, 121.14, 112.23, 60.57, 21.21. ESI-HRMS: calcd for C_15_H_12_ClNO_2_S [M + H]^+^, 306.0356; found, 306.0359.

5-chloro-2-((naphthalen-2-ylmethyl) sulfinyl) benzo[*d*]oxazole (**6h**) yellow solid, yield 87%. ^1^H NMR (600 MHz, Chloroform-*d*) δ 7.79 (q, *J* = 2.7, 2.3 Hz, 2H), 7.74 (d, *J* = 8.9 Hz, 3H), 7.52 − 7.45 (m, 3H), 7.43 (dd, *J* = 8.8, 2.1 Hz, 1H), 7.27 (dd, *J* = 8.4, 1.9 Hz, 1H), 4.83 − 4.66 (m, 2H). ^13^C NMR (151 MHz, CDCl_3_) δ 165.33, 150.21, 141.41, 133.28, 133.22, 131.28, 130.16, 128.93, 127.93, 127.70, 127.54, 126.97, 126.81, 126.63, 125.35, 121.13, 112.23, 61.18. ESI-HRMS: calcd for C_18_H_12_ClNO_2_S [M + Na]^+^, 364.0175; found, 364.0179.

5-chloro-2-((4-nitrobenzyl) sulfinyl) benzo[*d*]oxazole (**6i**) yellow solid, yield 72%. ^1^H NMR (600 MHz, Chloroform-*d*) δ 8.19 − 8.12 (m, 2H), 7.81 (d, *J* = 2.0 Hz, 1H), 7.53 (d, *J* = 8.8 Hz, 1H), 7.48 − 7.40 (m, 3H), 4.76 − 4.59 (m, 2H). ^13^C NMR (151 MHz, CDCl_3_) δ 164.38, 150.26, 148.33, 141.24, 135.33, 131.60, 131.30, 130.47, 127.89, 124.00, 121.21, 112.31, 59.34. ESI-HRMS: calcd for C_14_H_9_ClN_2_O_4_S [M + Na]^+^, 358.9869; found, 359.0133.

5-chloro-2-((4-(trifluoromethyl) benzyl) sulfinyl) benzo[*d*]oxazole (**6j**) white solid, yield 81%. ^1^H NMR (600 MHz, Chloroform-*d*) δ 7.81 (d, *J* = 2.0 Hz, 1H), 7.56 (d, *J* = 8.1 Hz, 2H), 7.52 (d, *J* = 8.7 Hz, 1H), 7.45 (dd, *J* = 8.8, 2.1 Hz, 1H), 7.36 (d, *J* = 8.0 Hz, 2H), 4.74 − 4.55 (m, 2H). ^13^C NMR (151 MHz, CDCl_3_) δ 164.73, 150.26, 141.32, 132.17, 131.49, 131.41, 130.68, 130.58, 129.80, 127.77, 125.94, 121.18, 112.28, 59.85. ESI-HRMS: calcd for C_15_H_9_ClF_3_NO_2_S [M + H]^+^, 360.0073; found, 360.0079.

5-chloro-2-((3-methoxybenzyl) sulfinyl) benzo[*d*]oxazole (**6k**) yellow solid, yield 75%. ^1^H NMR (600 MHz, Chloroform-*d*) δ 7.80 (d, *J* = 2.1 Hz, 1H), 7.54 (d, *J* = 8.7 Hz, 1H), 7.44 (dd, *J* = 8.7, 2.1 Hz, 1H), 7.19 (dd, *J* = 8.3, 7.5 Hz, 1H), 6.85 (ddd, *J* = 8.4, 2.6, 0.9 Hz, 1H), 6.79 (dt, *J* = 7.5, 1.2 Hz, 1H), 6.74 (t, *J* = 2.1 Hz, 1H), 4.60 − 4.51 (m, 2H), 3.68 (s, 3H). ^13^C NMR (151 MHz, CDCl_3_) δ 165.28, 159.92, 150.20, 141.39, 131.30, 130.08, 129.30, 127.57, 122.38, 121.13, 115.40, 115.05, 112.25, 60.91, 55.15. ESI-HRMS: calcd for C_15_H_12_ClNO_3_S [M + H]^+^, 322.0305; found, 322.0312.

5-chloro-2-((4-methoxybenzyl) sulfinyl) benzo[*d*]oxazole (**6 l**) white solid, yield 75%. ^1^H NMR (600 MHz, Chloroform-*d*) δ 7.79 (d, *J* = 2.1 Hz, 1H), 7.53 (d, *J* = 8.8 Hz, 1H), 7.44 (dd, *J* = 8.8, 2.1 Hz, 1H), 7.15 − 7.10 (m, 2H), 6.83 − 6.79 (m, 2H), 4.59 − 4.48 (m, 2H), 3.77 (s, 3H). ^13^C NMR (151 MHz, CDCl_3_) δ 165.41, 160.29, 150.20, 141.44, 131.47, 131.23, 127.47, 121.11, 119.68, 114.51, 112.23, 60.30, 55.28. ESI-HRMS: calcd for C_15_H_12_ClNO_3_S [M + Na]^+^, 344.0124; found, 344.0102.

2-(benzylsulfinyl)-6-chlorobenzo[*d*]oxazole (**7a**) white solid, yield 78%. ^1^H NMR (600 MHz, Chloroform-*d*) δ 7.73 (d, *J* = 8.6 Hz, 1H), 7.61 (d, *J* = 1.9 Hz, 1H), 7.42 (dd, *J* = 8.6, 1.9 Hz, 1H), 7.31 − 7.28 (m, 2H), 7.24 − 7.17 (m, 3H), 4.64 − 4.54 (m, 2H). ^13^C NMR (151 MHz, CDCl_3_) δ 164.36, 151.71, 139.07, 130.18, 129.14, 129.05, 127.94, 126.58, 124.08, 121.79, 112.04, 60.74. ESI-HRMS: calcd for C_14_H_10_ClNO_2_S [M + H]^+^, 292.0199; found, 292.0200.

6-chloro-2-((4-chlorobenzyl) sulfinyl) benzo[*d*]oxazole (**7b**) white solid, yield 85%. ^1^H NMR (600 MHz, Chloroform-*d*) δ 7.75 (d, *J* = 8.6 Hz, 1H), 7.65 (d, *J* = 1.8 Hz, 1H), 7.46 (dd, *J* = 8.6, 1.9 Hz, 1H), 7.29 (d, *J* = 8.6 Hz, 2H), 7.17 (dd, *J* = 13.7, 8.4 Hz, 2H), 4.62 − 4.53 (m, 2H). ^13^C NMR (151 MHz, CDCl_3_) δ 164.11, 151.74, 139.06, 135.45, 133.19, 131.51, 129.27, 126.69, 126.50, 121.81, 112.08, 59.76. ESI-HRMS: calcd for C_14_H_9_Cl_2_NO_2_S [M + H]^+^, 325.9809; found, 325.9812.

6-chloro-2-((4-methylbenzyl) sulfinyl) benzo[*d*]oxazole (**7c**) white solid, yield 82%. ^1^H NMR (600 MHz, Chloroform-*d*) δ 7.72 (d, *J* = 8.5 Hz, 1H), 7.61 (d, *J* = 1.8 Hz, 1H), 7.42 (dd, *J* = 8.6, 1.9 Hz, 1H), 7.09 (s, 4H), 4.61 − 4.37 (m, 2H), 2.30 (s, 3H). ^13^C NMR (151 MHz, CDCl_3_) δ 164.55, 151.69, 139.15, 132.97, 130.07, 129.76, 126.52, 124.75, 123.87, 121.77, 112.03, 60.51, 21.20. ESI-HRMS: calcd for C_15_H_12_ClNO_2_S [M + H]^+^, 306.0277; found, 306.0360.

6-chloro-2-((4-nitrobenzyl) sulfinyl) benzo[*d*]oxazole (**7d**) yellow solid, yield 86%. ^1^H NMR (600 MHz, Chloroform-*d*) δ 8.19 − 8.13 (m, 2H), 7.74 (d, *J* = 8.6 Hz, 1H), 7.63 (d, *J* = 1.9 Hz, 1H), 7.44 (dd, *J* = 13.5, 9.6 Hz, 3H), 4.71 − 4.62 (m, 2H). ^13^C NMR (151 MHz, CDCl_3_) δ 163.58, 151.78, 148.33, 138.97, 135.34, 133.43, 131.29, 126.88, 124.02, 121.85, 112.12, 59.32. ESI-HRMS: calcd for C_14_H_9_ClN_2_O_4_S [M + H]^+^, 337.0050; found, 337.0054.

6-chloro-2-((4-(trifluoromethyl) benzyl) sulfinyl) benzo[*d*]oxazole (**7e**) white solid, yield 77%. ^1^H NMR (600 MHz, Chloroform-*d*) δ 7.73 (d, *J* = 8.6 Hz, 1H), 7.61 (d, *J* = 1.9 Hz, 1H), 7.56 (d, *J* = 8.0 Hz, 2H), 7.44 (dd, *J* = 8.5, 1.9 Hz, 1H), 7.37 (d, *J* = 8.0 Hz, 2H), 4.70 − 4.58 (m, 2H). ^13^C NMR (151 MHz, CDCl_3_) δ 163.87, 151.77, 139.01, 133.31, 132.14, 130.68, 126.78, 125.95, 124.09, 121.83, 112.08, 110.94, 110.53, 59.77. ESI-HRMS: calcd for C_15_H_9_ClF_3_NO_2_S [M + H]^+^, 360.0073; found, 360.0071.

6-chloro-2-((4-fluorobenzyl) sulfinyl) benzo[*d*]oxazole (**7f**) yellow solid, yield 80%. ^1^H NMR (600 MHz, Chloroform-*d*) δ 7.73 (d, *J* = 8.6 Hz, 1H), 7.62 (t, *J* = 1.4 Hz, 1H), 7.43 (dt, *J* = 8.6, 1.4 Hz, 1H), 7.22 − 7.20 (m, 2H), 6.98 (d, *J* = 8.4 Hz, 2H), 4.62 − 4.43 (m, 2H). ^13^C NMR (151 MHz, CDCl_3_) δ 164.16, 162.42, 151.73, 139.05, 133.15, 132.05, 132.00, 126.66, 124.11, 121.79, 116.22, 116.07, 112.06, 59.69. ESI-HRMS: calcd for C_14_H_9_ClFNO_2_S [M + H]^+^, 310.0105; found, 310.0108.

2-(benzylsulfinyl)-5-methylbenzo[*d*]oxazole (**8a**) white solid, yield 79%. ^1^H NMR (600 MHz, Chloroform-*d*) δ 7.58 (s, 1H), 7.46 (d, *J* = 8.4 Hz, 1H), 7.32 − 7.26 (m, 3H), 7.26 − 7.21 (m, 2H), 6.86 (d, *J* = 10.2 Hz, 1H), 4.62 − 4.54 (m, 2H), 2.49 (s, 3H). ^13^C NMR (151 MHz, CDCl_3_) δ 156.28, 141.94, 134.09, 130.25, 129.44, 129.00, 128.39, 123.04, 120.96, 110.80, 110.63, 109.61, 60.71, 21.43. ESI-HRMS: calcd for C_15_H_13_NO_2_S [M + H]^+^, 272.0745; found, 272.0740.

2-((4-chlorobenzyl) sulfinyl)-5-methylbenzo[*d*]oxazole (**8b**) white solid, yield 87%. ^1^H NMR (600 MHz, Chloroform-*d*) δ 7.60 − 7.56 (m, 1H), 7.46 (d, *J* = 8.4 Hz, 1H), 7.29 − 7.22 (m, 3H), 7.15 (d, *J* = 8.4 Hz, 2H), 4.60 − 4.50 (m, 2H), 2.49 (s, 3H). ^13^C NMR (151 MHz, CDCl_3_) δ 163.26, 149.94, 140.52, 135.78, 135.21, 131.54, 129.16, 128.43, 126.80, 120.94, 110.79, 59.67, 21.48. ESI-HRMS: calcd for C_15_H_12_ClNO_2_S [M + H]^+^, 306.0356; found, 306.0344.

5-methyl-2-((4-methylbenzyl) sulfinyl) benzo[*d*]oxazole (**8c**) white solid, yield 84%. ^1^H NMR (600 MHz, Chloroform-*d*) δ 7.47 (d, *J* = 8.4 Hz, 1H), 7.29 − 7.24 (m, 1H), 7.10 (t, *J* = 8.9 Hz, 4H), 6.87 (d, *J* = 7.9 Hz, 1H), 4.60 − 4.52 (m, 2H), 2.49 (s, 3H), 2.29 (s, 3H). ^13^C NMR (151 MHz, CDCl_3_) δ 163.69, 149.96, 140.60, 138.99, 135.67, 10.15, 129.72, 128.34, 125.05, 120.96, 110.80, 60.45, 21.50, 21.19. ESI-HRMS: calcd for C_16_H_15_NO_2_S [M + H]^+^, 286.0902; found, 286.0896.

5-methyl-2-((4-nitrobenzyl) sulfinyl) benzo[*d*]oxazole (**8d**) yellow solid, yield 81%. ^1^H NMR (600 MHz, Chloroform-*d*) δ 8.19 − 8.09 (m, 2H), 7.58 (s, 1H), 7.46 (d, *J* = 8.5 Hz, 1H), 7.41 (d, *J* = 8.7 Hz, 2H), 7.29 (dd, *J* = 8.5, 1.7 Hz, 1H), 4.93 − 4.30 (m, 2H), 2.51 (s, 3H). ^13^C NMR (151 MHz, CDCl_3_) δ 162.69, 150.00, 148.23, 140.45, 136.04, 135.62, 131.28, 128.66, 120.99, 110.82, 59.31, 21.50. ESI-HRMS: calcd for C_15_H_12_N_2_O_4_S [M + H]^+^, 317.0596; found, 317.0588.

5-methyl-2-((4-(trifluoromethyl) benzyl) sulfinyl) benzo[*d*]oxazole (**8e**) white solid, yield 73%. ^1^H NMR (600 MHz, Chloroform-*d*) δ 7.60 − 7.58 (m, 1H), 7.54 (s, 1H), 7.46 (d, *J* = 8.5 Hz, 1H), 7.36 (d, *J* = 8.0 Hz, 2H), 7.28 (dd, *J* = 8.4, 1.7 Hz, 1H), 4.67 − 4.55 (m, 2H), 2.50 (s, 3H). ^13^C NMR (151 MHz, CDCl_3_) δ 163.08, 150.01, 140.54, 135.90, 132.46, 131.25, 131.03, 130.68, 128.54, 125.88, 120.97, 110.82, 59.81, 21.50. ESI-HRMS: calcd for C_16_H_12_F_3_NO_2_S [M + H]^+^, 340.0619; found, 340.0602.

2-((4-fluorobenzyl) sulfinyl)-5-methylbenzo[*d*]oxazole (**8f**) white solid, yield 79%. ^1^H NMR (600 MHz, Chloroform-*d*) δ 7.58 (dt, *J* = 1.7, 0.8 Hz, 1H), 7.47 (d, *J* = 8.4 Hz, 1H), 7.29 − 7.27 (m, 1H), 7.23 − 7.17 (m, 2H), 6.97 (t, *J* = 8.6 Hz, 2H), 4.62 − 4.46 (m, 2H), 2.49 (s, 3H). ^13^C NMR (151 MHz, CDCl_3_) δ 164.00, 163.37, 162.35, 149.95, 140.56, 135.77, 132.07, 132.02, 128.40, 120.93, 116.11, 115.97, 110.79, 59.65, 21.49. ESI-HRMS: calcd for C_15_H_12_FNO_2_S [M + H]^+^, 290.0651, found, 290.0642.

2-(benzylsulfinyl)-5-methoxybenzo[*d*]oxazole (**9a**) white solid, yield 81%. ^1^H NMR (600 MHz, Chloroform-*d*) δ 7.49 − 7.46 (m, 1H), 7.32 − 7.27 (m, 3H), 7.24 − 7.22 (m, 3H), 7.05 (dd, *J* = 9.0, 2.6 Hz, 1H), 4.67 − 4.48 (m, 2H), 3.86 (s, 3H). ^13^C NMR (151 MHz, CDCl_3_) δ 164.16, 157.97, 146.34, 141.29, 130.22, 128.99, 128.96, 128.34, 116.33, 111.65, 103.32, 60.75, 55.97. ESI-HRMS: calcd for C_15_H_13_NO_3_S [M + H]^+^, 288.0694; found, 288.0673.

2-((4-chlorobenzyl) sulfinyl)-5-methoxybenzo[*d*]oxazole (**9b**) white solid, yield 88%. ^1^H NMR (600 MHz, Chloroform-*d*) δ 7.48 (dd, *J* = 9.0, 1.3 Hz, 1H), 7.30 − 7.22 (m, 3H), 7.17 − 7.14 (m, 2H), 7.07 (dd, *J* = 8.9, 2.5 Hz, 1H), 4.62 − 4.45 (m, 2H), 3.88 (d, *J* = 1.4 Hz, 3H). ^13^C NMR (151 MHz, CDCl_3_) δ 163.76, 158.06, 146.38, 141.25, 135.29, 131.54, 129.21, 126.76, 116.49, 111.69, 103.32, 59.75, 56.00. ESI-HRMS: calcd for C_15_H_12_ClNO_3_S [M + H]^+^, 322.0305; found, 322.0295.

5-methoxy-2-((4-methylbenzyl) sulfinyl) benzo[*d*]oxazole (**9c**) white solid, yield 85%. ^1^H NMR (600 MHz, Chloroform-*d*) δ 7.48 (d, *J* = 9.0 Hz, 1H), 7.24 (d, *J* = 2.5 Hz, 1H), 7.12 − 7.04 (m, 5H), 4.65 − 4.42 (m, 2H), 3.87 (s, 3H), 2.30 (s, 3H). ^13^C NMR (151 MHz, CDCl_3_) δ 164.32, 157.97, 146.36, 141.34, 138.97, 130.12, 129.72, 125.11, 116.30, 111.66, 103.34, 60.53, 55.98, 21.20. ESI-HRMS: calcd for C_16_H_15_NO_3_S [M + H]^+^, 302.0851; found, 302.0833.

5-methoxy-2-((4-nitrobenzyl) sulfinyl) benzo[*d*]oxazole (**9d**) yellow solid, yield 81%. ^1^H NMR (600 MHz, Chloroform-*d*) δ 8.18 − 8.11 (m, 2H), 7.47 (d, *J* = 9.0 Hz, 1H), 7.44 − 7.39 (m, 2H), 7.24 (d, *J* = 2.5 Hz, 1H), 7.07 (dd, *J* = 9.1, 2.4 Hz, 1H), 4.70 − 4.60 (m, 2H), 3.88 (s, 3H). ^13^C NMR (151 MHz, CDCl_3_) δ 163.13, 158.16, 148.23, 146.40, 141.16, 135.57, 131.29, 123.95, 116.70, 111.72, 103.31, 59.32, 56.01. ESI-HRMS: calcd for C_15_H_12_N_2_O_5_S [M + H]^+^, 333.0545; found, 333.0545.

5-methoxy-2-((4-(trifluoromethyl) benzyl) sulfinyl) benzo[*d*]oxazole (**9e**) white solid, yield 83%. ^1^H NMR (600 MHz, Chloroform-*d*) δ 7.60 − 7.54 (m, 2H), 7.49 (d, *J* = 9.0 Hz, 1H), 7.38 (d, *J* = 8.0 Hz, 2H), 7.26 (d, *J* = 2.5 Hz, 1H), 7.11 − 7.08 (m, 1H), 4.69 − 4.57 (m, 2H), 3.89 (s, 3H). ^13^C NMR (151 MHz, CDCl_3_) δ 163.56, 158.12, 146.42, 141.24, 132.44, 131.27, 131.05, 130.68, 125.87, 116.58, 111.70, 103.33, 59.84, 56.00. ESI-HRMS: calcd for C_16_H_12_F_3_NO_3_S [M + H]^+^, 356.0568; found, 356.0551.

2-((4-fluorobenzyl) sulfinyl)-5-methoxybenzo[*d*]oxazole (**9f**) white solid, yield 79%. ^1^H NMR (600 MHz, Chloroform-*d*) δ 7.48 (d, *J* = 9.0 Hz, 1H), 7.24 (d, *J* = 2.6 Hz, 1H), 7.23 − 7.18 (m, 2H), 7.06 (dd, *J* = 9.0, 2.6 Hz, 1H), 6.98 (t, *J* = 8.5 Hz, 2H), 4.61 − 4.49 (m, 2H), 3.87 (s, 3H). ^13^C NMR (151 MHz, CDCl_3_) δ 164.02, 163.87, 162.37, 158.04, 146.37, 141.27, 132.07, 124.12, 116.42, 116.13, 115.99, 111.67, 103.31, 59.69, 55.99. ESI-HRMS: calcd for C_15_H_12_FNO_3_S [M + H]^+^, 306.0600; found, 306.0588.

### Biological investigations

#### Biofilm formation assessment

Biofilms were quantitatively detected by the crystal violet method.^35^ The overnight culture of *P. aeruginosa* PAO1 was diluted 1000 times with fresh Luria broth medium. 199 μL of the diluted bacterial solution was added to a 96-well plate, and 1 μL of the 10 mM compound stock solution prepared was added to make the final concentration of the compound 50 μM. The same volume of DMSO was added as a negative control. Then the 96-well plates were incubated at 37 °C for 24 h. Then the OD value at 600 nm was measured by a microplate reader. After removing the upper layer of bacteria, rinse gently 2–3 times with PBS buffer. After dehydration and fixation at 37 °C, 200 μL of 0.1% crystal violet solution was added. After standing for 15 min for staining, the excess crystal violet solution was removed by suction and the remaining crystal violet was gently rinsed. After drying at 37 °C, the pigment was dissolved in 200 μL of 95% ethanol. Finally, the absorbance was measured at 595 nm by the microplate reader. The formula for calculating the biofilm inhibition rate is: OD_595control_-OD_595_/OD_595control_ × 100%.

#### Growth curve analysis

*P. aeruginosa* PAO1 was cultured overnight, diluted to OD_600_ = 0.01, and then transferred into 96-well plates with active compounds (to give a final concentration of 50 μM, 20 μM, 10 μM, 5 μM). Bacterial cultures were incubated for at 37 °C, and optical density was measured at 600 nm every hour for 16 h.

#### CLSM images

*P. aeruginosa* PAO1was cultured overnight and diluted 100 times, the medium and compounds were added to the plate to make the final concentration 50 μM, 25 μM. The mixture was incubated at 37 °C for 24 h. Supernatants were poured out and washed with water for three times to remove the floating bacteria, then fixed with 4% paraformaldehyde for 15 min, after that stained with 0.01% acridine orange for 15 min (in the dark), and the excess dye was washed away with PBS. The established model was observed by laser confocal microscope (LEICA, TCS SP8). The excitation filter wavelength was detected at 488 nm and the blocking filter wavelength was detected at 515 nm. The signal was received by FITC channel with an objective lens ×63.

#### GFP reporter strain assay

Compounds were prepared as stock solutions at concentration of 10 mM in 100% dimethyl sulfoxide. The PAO1*-1asB-gfp* strain was grown in LB medium at 37 °C, 200 rpm for 12–16 h, and the culture was diluted in ABTGC medium to a final optical density of 600 nm (OD_600_) of 0.02 (2.5 × 10 CFU/mL). Next, the compound stock solution and the diluted bacterial suspension were added to a 96-well microtiter plate to a final concentration of 20 μM. The same amount of a 0.02% DMSO solvent control group was set. The 96-well microtiter plates were incubated in a Molecular Devices SpectraMax microplate reader at 37 °C for at least 12 h, with OD_600_ and GFP fluorescence signals (excitation 485 nm, emission 535 nm) measured every 20 min. Inhibition assays for all test compounds and controls were performed in triplicate. The test methods of PAO1-*rhl*-*gfp* and PAO1-*pqs*-*gfp* strains are as above.^23^^,^^36^

#### Elastase assay

*P. aeruginosa* PAO1 was grown overnight and diluted to a density of 600 nm (OD_600_) of 0.01 in LB medium. Compounds were added to various final concentrations of 50 μM, 25 μM, 12.5 μM. Then incubate at 37 °C, shaking at 200 rpm for 24 h. Cultures were centrifuged at 10 000 rpm for 10 min. Aspirate the supernatant and filter sterilise with a disposable filter. The supernatant fraction 100 µL was incubated with 900 µL 2 mg/mL Elastin-Congo Red (ECR) prepared in 0.1mMTris-HCl, the reaction was shaken at 37 °C for 18 h. The reaction was then placed on ice, 100 μL of 0.12 M EDTA was added to terminate the reaction, and centrifuged at 4 °C, 12 000 r/min for 10 min to remove insoluble ECR; the absorbance of the supernatant was measured at 495 nm by a microplate reader.^37–38^

#### Pyocyanin quantification assay

The determination of pyocyanin is based on the absorbance of pyocyanin at 520 nm in acidic solution.^39^ *P. aeruginosa* PAO1 overnight culture was diluted with LB medium to OD600 = 0.01. Compounds were added to make a final concentration of 50 μM, 25 μM, 12.5 μM. Incubate at 37 °C, with shaking at 200 rpm for 24 h. The culture was then centrifuged at 10 000 rpm for 10 min, and 7.5 mL of the supernatant was transferred to a new centrifuge tube. Add 3 mL of chloroform for extraction (the chloroform layer turns blue); transfer the chloroform layer to a new centrifuge tube, add 1.5 mL of 0.2 M HCl for extraction (they turn pink after the hydrochloric acid mixed reaction). The HCl layer was pipetted into a microtiter plate and the absorbance was measured at 520 nm by Microplate reader.

#### Rhamnolipid quantification assay

Rhamnolipid production was directly quantified using the orcinol assay according to original protocol by Koch et al.^40^ *P. aeruginosa* PAO1 was diluted 100 times from overnight culture into fresh Luria broth medium (OD_600_=0.01). Compounds were then added to a final concentration of 50 μM, 25 μM, 12.5 μM. The cultures were grown for 24 h at 37 °C, shaking condition (200 rpm). Supernatants were collected by centrifuging at 10 000 rpm for 10 min and extracted with diethyl ether (twice). Organic fractions were concentrated to yield white solids. It was then resuspended in deionised water and added with 0.19% (w/v) orcinol in 50% H_2_SO_4_. The resulting mixture was incubated at 80 °C for 30 min to give orange solution. After cooling to room temperature, the absorbance was measured at 421 nm by a microplate reader.

#### Molecular docking

Molecules (OdDHL, **6b**) were drawn with ChemBioDraw Ultra 13.0 software and minimised with MOE (Molecular Operate Environment, 2020) software (Innovation Centre of Pesticide Research, Department of Applied Chemistry, College of Science, China Agricultural University, Beijing, China). The receptor protein LasR in PDB format downloaded from RCSB Protein Data Bank (http://www.pdb.org). LasR X-ray crystal structure with 1.80 A° resolution (PDB ID: 2UV0) was used for docking study.^41^ And it was prepared by the process of deleting water, adding hydrogen, adding Gasteiger charges and so on by Autodock Tools 15.6 software. The ligand binding site was defined using the bound ligands in the crystal structures. The OdDHL-binding pocket was selected as docking sites and docking environment was set in the solvent. Docking was operated by MOE after setting method (placement: triangle matcher, refinement: rigid receptor), score (placement: London dG, refinement: GBVI/WSA dG) and poses (placement: 30, refinement: 5). The best docking poses were selected to analyse the interaction between LasR and target compounds.

## Supplementary Material

Supplemental Material

## References

[1] Kalia VC, Rani A, Lal S, Cheema S, Raut CP. Combing databases reveals potential antibiotic producers. Expert Opin Drug Discov. 2007;2(2):211–224. 23496078 10.1517/17460441.2.2.211

[2] Kalia VC. Quorum sensing inhibitors: an overview. Biotechnol Adv. 2013;31(2):224–245. 23142623 10.1016/j.biotechadv.2012.10.004

[3] Lewis K. Persister cells, dormancy and infectious disease. Nat Rev Microbiol. 2007;5(1):48–56. 17143318 10.1038/nrmicro1557

[4] de Kievit TR. Quorum sensing in *Pseudomonas aeruginosa* biofilms. Environ Microbiol. 2009;11(2):279–288. 19196266 10.1111/j.1462-2920.2008.01792.x

[5] Costerton JW, Stewart PS, Greenberg EP. Bacterial biofilms: a common cause of persistent infections. Science. 1999;284 (5418):1318–1322. 10334980 10.1126/science.284.5418.1318

[6] Hentzer M, Givskov M. Pharmacological inhibition of quorum sensing for the treatment of chronic bacterial infections. J Clin Invest. 2003;112 (9):1300–1307. 14597754 10.1172/JCI20074 PMC228474

[7] Persson T, Givskov M, Nielsen J. Quorum sensing inhibition: targeting chemical communication in gram-negative bacteria. Curr Med Chem. 2005;12(26):3103–3115. 16375704 10.2174/092986705774933425

[8] Brackman G, Cos P, Maes L, Nelis HJ, Coenye T. Quorum sensing inhibitors increase the susceptibility of bacterial biofilms to antibiotics *in vitro* and *in vivo*. Antimicrob Agents Chemother. 2011;55(6):2655–2661. 21422204 10.1128/AAC.00045-11 PMC3101409

[9] LaSarre B, Federle MJ. Exploiting quorum sensing to confuse bacterial pathogens. Microbiol Mol Biol Rev. 2013;77(1):73–111. 23471618 10.1128/MMBR.00046-12 PMC3591984

[10] Lee J, Zhang L. The hierarchy quorum sensing network in *Pseudomonas aeruginosa*. Protein Cell. 2015;6(1):26–41. 25249263 10.1007/s13238-014-0100-x PMC4286720

[11] Soukarieh F, Williams P, Stocks MJ, Camara M. *Pseudomonas aeruginosa* quorum sensing systems as drug discovery targets: current position and future perspectives. J Med Chem. 2018;61(23):10385–10402. 29999316 10.1021/acs.jmedchem.8b00540

[12] Papaioannou E, Utari PD, Quax WJ. Choosing an appropriate infection model to study quorum sensing inhibition in *Pseudomonas* Infections. Int J Mol Sci. 2013;14(9):19309–19340. 24065108 10.3390/ijms140919309 PMC3794835

[13] Haussler S, Becker T. The *Pseudomonas* quinolone signal (PQS) balances life and death in *Pseudomonas aeruginosa* populations. PLoS Pathog. 2008;4(9):e1000166. 18818733 10.1371/journal.ppat.1000166 PMC2533401

[14] Schuster M, Lostroh CP, Ogi T, Greenberg EP. Identification, timing, and signal specificity of *Pseudomonas aeruginosa* quorum-controlled genes: a transcriptome analysis. J Bacteriol. 2003;185(7):2066–2079. 12644476 10.1128/JB.185.7.2066-2079.2003 PMC151497

[15] Zou Y, Nair SK. Molecular basis for the recognition of structurally distinct autoinducer mimics by the *Pseudomonas aeruginosa* LasR quorum-sensing signaling receptor. Chem Biol. 2009;16(9):961–970. 19778724 10.1016/j.chembiol.2009.09.001 PMC2778763

[16] Ochsner UA, Reiser J. Autoinducer-mediated regulation of rhamnolipid biosurfactant synthesis in *Pseudomonas aeruginosa*. Proc Natl Acad Sci USA. 1995;92(14):6424–6428. 7604006 10.1073/pnas.92.14.6424 PMC41530

[17] Ilangovan A, Fletcher M, Rampioni G, Pustelny C, Rumbaugh K, Heeb S, Camara M, Truman A, Chhabra SR, Emsley J, et al. Structural basis for native agonist and synthetic inhibitor recognition by the *Pseudomonas aeruginosa* quorum sensing regulator PqsR (MvfR). PLoS Pathog. 2013;9(7):e1003508. 23935486 10.1371/journal.ppat.1003508 PMC3723537

[18] Chugani SA, Whiteley M, Lee KM, D’Argenio D, Manoil C, Greenberg EP. QscR, a modulator of quorum-sensing signal synthesis and virulence in *Pseudomonas aeruginosa*. Proc Natl Acad Sci USA. 2001;98(5):2752–2757. 11226312 10.1073/pnas.051624298 PMC30211

[19] Preston MJ, Seed PC, Toder DS, Iglewski BH, Ohman DE, Gustin JK, Goldberg JB, Pier GB. Contribution of proteases and LasR to the virulence of *Pseudomonas aeruginosa* during corneal infections. Infect Immun. 1997;65(8):3086–3090. 9234758 10.1128/iai.65.8.3086-3090.1997 PMC175435

[20] Kalia VC, Purohit HJ. Quenching the quorum sensing system: potential antibacterial drug targets. Crit Rev Microbiol. 2011;37(2):121–140. 21271798 10.3109/1040841X.2010.532479

[21] Bjarnsholt T, van Gennip M, Jakobsen TH, Christensen LD, Jensen PØ, Givskov M. *In vitro* screens for quorum sensing inhibitors and *in vivo* confirmation of their effect. Nat Protoc. 2010;5(2):282–293. 20134428 10.1038/nprot.2009.205

[22] Jakobsen TH, van Gennip M, Phipps RK, Shanmugham MS, Christensen LD, Alhede M, Skindersoe ME, Rasmussen TB, Friedrich K, Uthe F, et al. Ajoene, a sulfur-rich molecule from garlic, inhibits genes controlled by quorum sensing. Antimicrob Agents Chemother. 2012;56(5):2314–2325. 22314537 10.1128/AAC.05919-11 PMC3346669

[23] Fong J, Yuan M, Jakobsen TH, Mortensen KT, Delos Santos MMS, Chua SL, Yang L, Tan CH, Nielsen TE, Givskov M. Disulfide bond-containing ajoene analogues as novel quorum sensing inhibitors of *Pseudomonas aeruginosa*. J Med Chem. 2017;60(1):215–227. 27977197 10.1021/acs.jmedchem.6b01025

[24] Gong Z, Peng Y, Qiu J, Cao A, Wang G, Peng Z. Synthesis, in vitro *α*-Glucosidase inhibitory activity and molecular docking studies of novel benzothiazole-triazole derivatives. Molecules. 2017;22(9):1555. 28914795 10.3390/molecules22091555 PMC6151782

[25] He M, Fan M, Liu W, Li Y, Wang G. Design, synthesis, molecular modeling, and biological evaluation of novel kojic acid derivatives containing bioactive heterocycle moiety as inhibitors of tyrosinase and antibrowning agents. Food Chem. 2021;362:130241. 34118508 10.1016/j.foodchem.2021.130241

[26] Ye X, Moeljadi AMP, Chin KF, Hirao H, Zong L, Tan CH. Enantioselective sulfoxidation catalyzed by a bisguanidinium diphosphatobisperoxotungstate ion pair. Angew Chem Int Ed Engl. 2016;55(25):7101–7105. 27150978 10.1002/anie.201601574

[27] Zhu JX, Lu Y, Chen J, Chen JW, Zhang H, Bao X, Ye X, Wang H. Total synthesis of quinolactacin-H from marine-derived Penicillium sp. ENP701 and biologic activities. RSC Adv. 2020;10(41):24251–24254. 35516178 10.1039/d0ra05244b PMC9055059

[28] Frei R, Breitbach AS, Blackwell HE. 2-Aminobenzimidazole derivatives strongly inhibit and disperse *Pseudomonas aeruginosa* biofilms. Angew Chem Int Ed Engl. 2012;51(21):5226–5229. 22488868 10.1002/anie.201109258 PMC3517033

[29] Rasmussen TB, Bjarnsholt T, Skindersoe ME, Hentzer M, Kristoffersen P, Köte M, Nielsen J, Eberl L, Givskov M. Screening for quorum-sensing inhibitors (QSI) by use of a novel genetic system, the QSI selector. J Bacteriol. 2005;187(5):1799–1814. 15716452 10.1128/JB.187.5.1799-1814.2005 PMC1063990

[30] Hentzer M, Riede K, Rasmussen TB, Heydorn A, Andersen JB, Parsek MR, Rice SA, Eber L, Molin S, Høiby N, et al. Inhibition of quorum sensing in *Pseudomonas aeruginosa* biofilm bacteria by a halogenated furanone compound. Microbiol. 2002;148(Pt 1):87–102. 10.1099/00221287-148-1-87 11782502

[31] Yang L, Rybtke MT, Jakobsen TH, Hentzer M, Bjarnsholt T, Givskov M, Tolker-Nielsen T. Computer-aided identification of recognized drugs as *Pseudomonas aeruginosa* quorum-sensing inhibitors. Antimicrob Agents Chemother. 2009;53(6):2432–2443. 19364871 10.1128/AAC.01283-08 PMC2687250

[32] Passador L, Cook JM, Gambello MJ, Rust L, Iglewski BH. Expression of *Pseudomonas aeruginosa* virulence genes requires cell-to-cell communication. Science. 1993;260(5111):1127–1130. 8493556 10.1126/science.8493556

[33] Williams P, Camara M, Hardman A, Swift S, Milton D, Hope VJ, Winzer K, Middleton B, Pritchard DI, Bycroft BW. Quorum sensing and the population-dependent control of virulence. Phil. Trans R Soc Lond B. 2000;355(1397):667–680. 10874739 10.1098/rstb.2000.0607 PMC1692775

[34] Gallagher LA, McKnight SL, Kuznetsova MS, Pesci EC, Manoil C. Functions required for extracellular quinolone signaling by *Pseudomonas aeruginosa*. J Bacteriol. 2002;184(23):6472–6480. 12426334 10.1128/JB.184.23.6472-6480.2002 PMC135424

[35] Christensen GD, Simpson WA, Younger JJ, Baddour LM, Barrett FF, Melton DM, Beachey EH. Adherence of coagulase-negative staphylococci to plastic tissue culture plates: a quantitative model for the adherence of staphylococci to medical devices. J Clin Microbiol. 1985;22(6):996–1006. 3905855 10.1128/jcm.22.6.996-1006.1985 PMC271866

[36] Tan SYY, Chua SL, Chen Y, Rice SA, Kjelleberg S, Nielsen TE, Yang L, Givskov M. Identification of five structurally unrelated quorum-sensing inhibitors of *Pseudomonas aeruginosa* from a natural-derivative database. Antimicrob Agents Chemother. 2013;57(11):5629–5641. 24002091 10.1128/AAC.00955-13 PMC3811257

[37] Hamood AN, Griswold J, Colmer J. Characterization of elastase-deficient clinical isolates of *Pseudomonas aeruginosa*. Infect Immun. 1996;64(8):3154–3160. 8757847 10.1128/iai.64.8.3154-3160.1996 PMC174201

[38] Smith K, Rajendran R, Kerr S, Lappin DF, Mackay WG, Williams C, Ramage G. Aspergillus fumigatus enhances elastase production in *Pseudomonas aeruginosa* co-cultures. Med Mycol. 2015;53(7):645–655. 26162475 10.1093/mmy/myv048

[39] Essar DW, Eberly L, Hadero A, Crawford IP. Identification and characterization of genes for a second anthranilate synthase in *Pseudomonas aeruginosa*: interchangeability of the two anthranilate synthases and evolutionary implications. J Bacteriol. 1990;172(2):884–900. 2153661 10.1128/jb.172.2.884-900.1990 PMC208517

[40] Koch AK, Kappeli O, Fiechter A, Reiser J. Hydrocarbon assimilation and biosurfactant production in *Pseudomonas aeruginosa* mutants. J Bacteriol. 1991;173(13):4212–4219. 1648079 10.1128/jb.173.13.4212-4219.1991 PMC208072

[41] Bottomley MJ, Muraglia E, Bazzo R, Carfi A. Molecular insights into quorum sensing in the human pathogen *Pseudomonas aeruginosa* from the structure of the virulence regulator LasR bound to its autoinducer. J Biol Chem. 2007;282(18):13592–13600. 17363368 10.1074/jbc.M700556200

