# Supplementary material for: Design, synthesis, and biological evaluation of benzoheterocyclic sulfoxide derivatives as quorum sensing inhibitors in Pseudomonas aeruginosa
Source: J Enzyme Inhib Med Chem. 2023 Feb 7;38(1):2175820. doi: 10.1080/14756366.2023.2175820 (PMC9930800; doi:10.1080/14756366.2023.2175820)
Supplement: Supplemental Material [file IENZ_A_2175820_SM5143.pdf]

Supporting Information

Design, synthesis and biological evaluation of benzoheterocyclic sulfoxide derivatives as quorum sensing inhibitors in *Pseudomonas aeruginosa*

Shen Mao,[a] Qiaoqiang Li,[a] Zhikun Yang,[a] Yasheng Li,[b] Xinyi Ye,[a,*] Hong Wang[a,*]

[a] College of Pharmaceutical Science & Green Pharmaceutical Collaborative Innovation Center of Yangtze River Del-ta Region, Zhejiang University of Technology, Hangzhou 310014, China

[b] Department of Infectious Diseases & Anhui Center for Surveillance of Bacterial Resistance, The First Affiliated Hospital of Anhui Medical University, Hefei, Anhui, China

*Corresponding author.

E-mail address: xinyiye1020@zjut.edu.cn (X.Y. Ye), hongw@zjut.edu.cn (H. Wang).

Pages S2-S4:

Table 1-4 Biofilm inhibition rates of derivatives against the *P. aeruginosa* PAO1.

Pages S5:

Figure S-1 Cytotoxicity of **6b**.

Pages S6-S59:

Figures S1-S54 ^{1}H NMR and ^{13}C NMR spectra of title target compounds **4a-4l**, **5a-5l**, **6a-6l**, **7a-7f**, **8a-8f**, **9a-9f.**

Table 1 Biofilm inhibition rates of derivatives against the *P. aeruginosa* PAO1.

**Entry**	**Compd.**	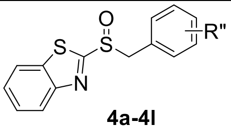 **4a-4l**			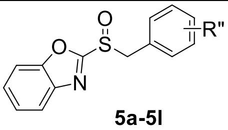 **5a-5l**		
		**Inhibition rate [a,b] (%)**					
		**100 µM**	**50 µM**	**25 µM**	**100 µM**	**50 µM**	**25 µM**
**a**	H	2.48±1.27	-4.75±0.54	-	26.8±4.26	19.8±0.70	10.86±2.71
**b**	4-Cl	2.54±1.87	3.93±1.07	-	48.88±2.97	39.18±1.84	24.28±3.03
**c**	3-Cl	15.33±2.84	16.64±1.67	-	27.94±4.24	26.00±0.69	13.29±0.50
**d**	2-Cl	13.82±1.29	5.32±2.00	-	33.77±4.51	24.25±2.31	11.97±1.92
**e**	4-F	5.41±1.27	-3.26±0.76	-	16.41±1.44	15.94±0.56	-
**f**	4-Br	10.29±2.38	14.52±3.50	-	34.54±2.06	26.06±1.54	17.35±2.31
**g**	4-Me	1.46±4.81	3.25±0.55	-	40.45±2.60	31.39±2.40	17.72±0.62
**h**	4-napthyl	-0.21±1.07	-8.59±1.34	-	51.79±3.16	36.95±0.57	25.59±0.30
**i**	4-NO$_2$	0.32±3.49	2.59±0.05	-	17.86±0.96	7.74±2.10	-
**g**	4-CF$_3$	-4.45±2.76	-6.28±1.07	-	26.73±3.36	26.24±1.42	19.02±1.56
**k**	3-MeO	5.67±2.48	6.15±0.33	-	14.58±2.52	14.64±3.12	-
**l**	4-MeO	4.74±2.25	5.48±0.32	-	14.72±1.58	20.99±2.19	-

[a] All data represent mean ± S.D. from different experiments performed in triplicate.

[b] “-” means no test.

Table 2 Biofilm inhibition rates of derivatives against the *P. aeruginosa* PAO1.

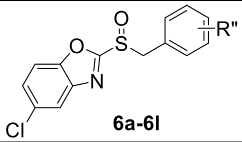

6a-6l

Entry	Compd.	Inhibition rate [a,b] (%)		
		**100 µM**	**50 µM**	**25 µM**
**a**	H	18.07±1.71	12.84±4.17	-
**b**	4-Cl	52.78±2.89	46.13±0.72	33.09±2.99
**c**	3-Cl	12.35±1.60	13.77±0.27	-
**d**	2-Cl	14.48±1.85	15.14±2.25	-
**e**	4-F	41.98±2.26	38.64±0.32	21.10±3.77
**f**	4-Br	10.92±1.29	14.6±1.23	-
**g**	4-Me	18.79±4.20	16.27±1.68	-
**h**	4-napthyl	14.36±0.24	9.95±0.59	-
**i**	4-NO_2	24.28±1.38	16.45±1.69	-
**g**	4-CF_3	19.16±2.19	15.65±0.84	-
**k**	3-MeO	18.30±1.10	15.85±3.33	-
**l**	4-MeO	17.94±1.28	12.51±1.41	-

[a] All data represent mean ± S.D. from different experiments performed in triplicate.

[b] "-" means no test.

Table 3 Biofilm inhibition rates of derivatives against the *P. aeruginosa* PAO1.

Entry	Compd.	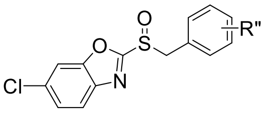 7a-7f			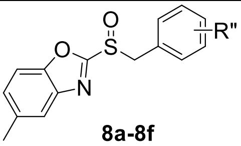 8a-8f		
		Inhibition rate [a,b] (%)					
		100 µM	50 µM	25 µM	100 µM	50 µM	25 µM
**a**	H	22.56±3.02	19.11±2.12	11.49±2.24	16.98±3.74	11.92±0.58	-
**b**	4-Cl	55.84±1.78	43.64±2.49	27.7±1.37	16.47±2.74	19.93±0.57	9.5±2.96
**e**	4-F	28.69±1.20	23.75±0.52	14.75±2.19	6.53±3.55	9.52±1.7	-
**g**	4-Me	23.92±1.46	22.30±3.69	13.53±2.28	8.29±2.60	12.38±2.54	-
**i**	4-NO_2	31.40±1.63	26.45±1.21	15.79±2.83	19.94±3.56	22.07±0.82	12.01±1.98
**l**	4-MeO	23.59±3.34	19.09±1.16	11.33±0.72	10.50±0.52	14.19±1.83	-

[a] All data represent mean ± S.D. from different experiments performed in triplicate.

[b] “-” means no test.

Table 4 Biofilm inhibition rates of derivatives against the *P. aeruginosa* PAO1.

Entry	Compd.	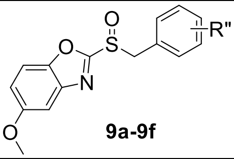 9a-9f		
		Inhibition rate [a,b] (%)		
		100 µM	50 µM	25 µM
**a**	H	16.2±3.03	13.44±0.37	–
**b**	4-Cl	33.18±3.39	29.44±0.39	14.92±2.31
**e**	4-F	7.90±0.21	13.33±0.96	–
**g**	4-Me	14.75±2.34	13.18±0.69	–
**i**	4-NO_2	21.01±1.58	18.75±0.52	–
**l**	4-MeO	34.07±2.78	28.56±0.45	16.51±1.67

[a] All data represent mean ± S.D. from different experiments performed in triplicate.

[b] “-” means no test.

Cytotoxicity of **6b**

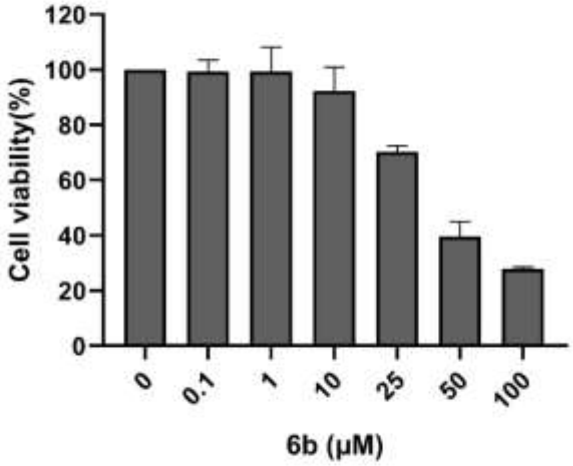

Figure S-1. RAW 264.7 cells were treated with 6b at various concentrations (100, 50, 25, 10, 1, 0.1, 0 mM) for 24 h and cell viability was determined using CCK-8.

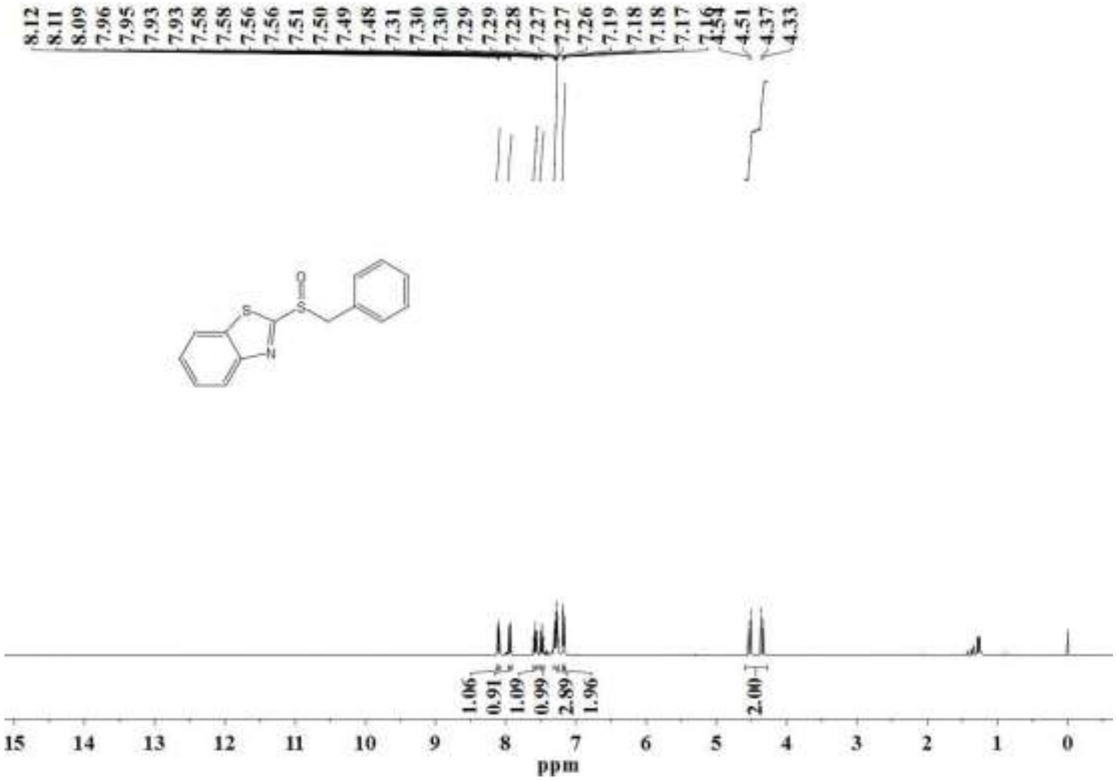

Figure S 1-1. ^{1}H NMR spectrum of compound **4a**.

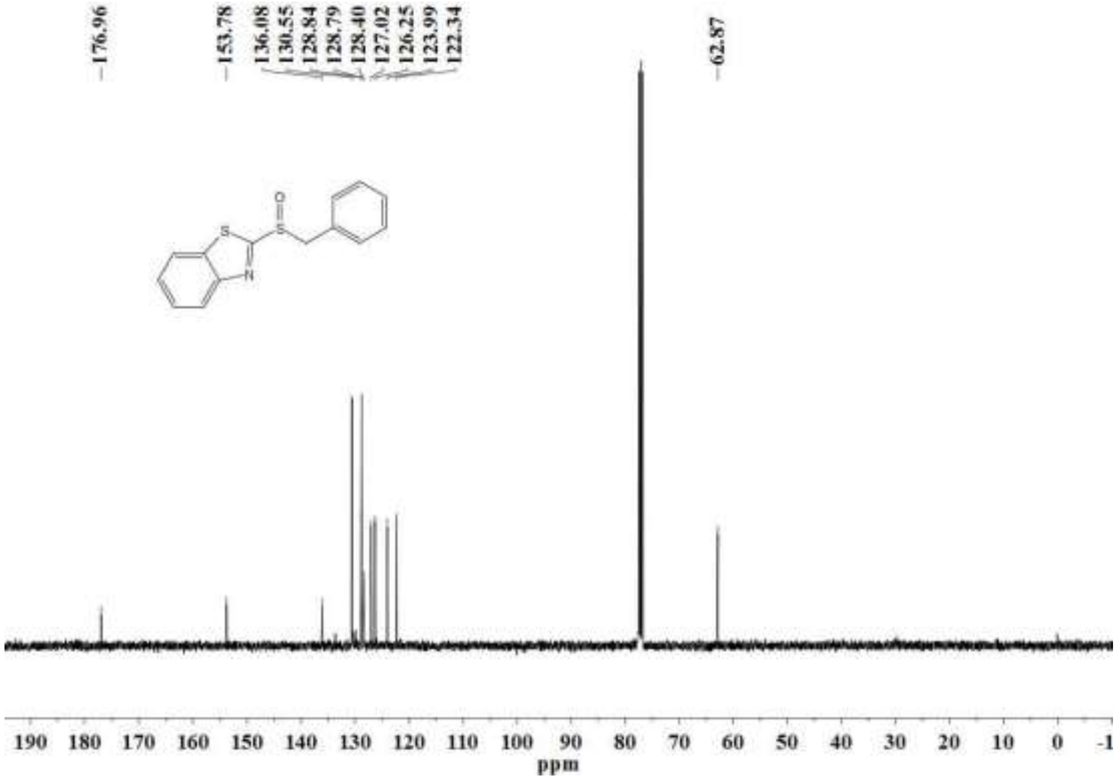

Figure S 1-2. ^{13}C NMR spectrum of compound **4a**.

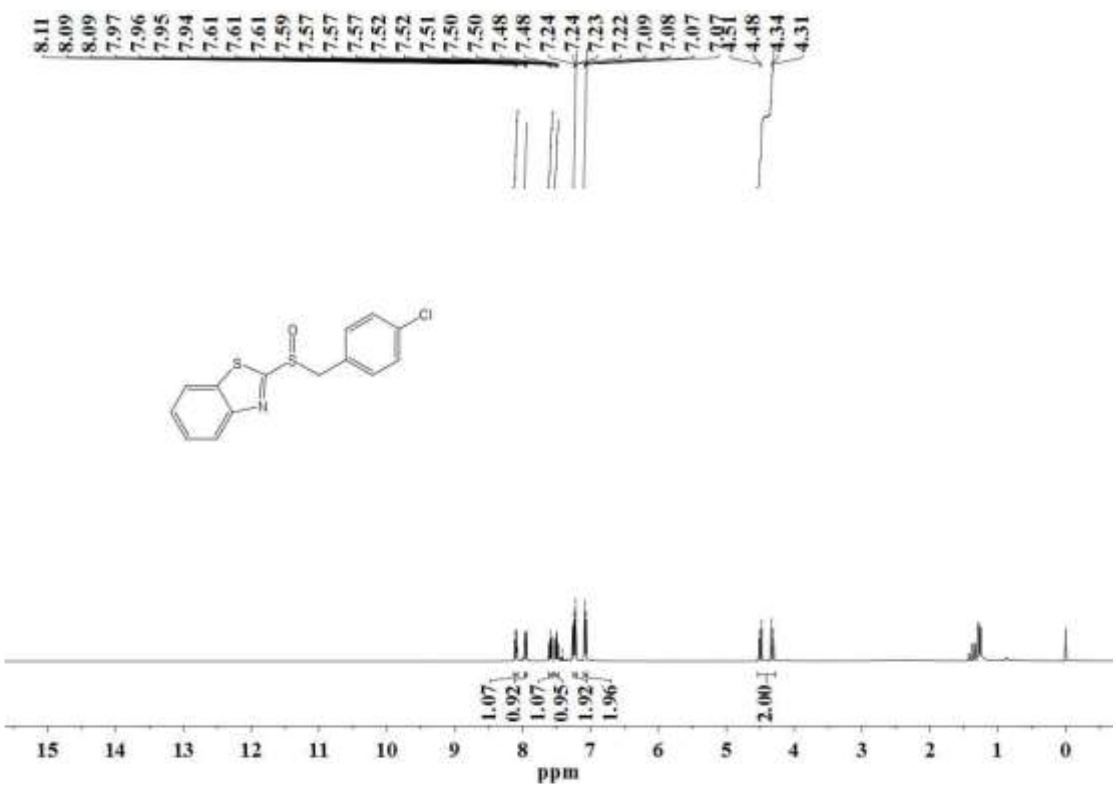

Figure S 2-1. ^{1}H NMR spectrum of compound **4b**.

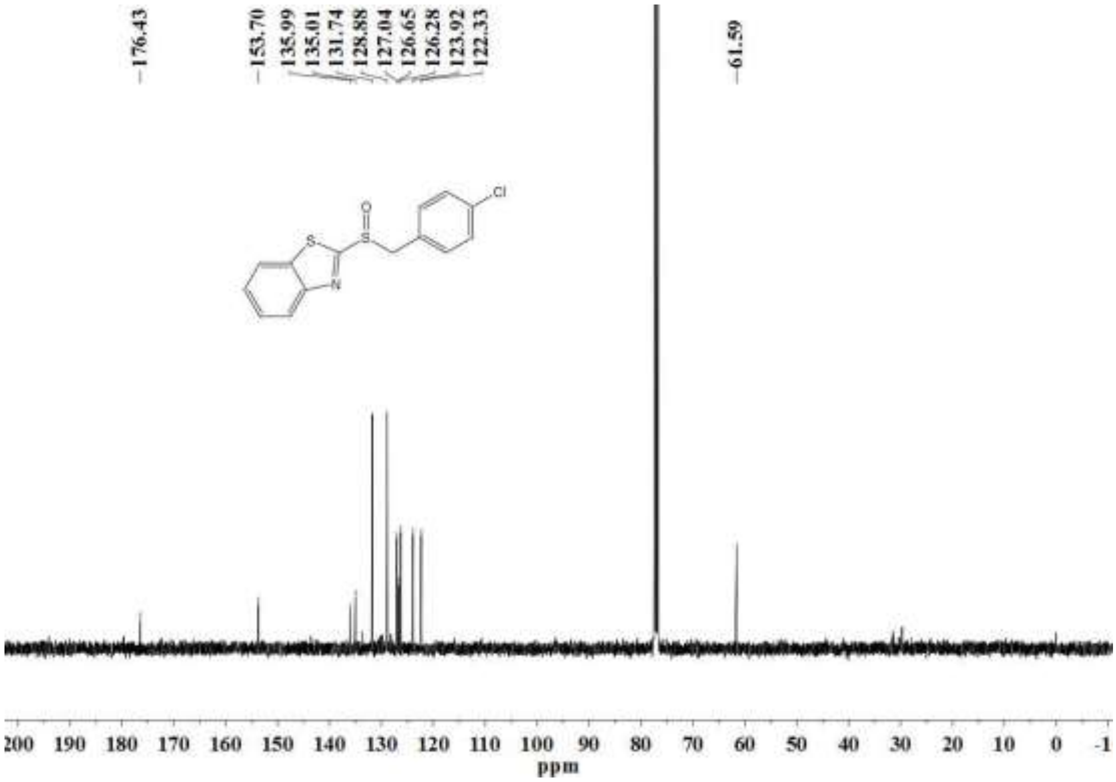

Figure S 2-2. ^{13}C NMR spectrum of compound **4b**.

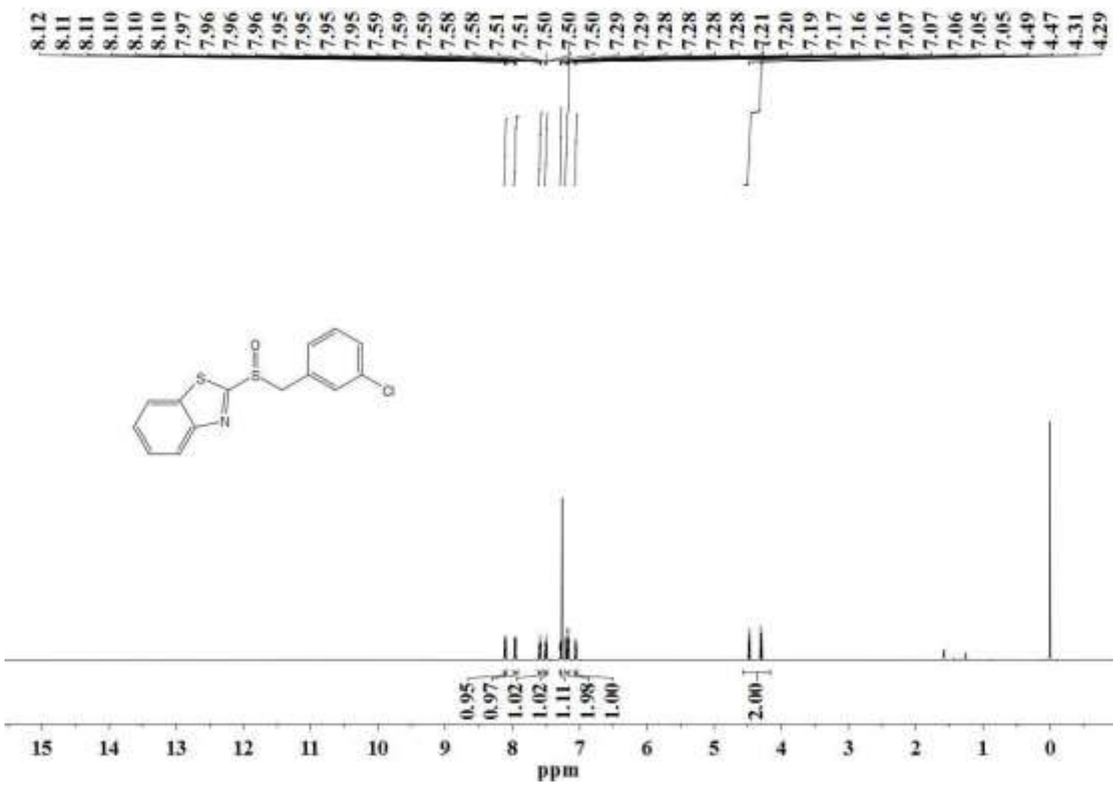

Figure S 3-1. ^{1}H NMR spectrum of compound **4c**.

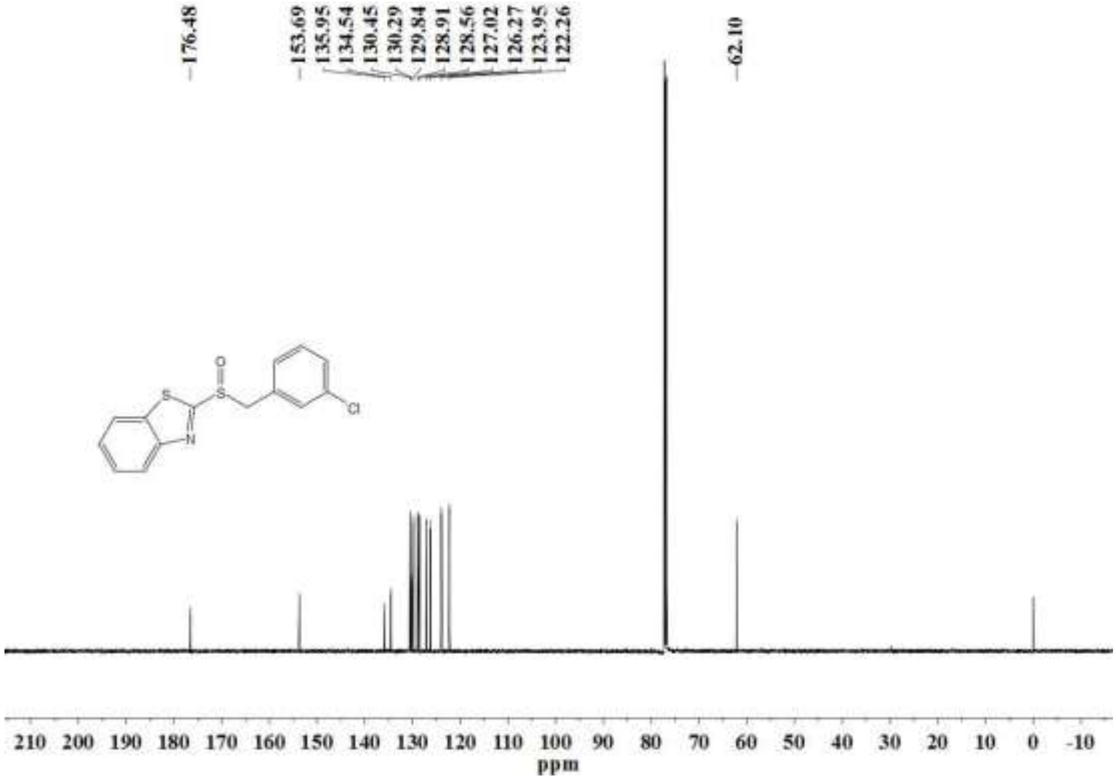

Figure S 3-2. ^{13}C NMR spectrum of compound **4c**.

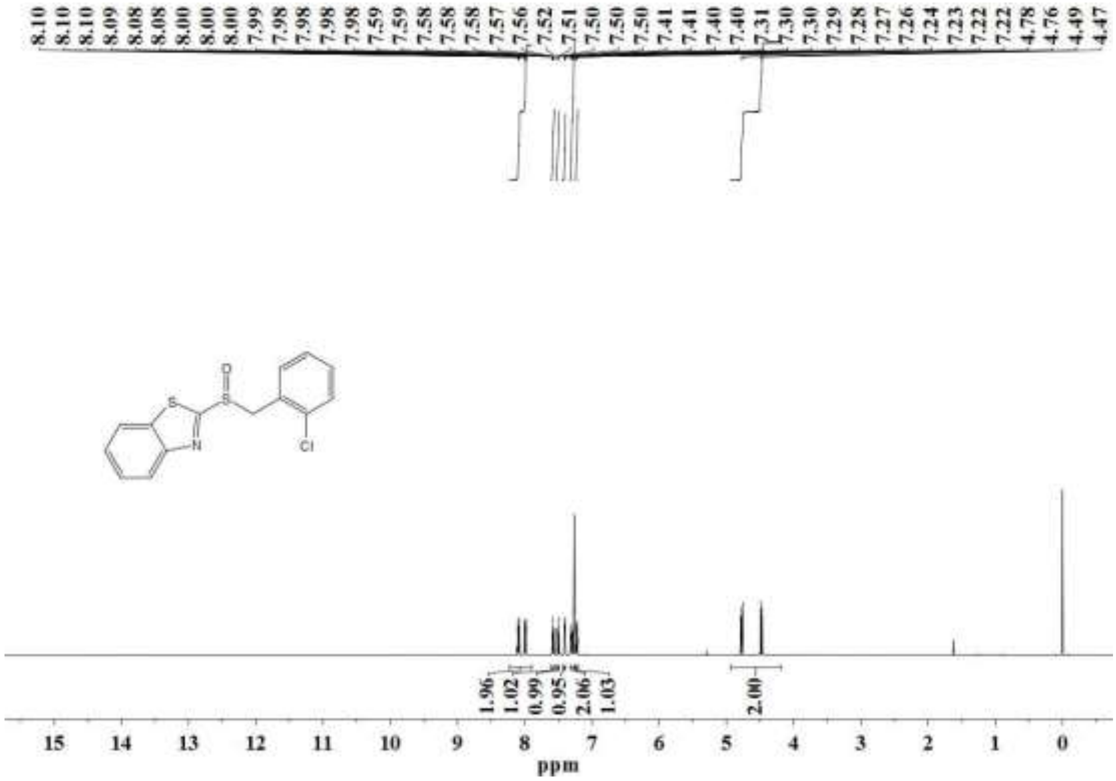

Figure S 4-1. ^{1}H NMR spectrum of compound **4d**.

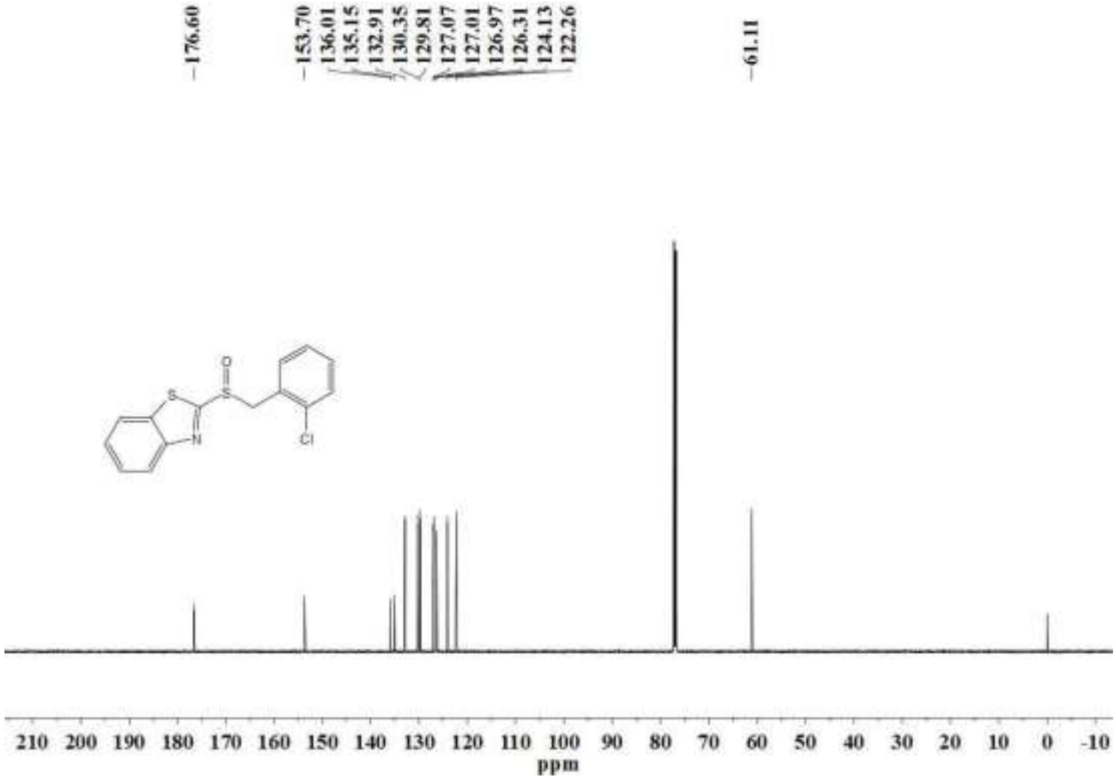

Figure S 4-2. ^{13}C NMR spectrum of compound **4d**.

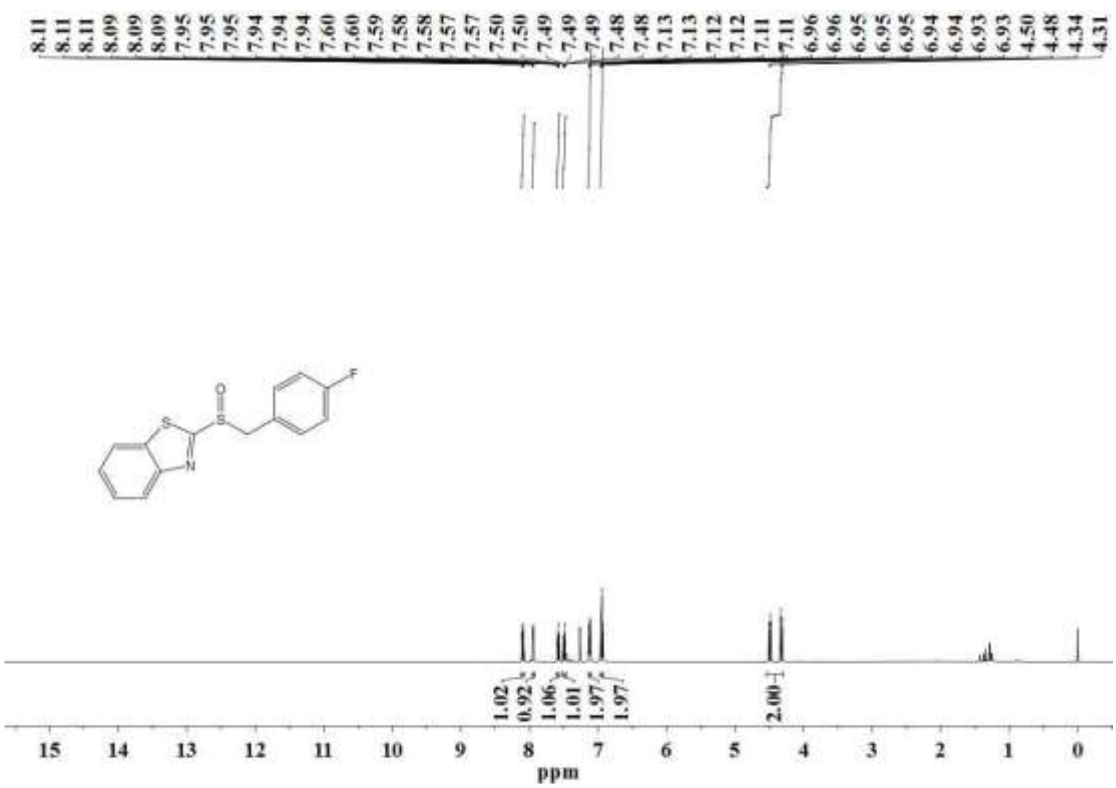

Figure S 5-1. ^{1}H NMR spectrum of compound **4e**.

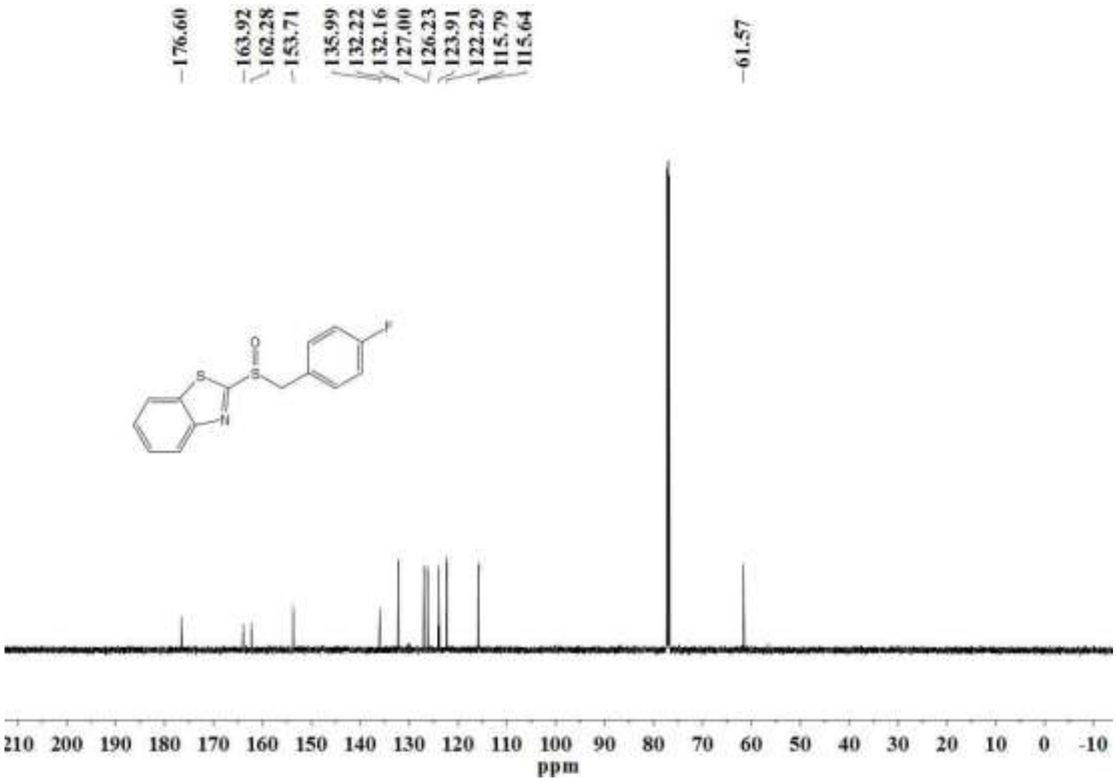

Figure S 5-2. ^{13}C NMR spectrum of compound **4e**.

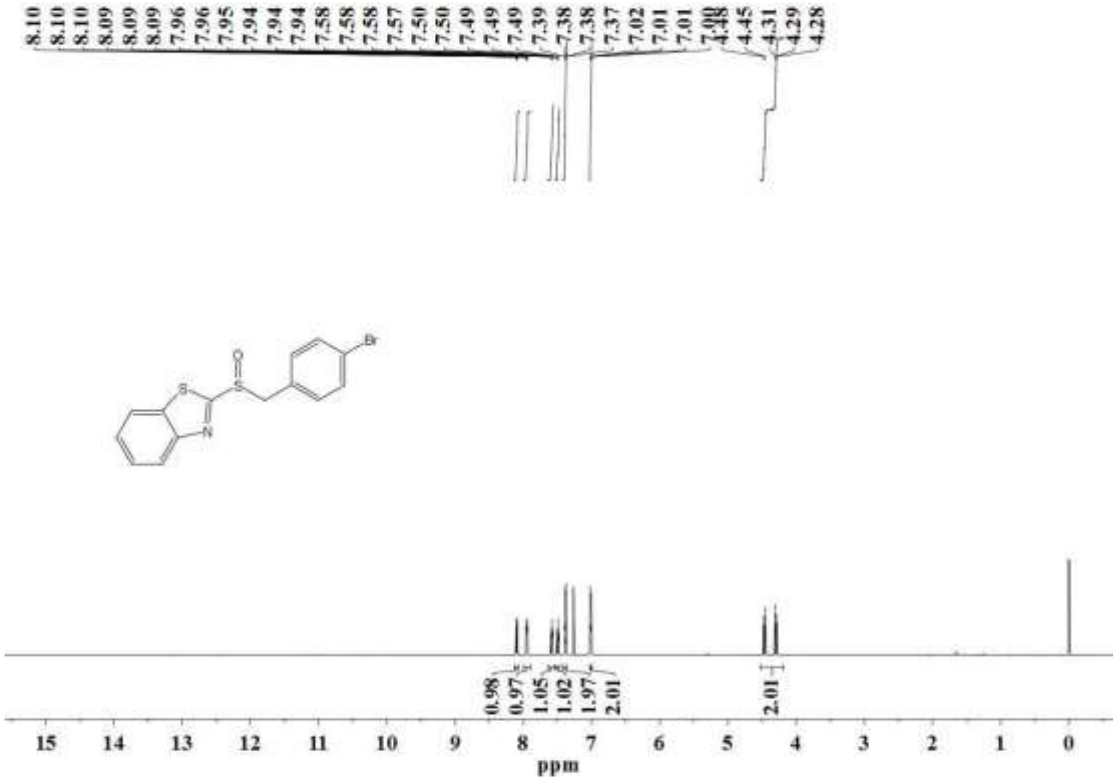

Figure S 6-1. ^{1}H NMR spectrum of compound **4f**.

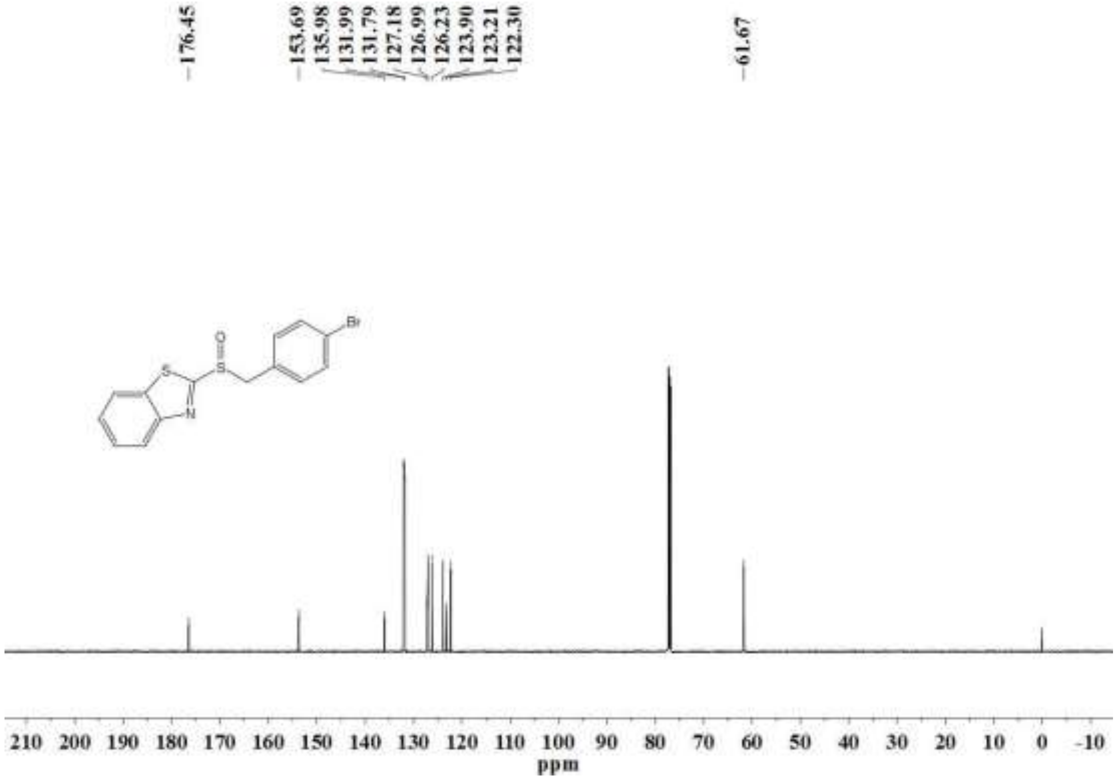

Figure S 6-2. ^{13}C NMR spectrum of compound **4f**.

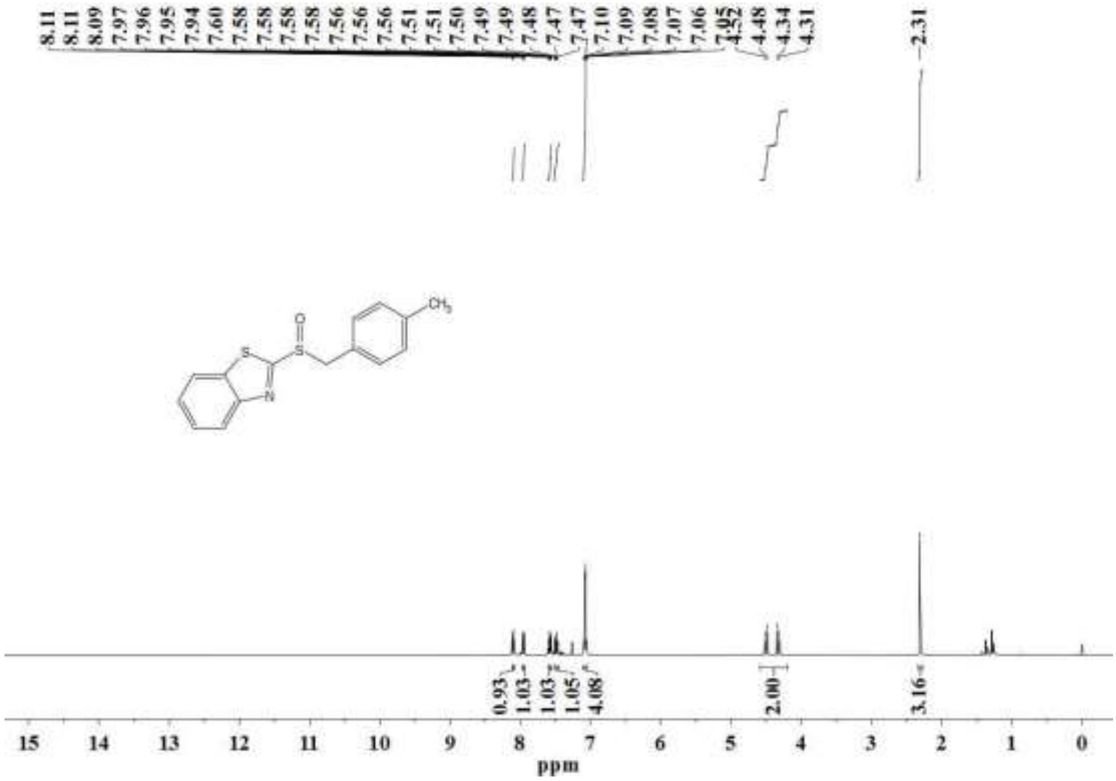

Figure S 7-1. ^{1}H NMR spectrum of compound **4g**.

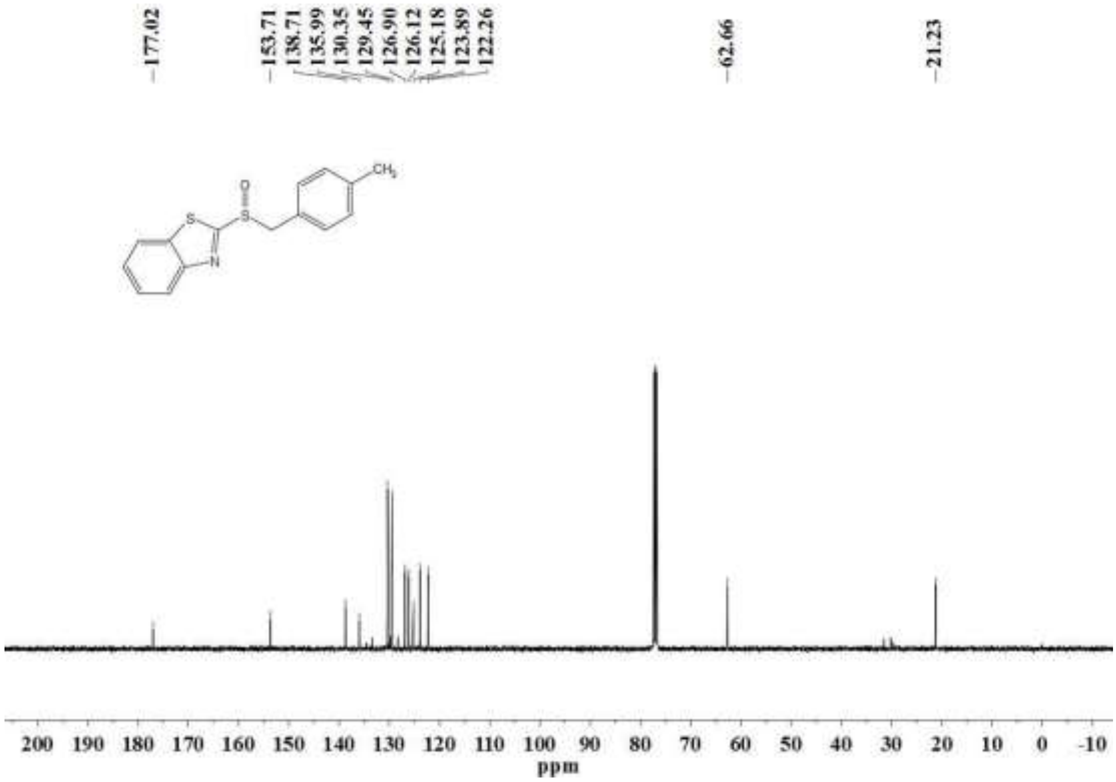

Figure S 7-2. ^{13}C NMR spectrum of compound **4g**.

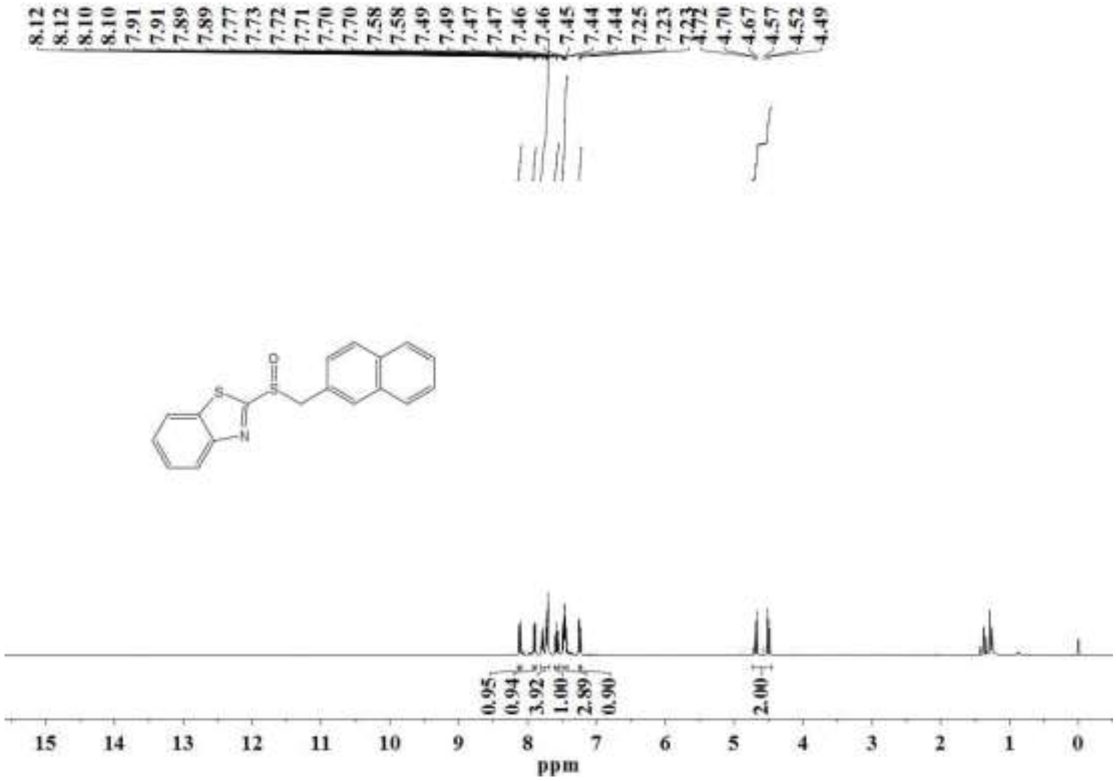

Figure S 8-1. ^{1}H NMR spectrum of compound **4h**.

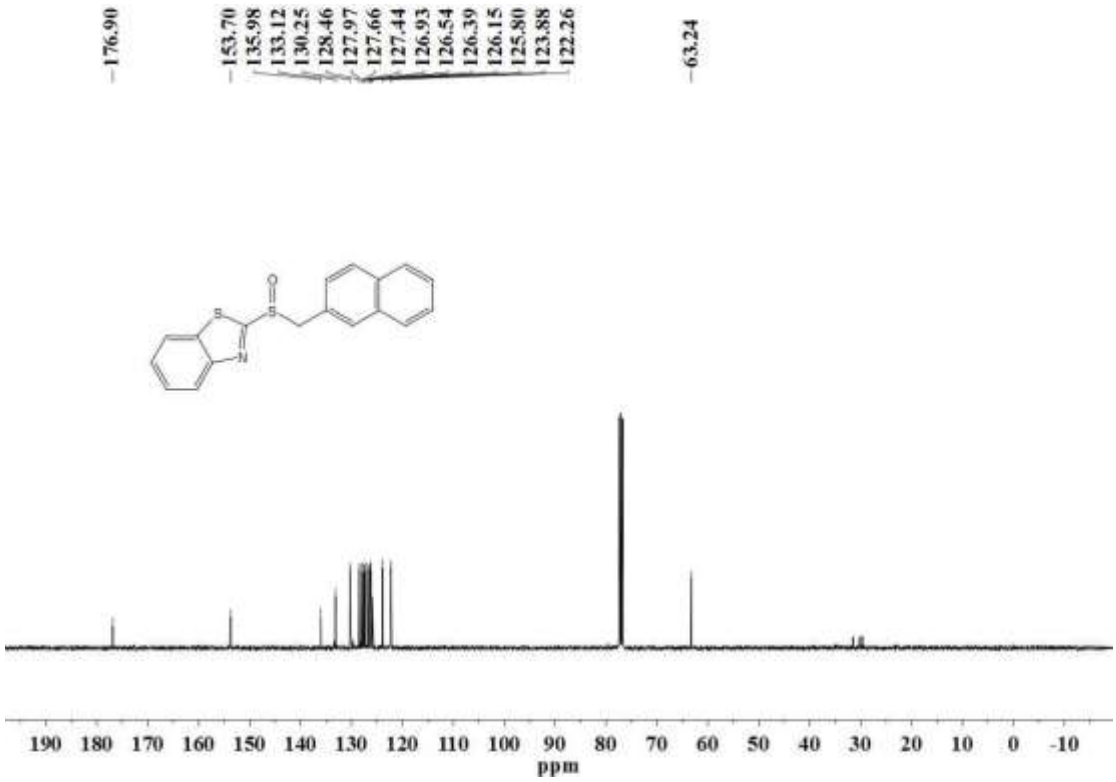

Figure S 8-2. ^{13}C NMR spectrum of compound **4h**.

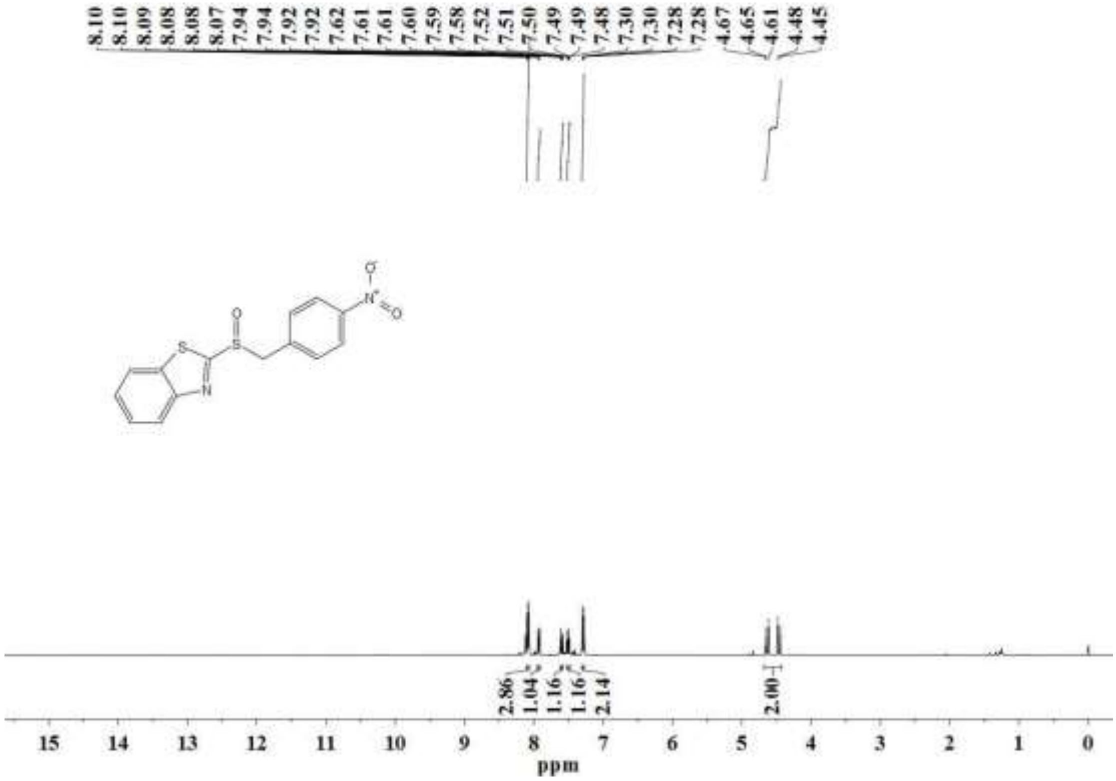

Figure S 9-1. ^{1}H NMR spectrum of compound **4i**.

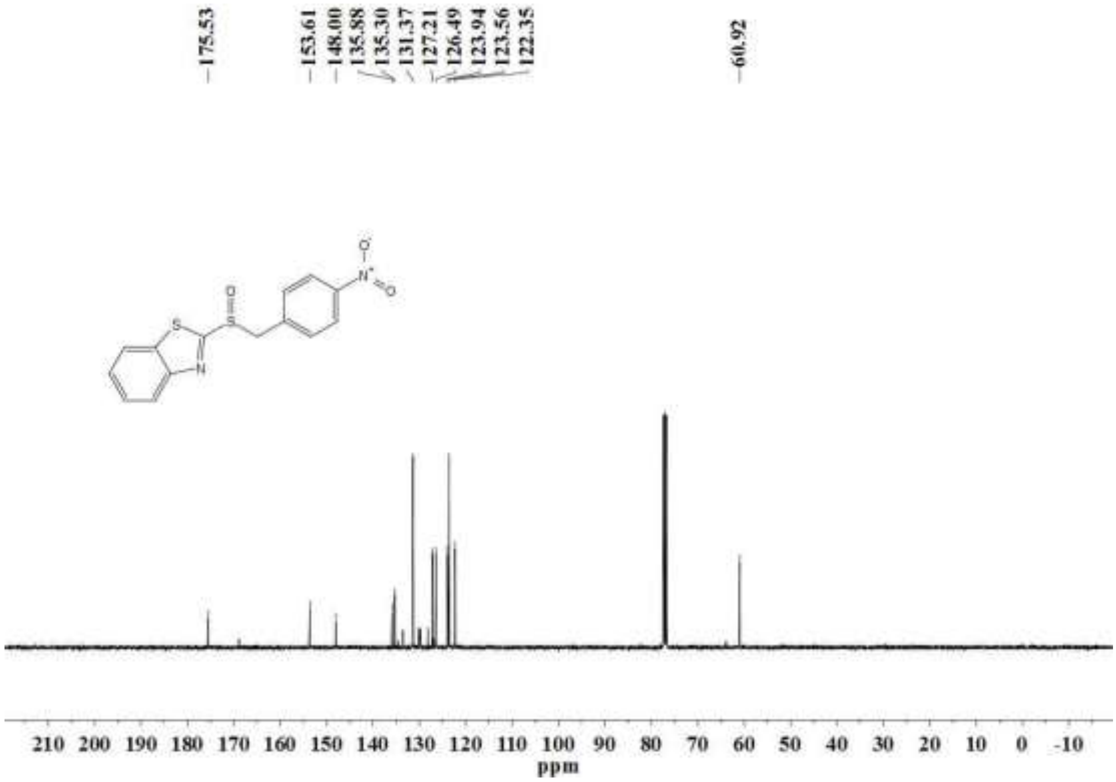

Figure S 9-2. ^{13}C NMR spectrum of compound **4i**.

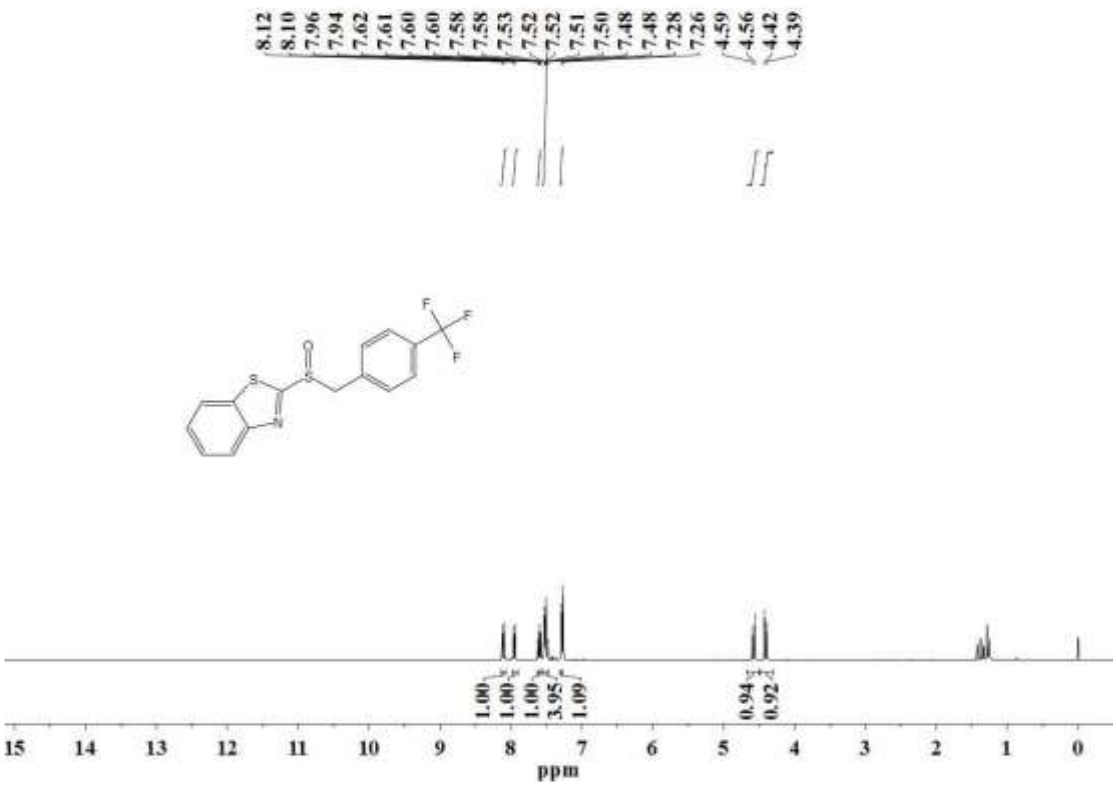

Figure S 10-1. ^{1}H NMR spectrum of compound **4j**.

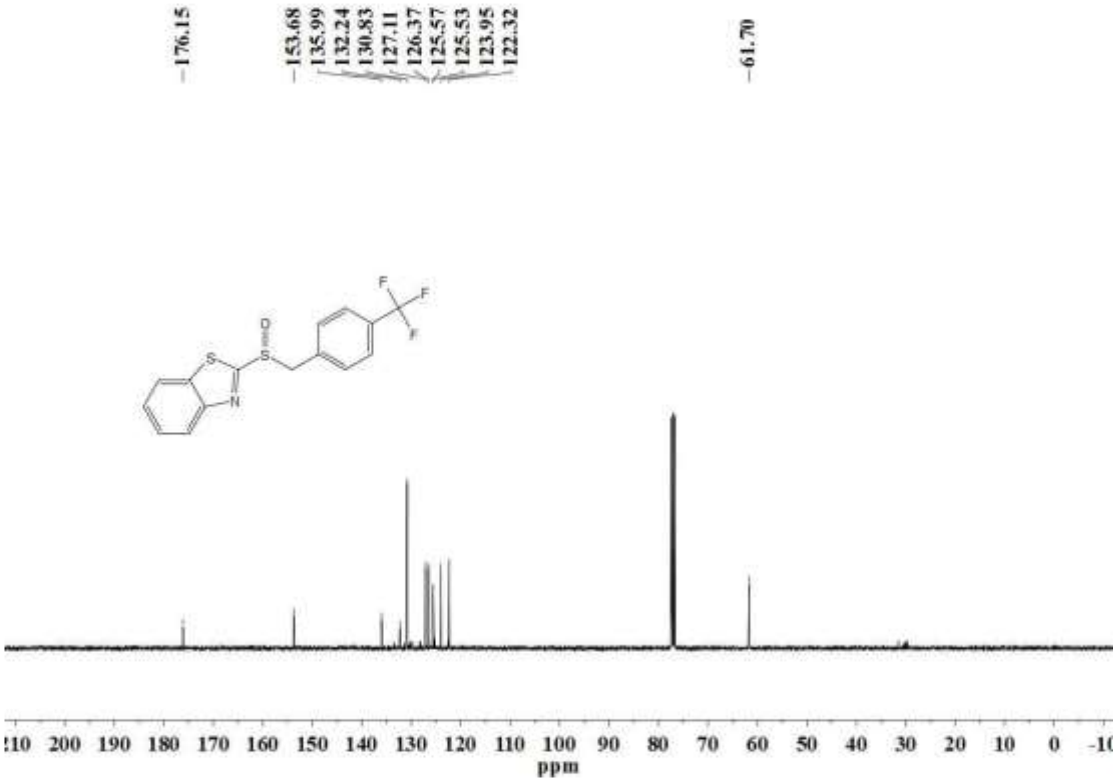

Figure S 10-2. ^{13}C NMR spectrum of compound **4j**.

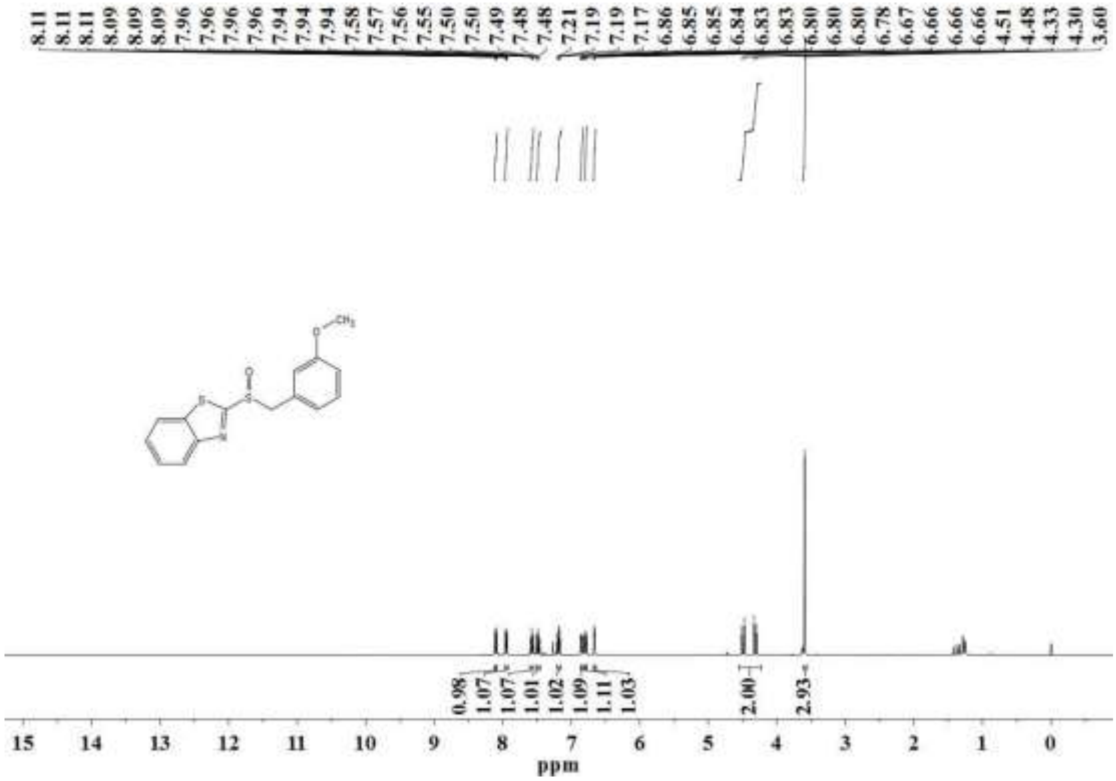

Figure S 11-1. ^{1}H NMR spectrum of compound **4k**.

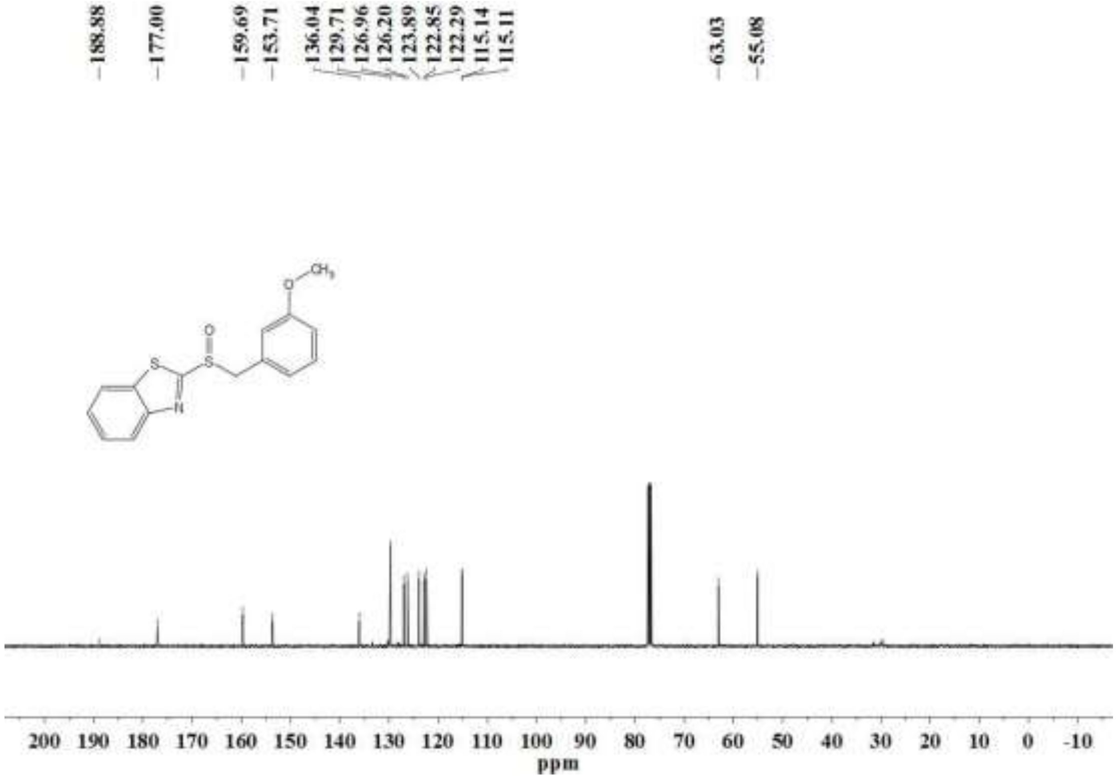

Figure S 11-2. ^{13}C NMR spectrum of compound **4k**.

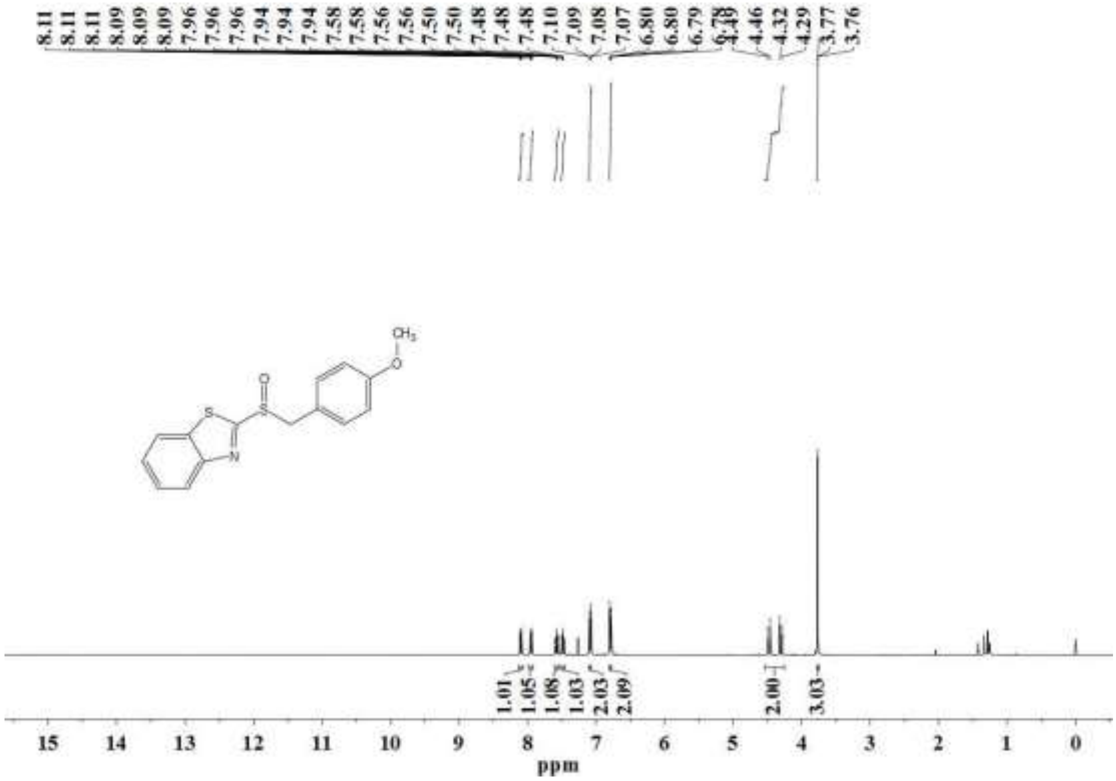

Figure S 12-1. ^{1}H NMR spectrum of compound **4l**.

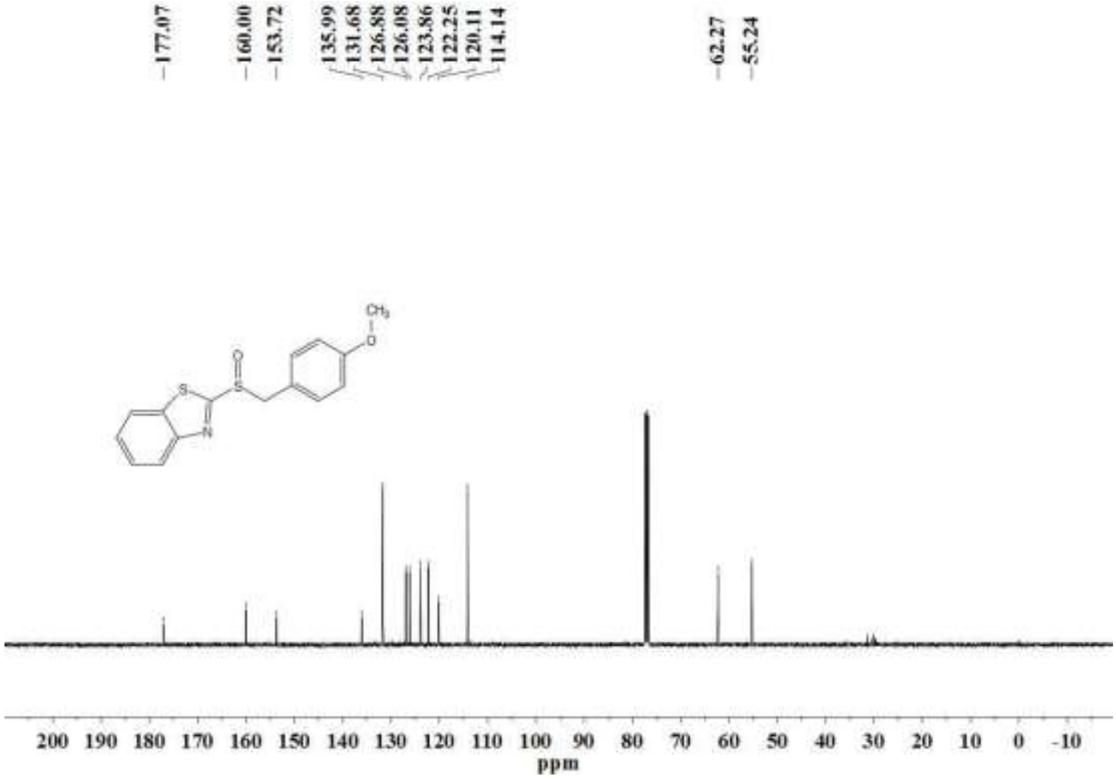

Figure S 12-2. ^{13}C NMR spectrum of compound **4l**.

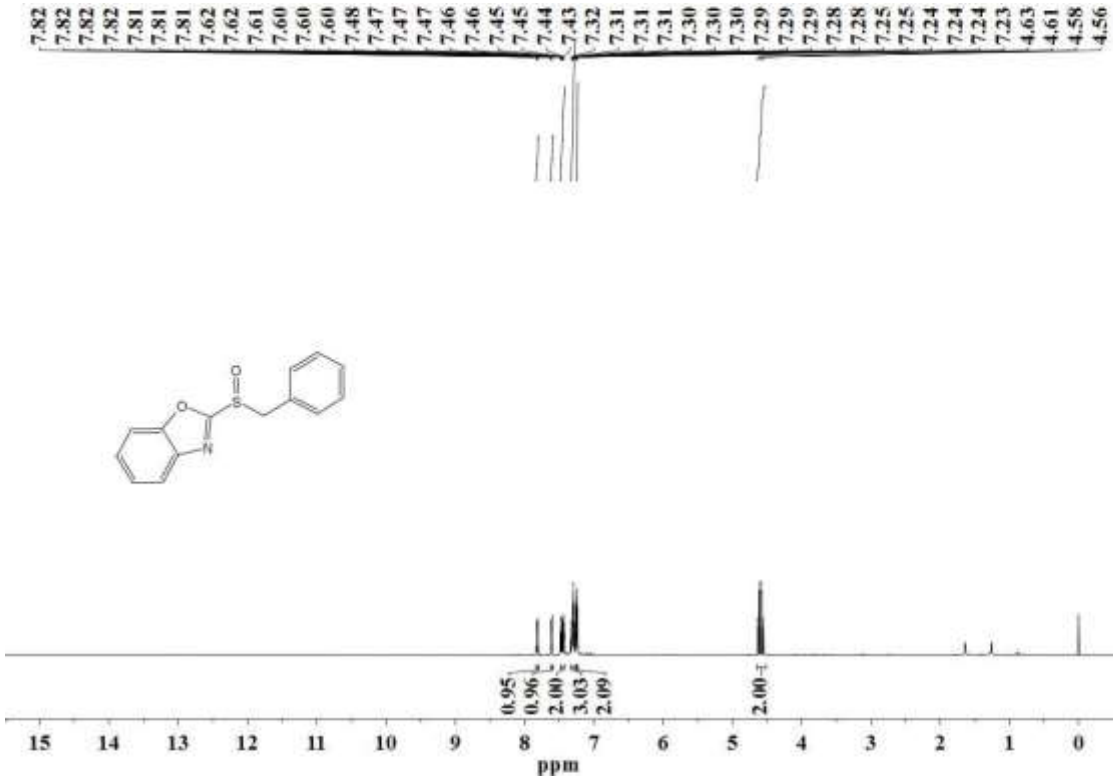

Figure S 13-1. ^{1}H NMR spectrum of compound **5a**.

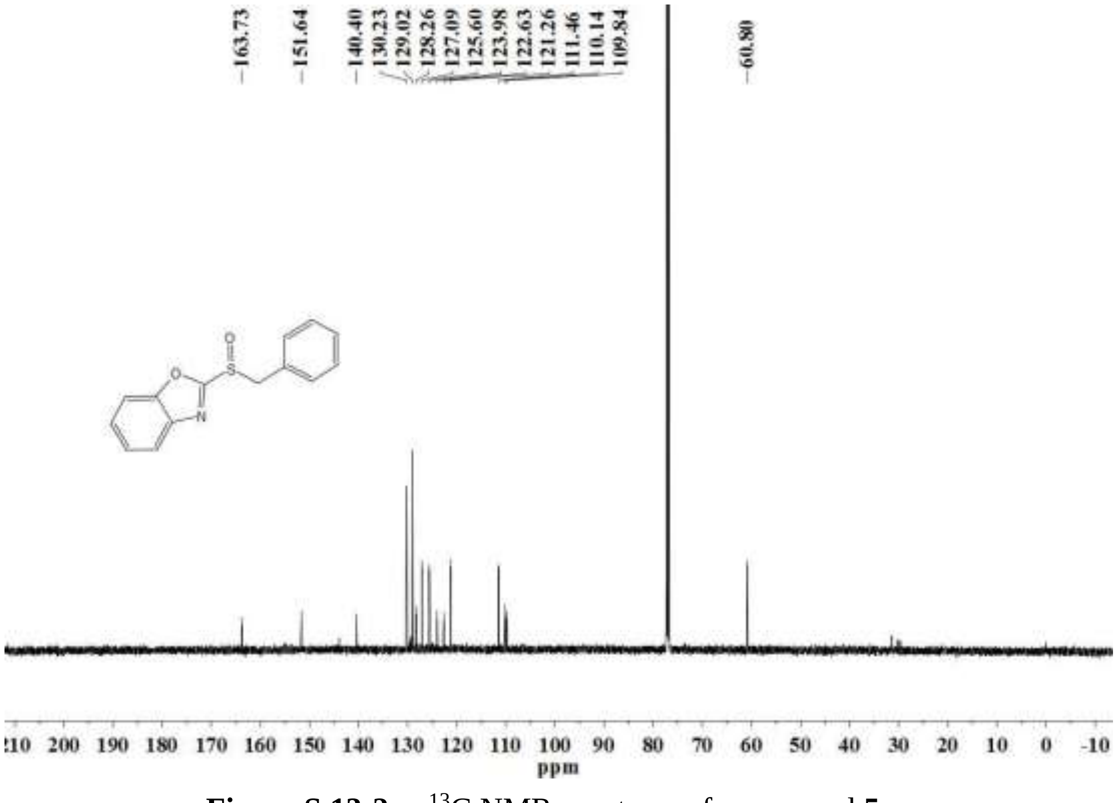

Figure S 13-2. ^{13}C NMR spectrum of compound **5a**.

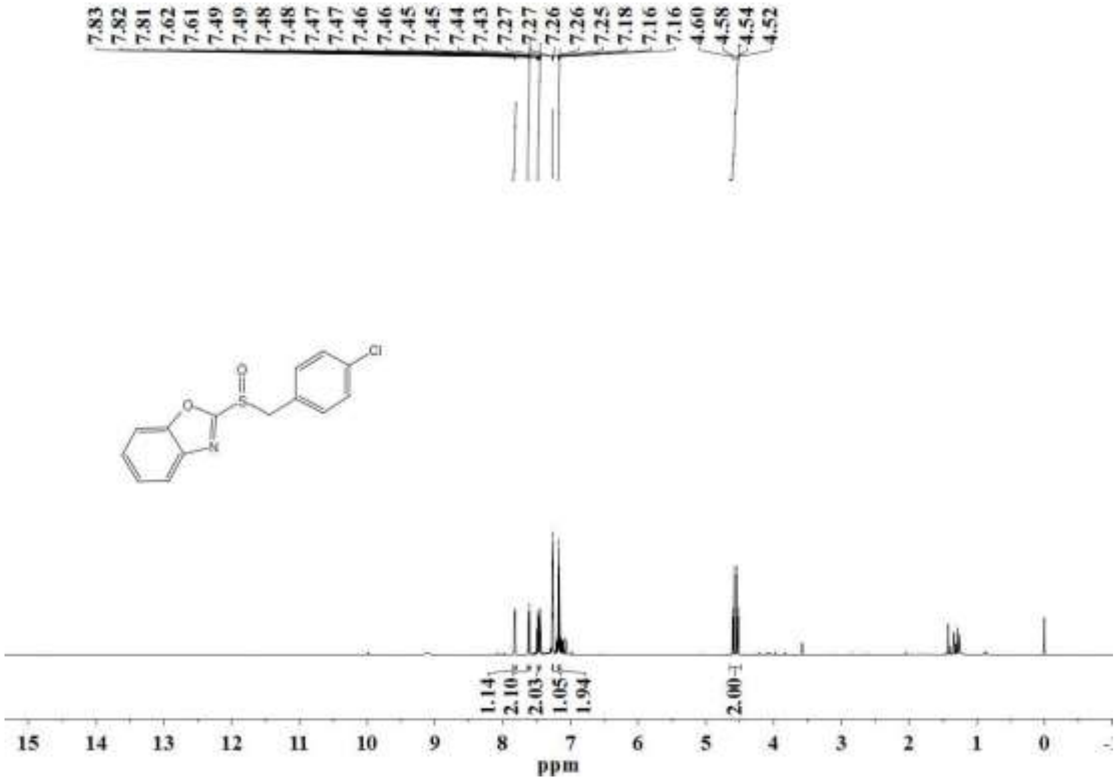

Figure S 14-1. ^{1}H NMR spectrum of compound **5b**.

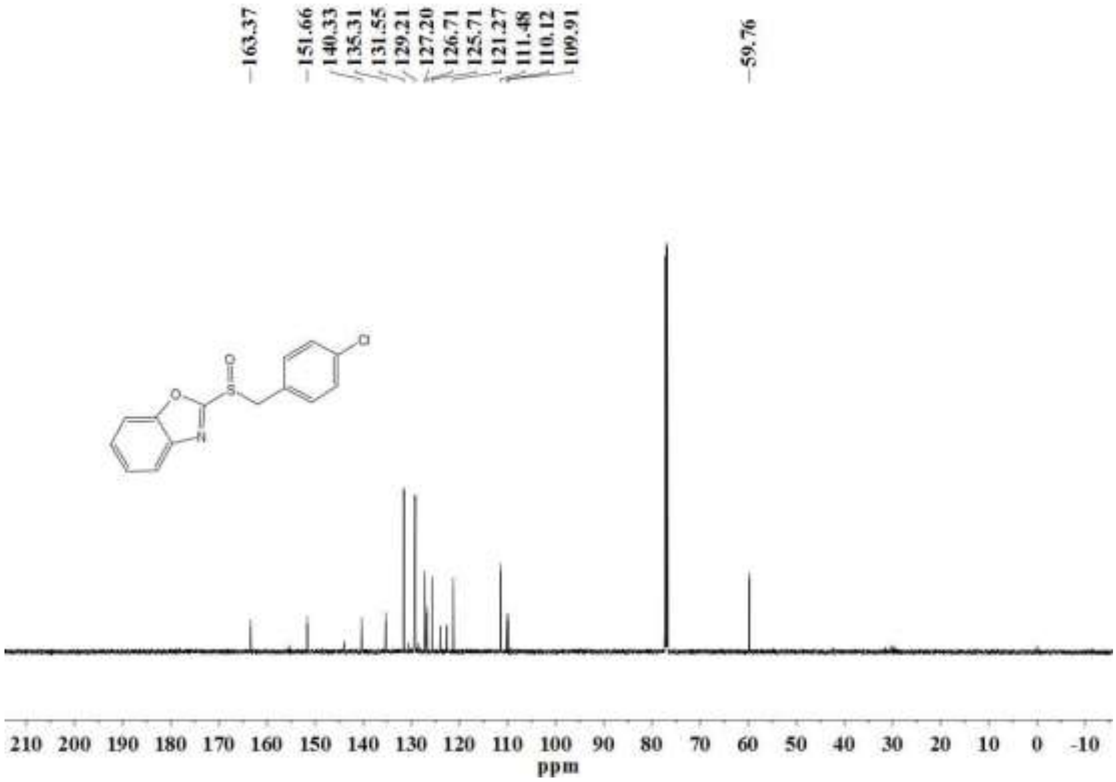

Figure S 14-2. ^{13}C NMR spectrum of compound **5b**.

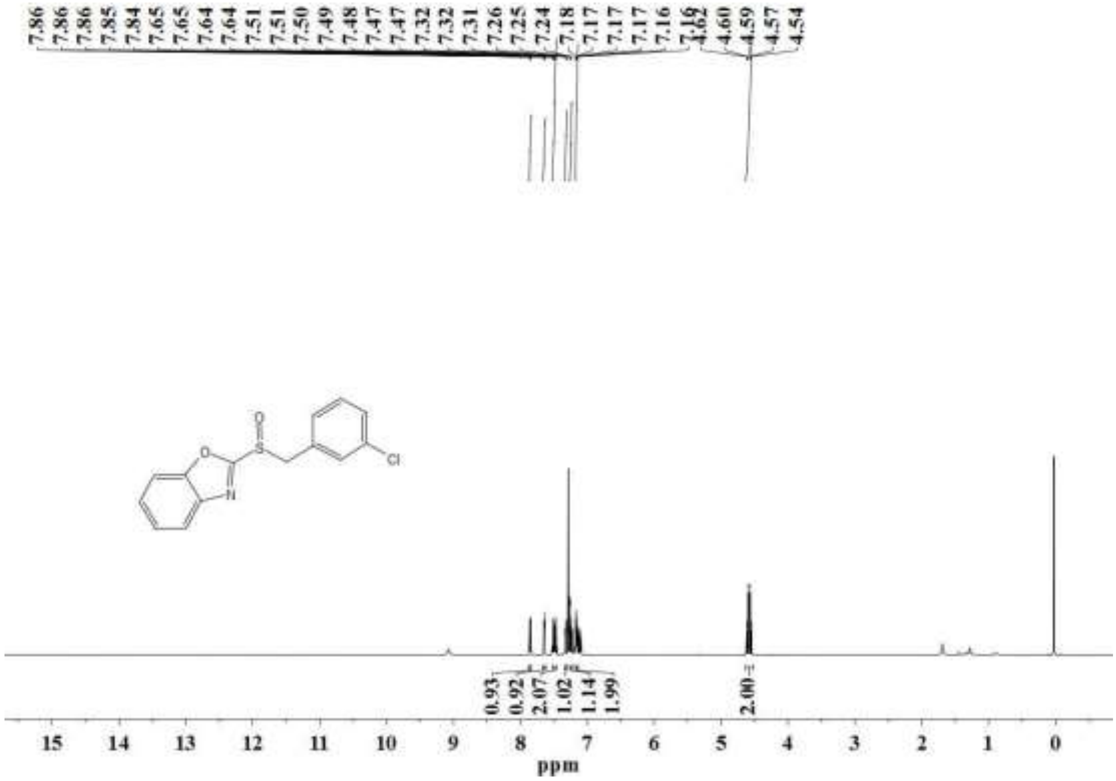

Figure S 15-1. ^{1}H NMR spectrum of compound **5c**.

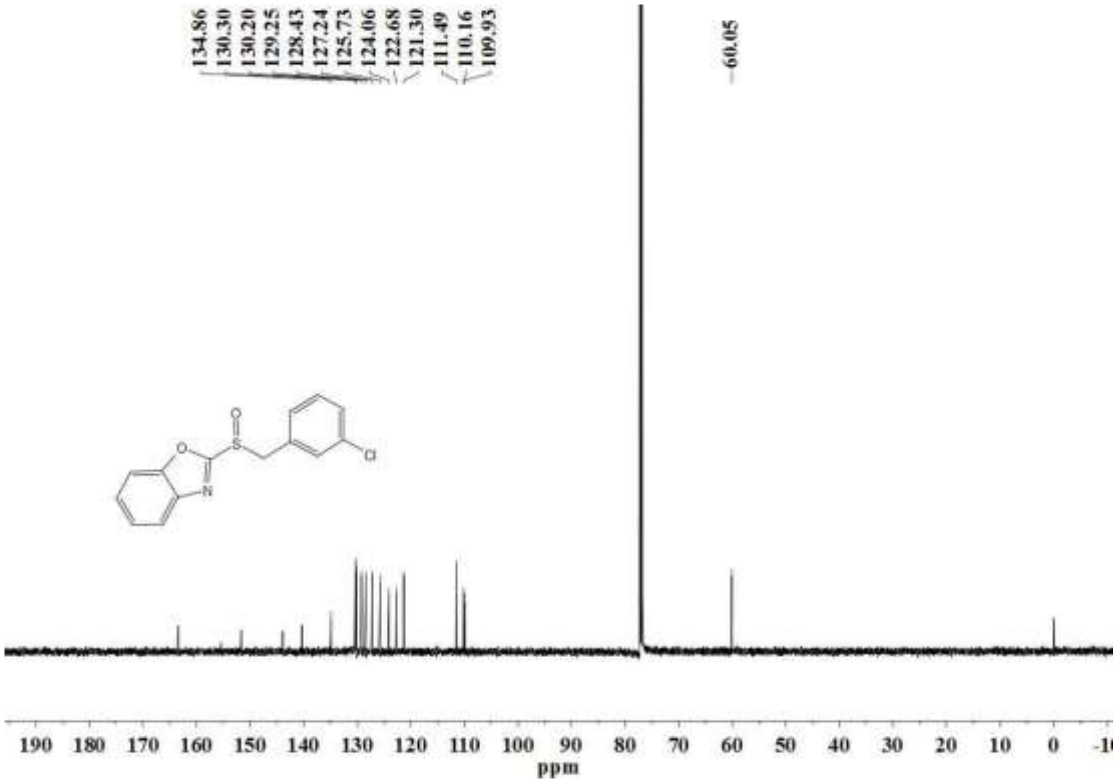

Figure S 15-2. ^{13}C NMR spectrum of compound **5c**.

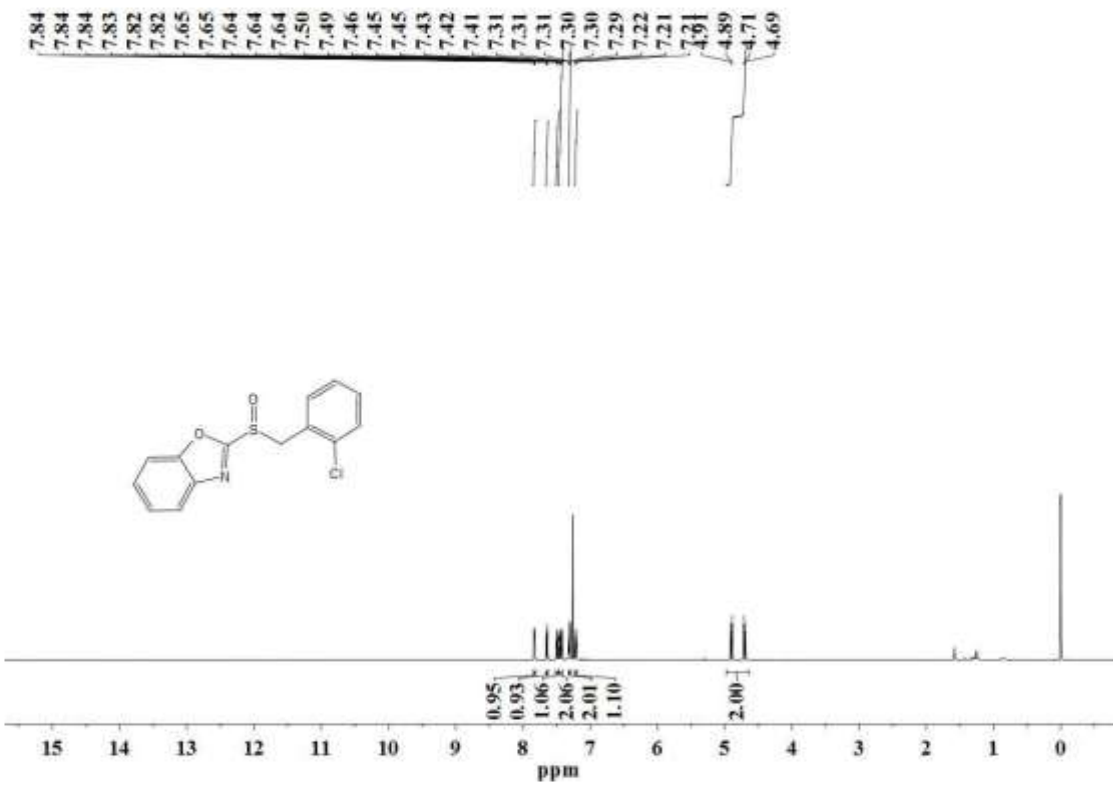

Figure S 16-1. ^{1}H NMR spectrum of compound **5d**.

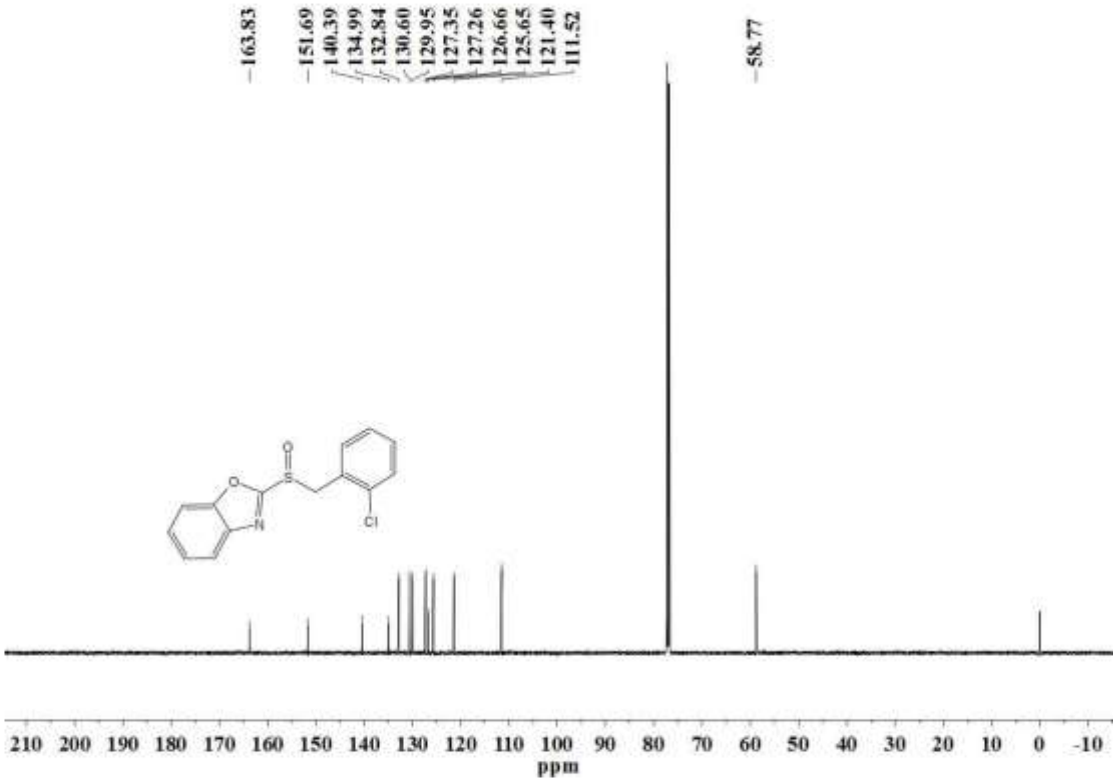

Figure S 16-2. ^{13}C NMR spectrum of compound **5d**.

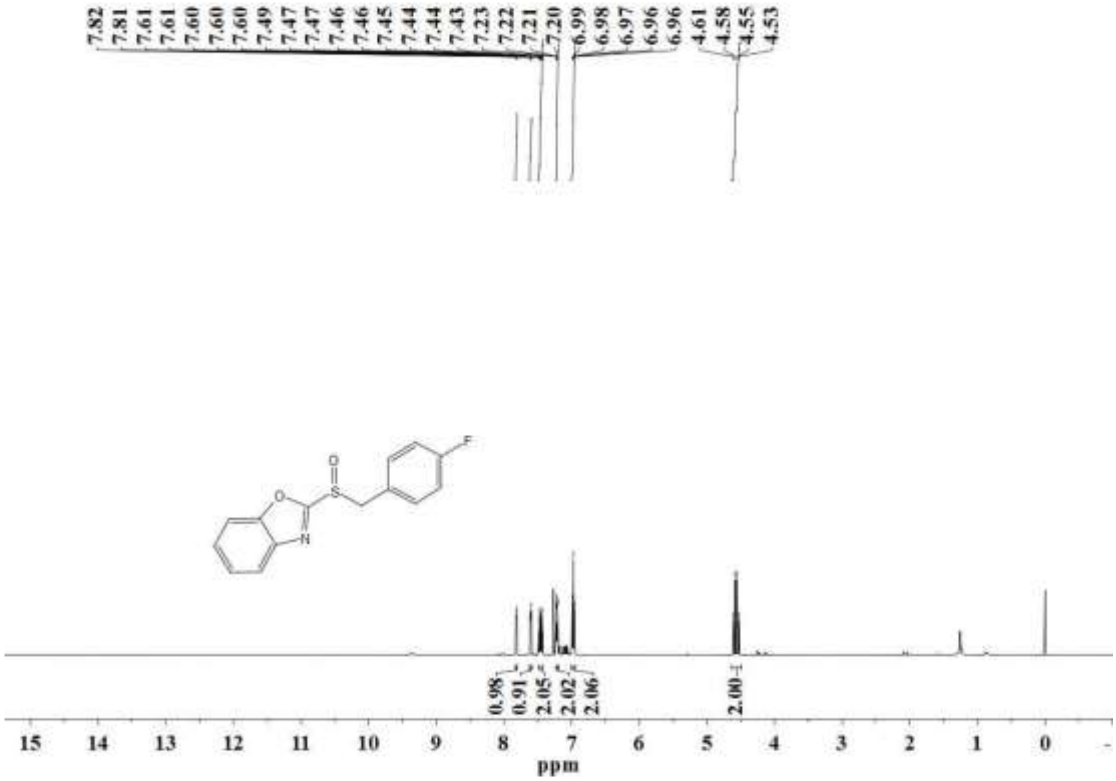

Figure S 17-1. ^{1}H NMR spectrum of compound **5e**.

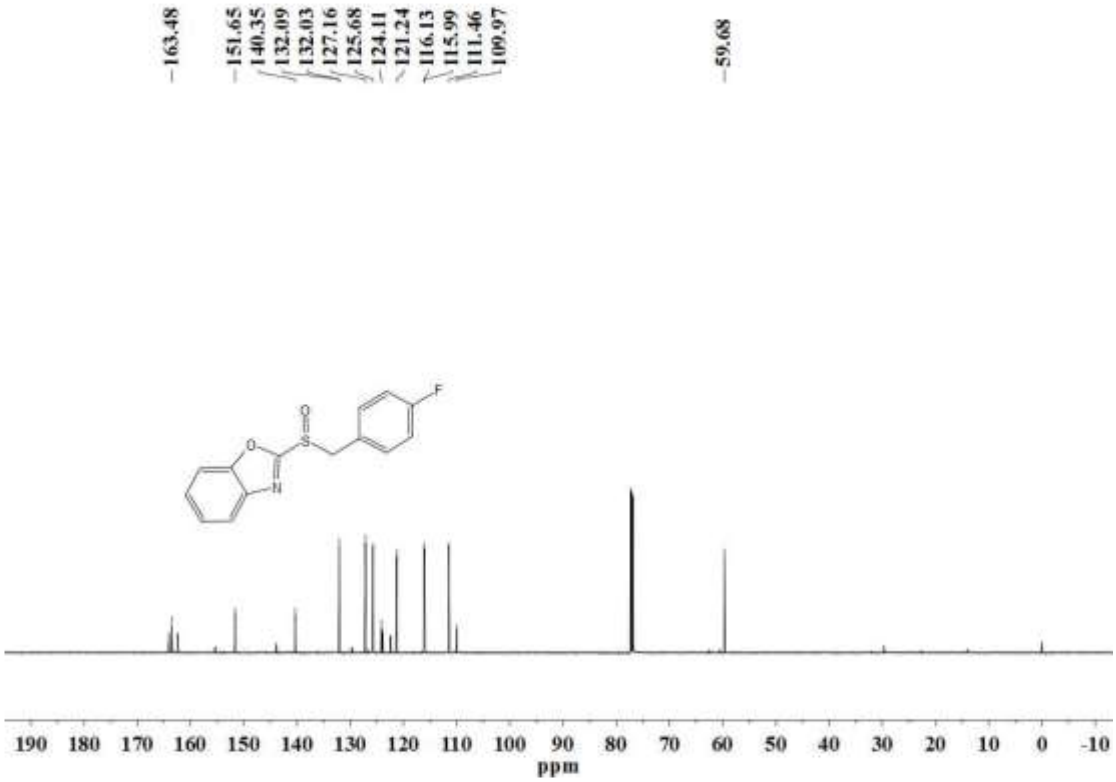

Figure S 17-2. ^{13}C NMR spectrum of compound **5e**.

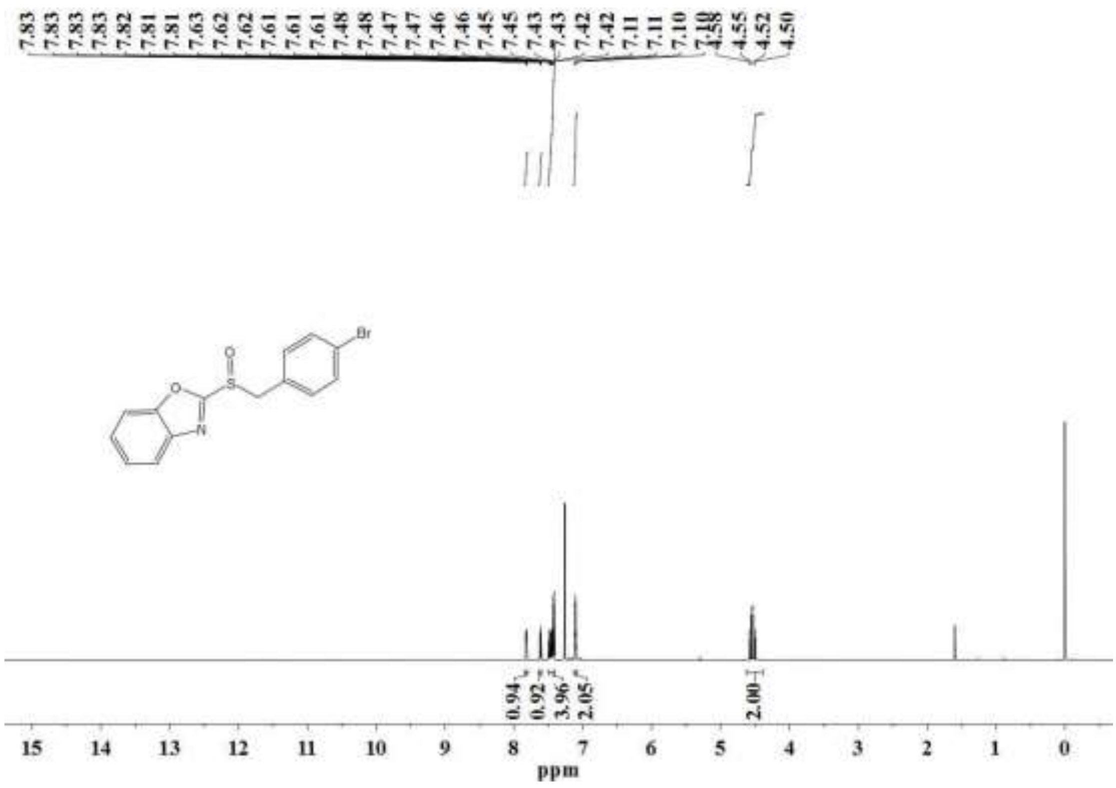

Figure S 18-1. ^{1}H NMR spectrum of compound **5f**.

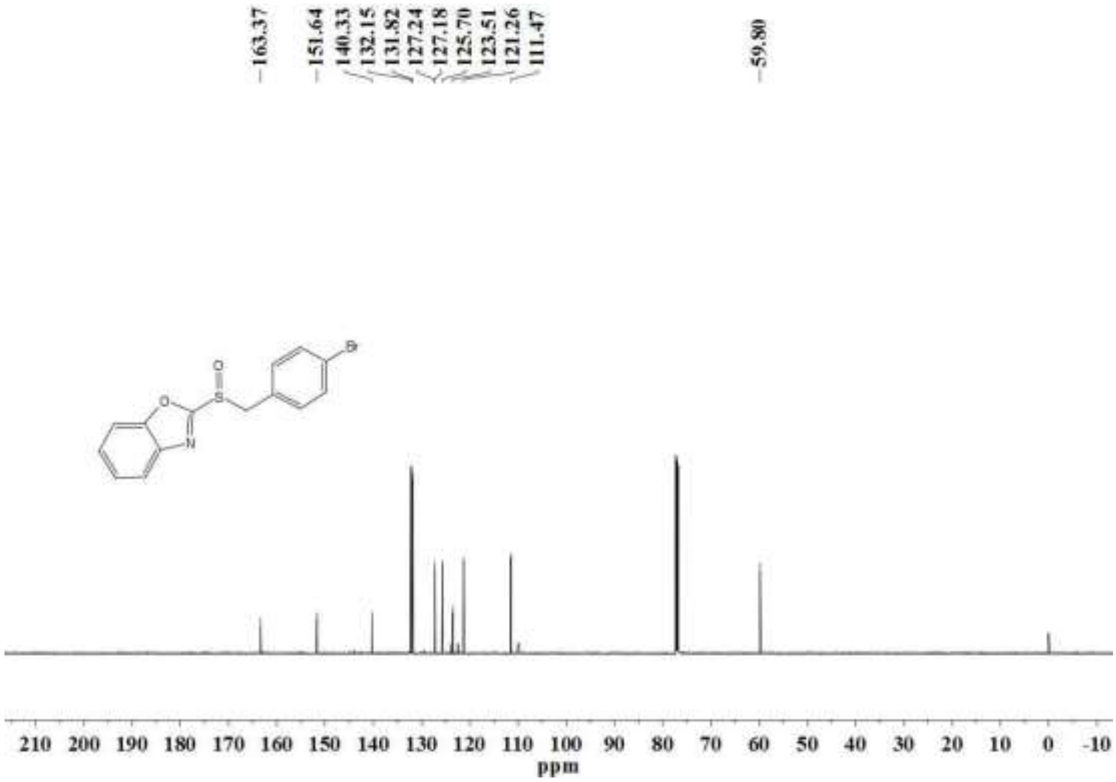

Figure S 18-2. ^{13}C NMR spectrum of compound **5f**.

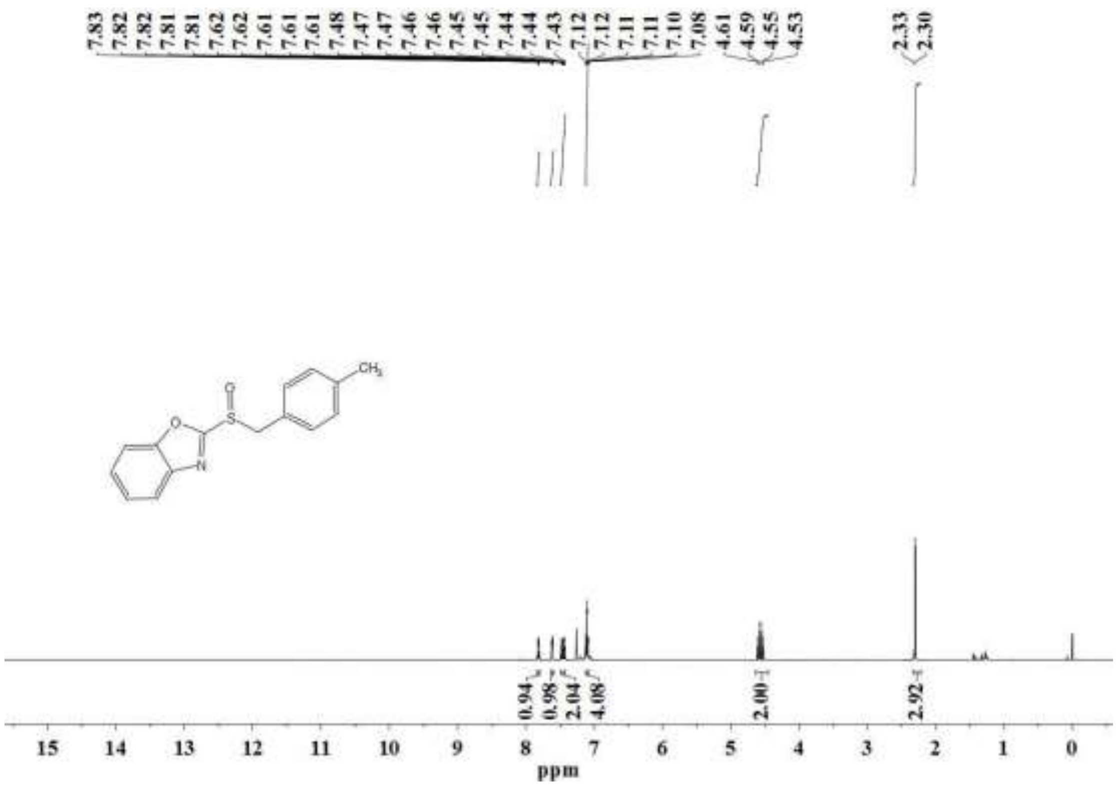

Figure S 19-1. ^{1}H NMR spectrum of compound **5g**.

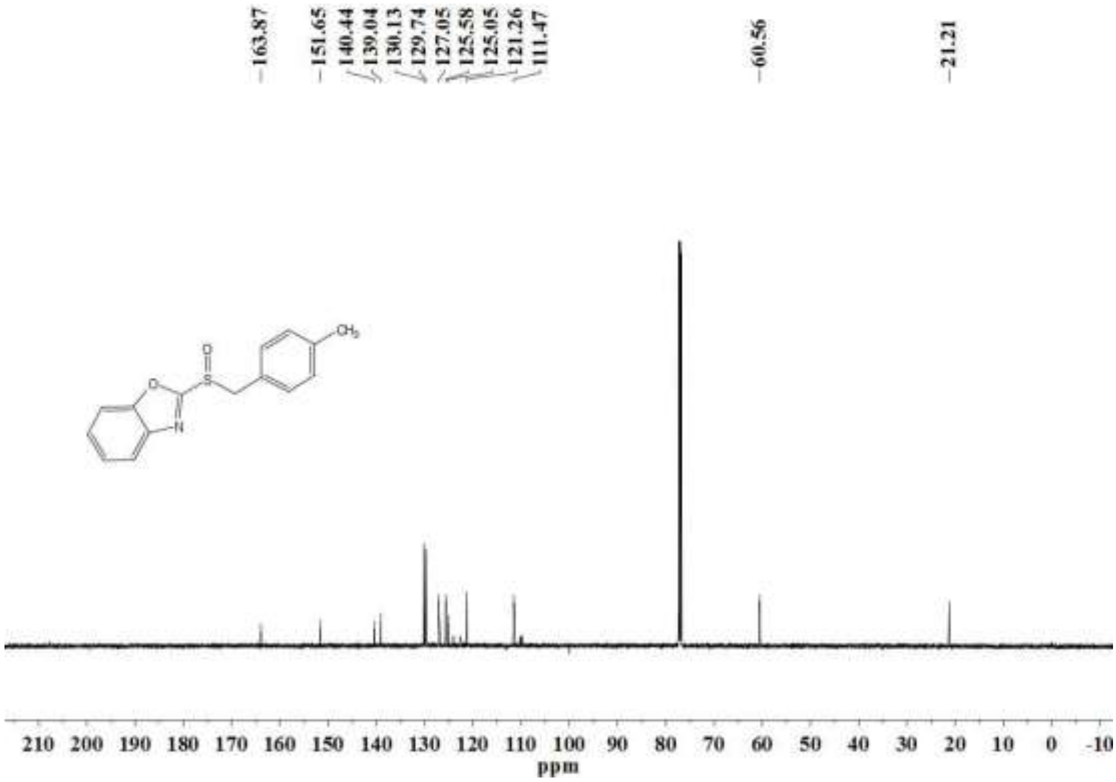

Figure S 19-2. ^{13}C NMR spectrum of compound **5g**.

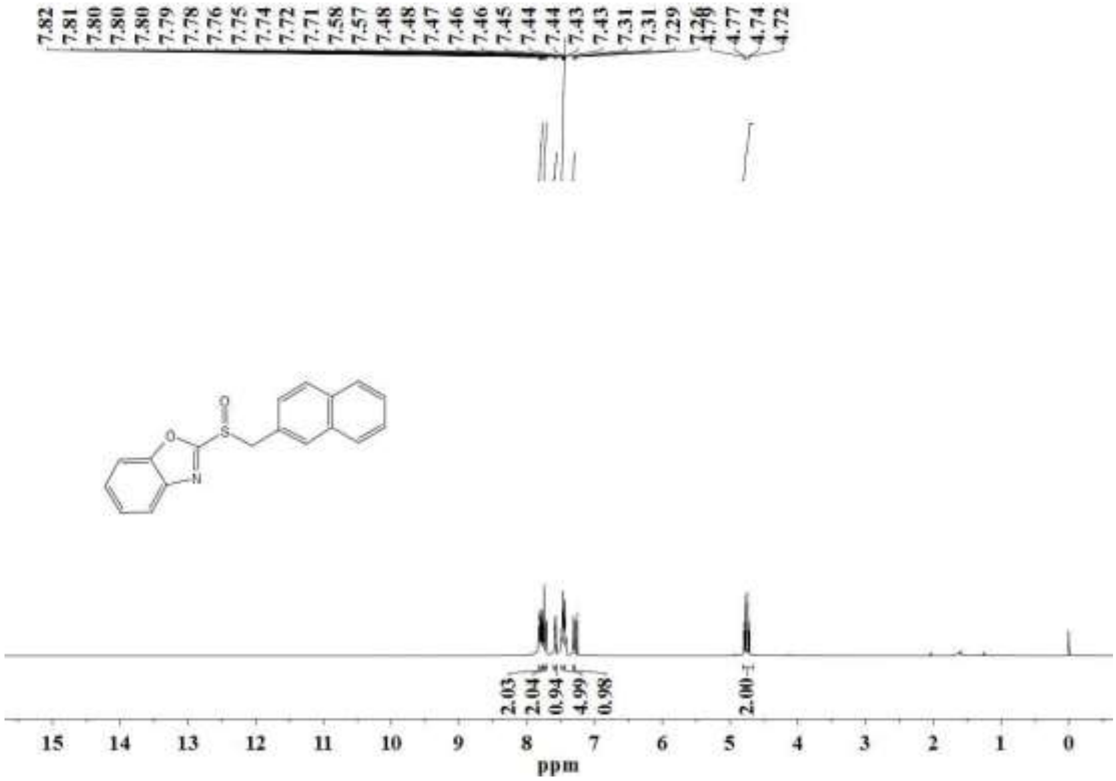

Figure S 20-1. ^{1}H NMR spectrum of compound **5h**.

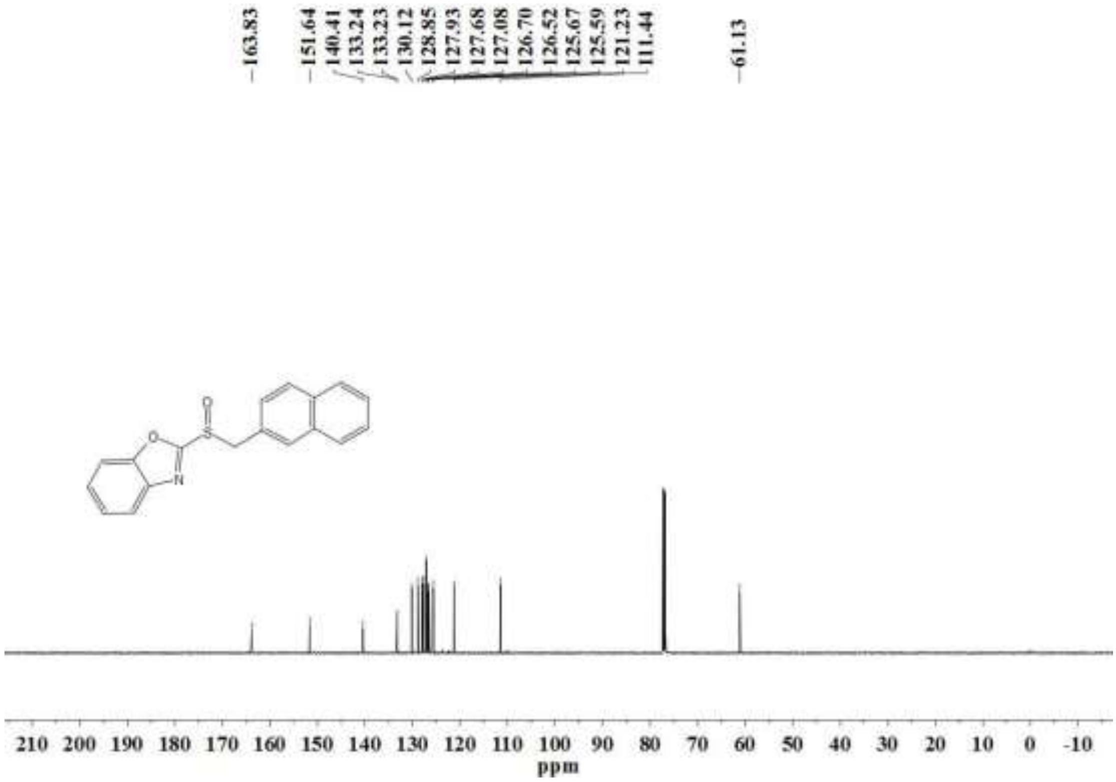

Figure S 20-2. ^{13}C NMR spectrum of compound **5h**.

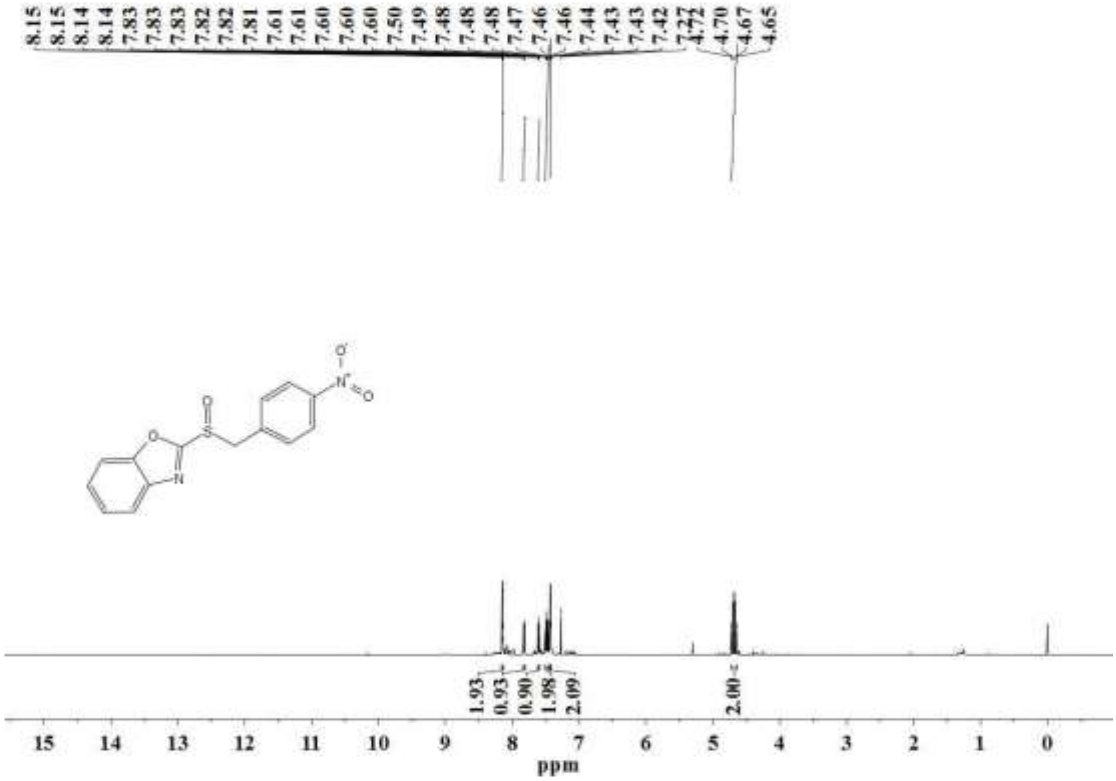

Figure S 21-1. ^{1}H NMR spectrum of compound **5i**.

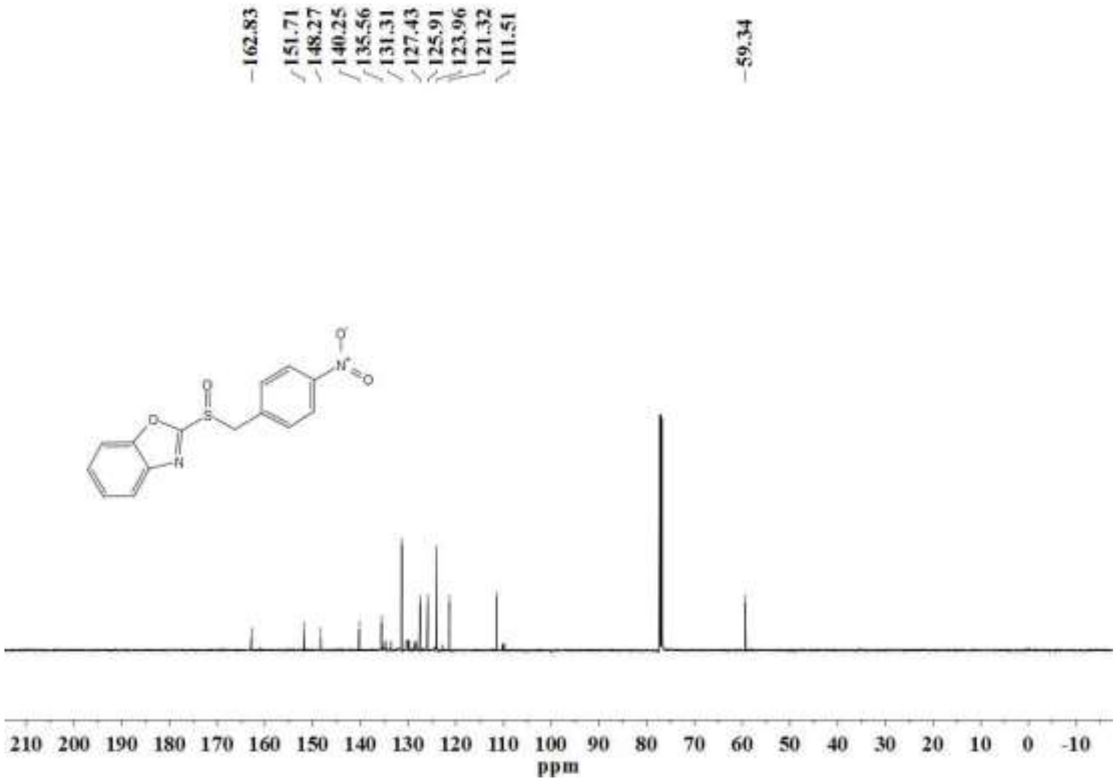

Figure S 21-2. ^{13}C NMR spectrum of compound **5i**.

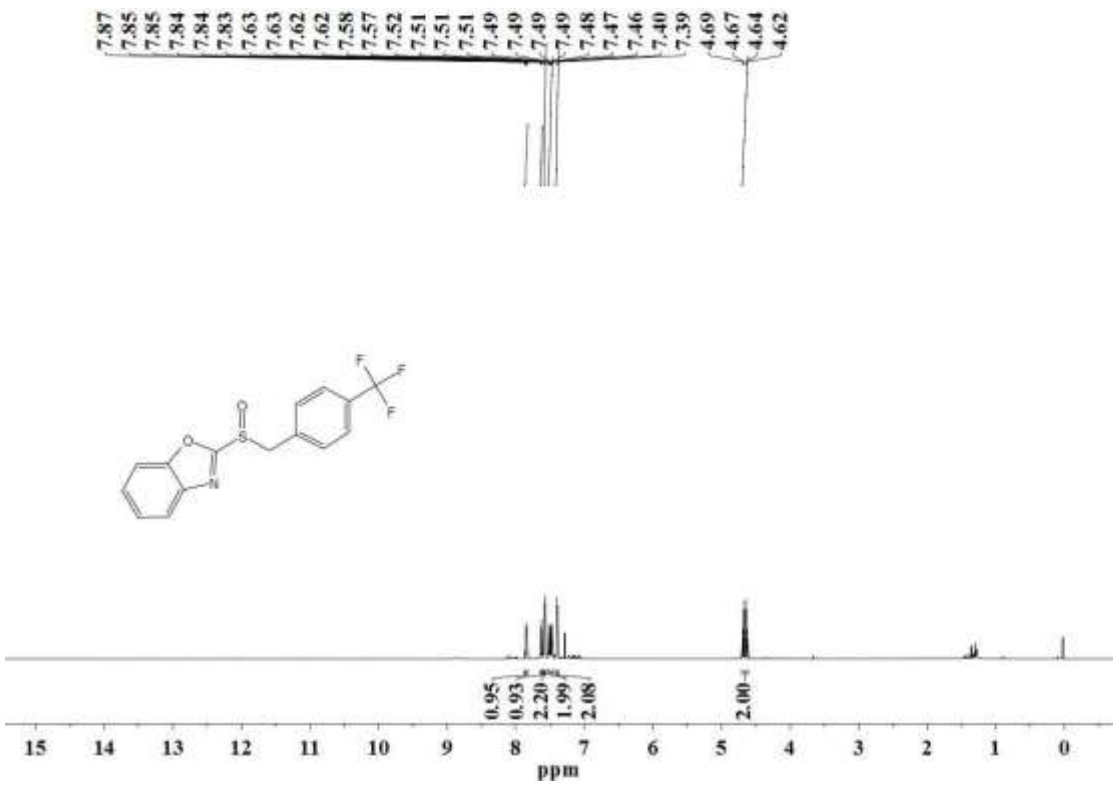

Figure S 22-1. ^{1}H NMR spectrum of compound **5j**.

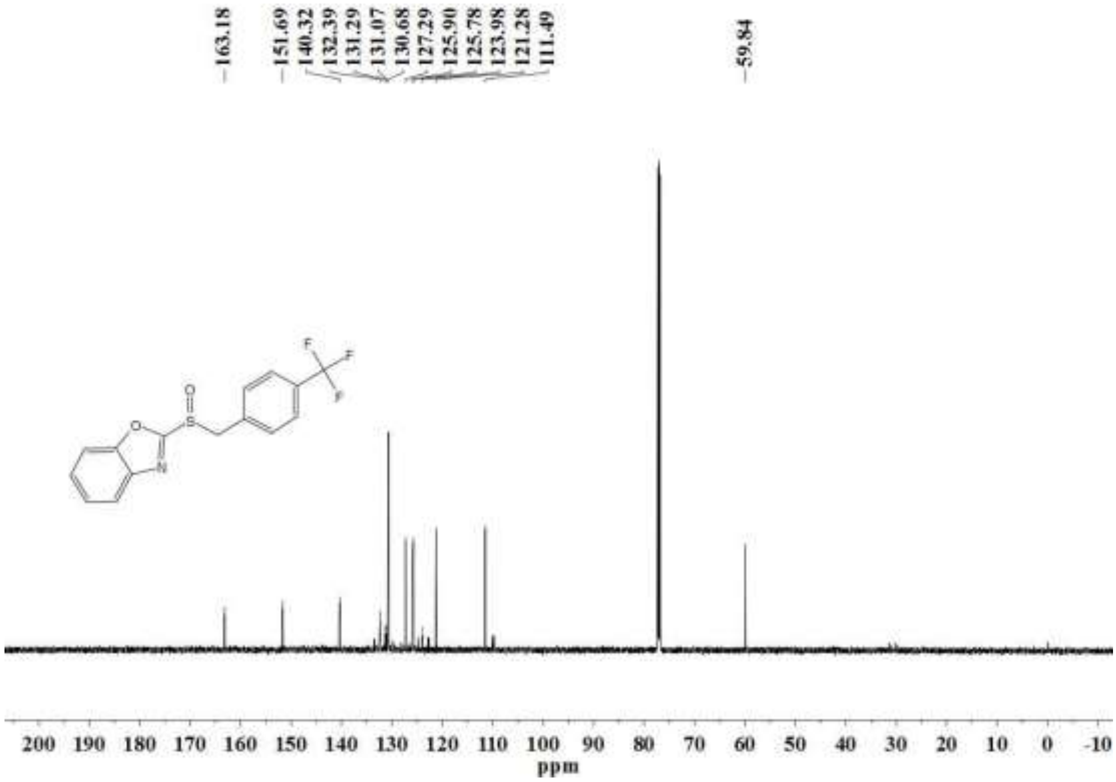

Figure S 22-2. ^{13}C NMR spectrum of compound **5j**.

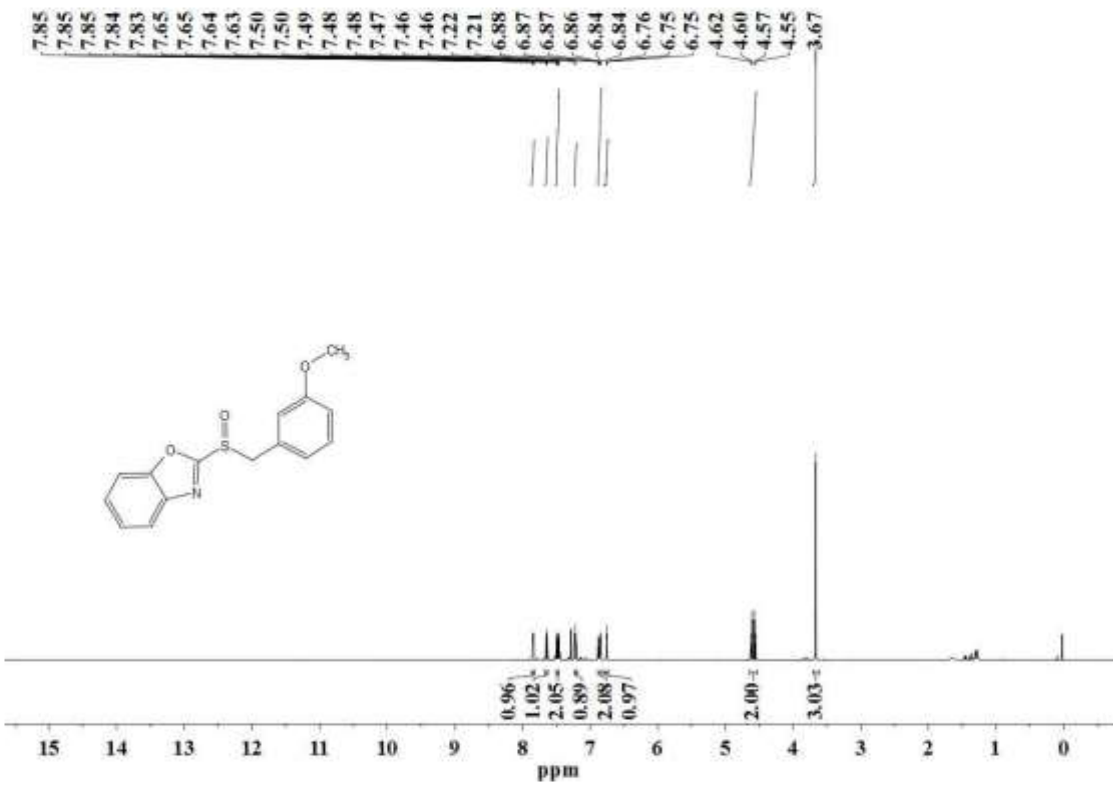

Figure S 23-1. ^{1}H NMR spectrum of compound **5k**.

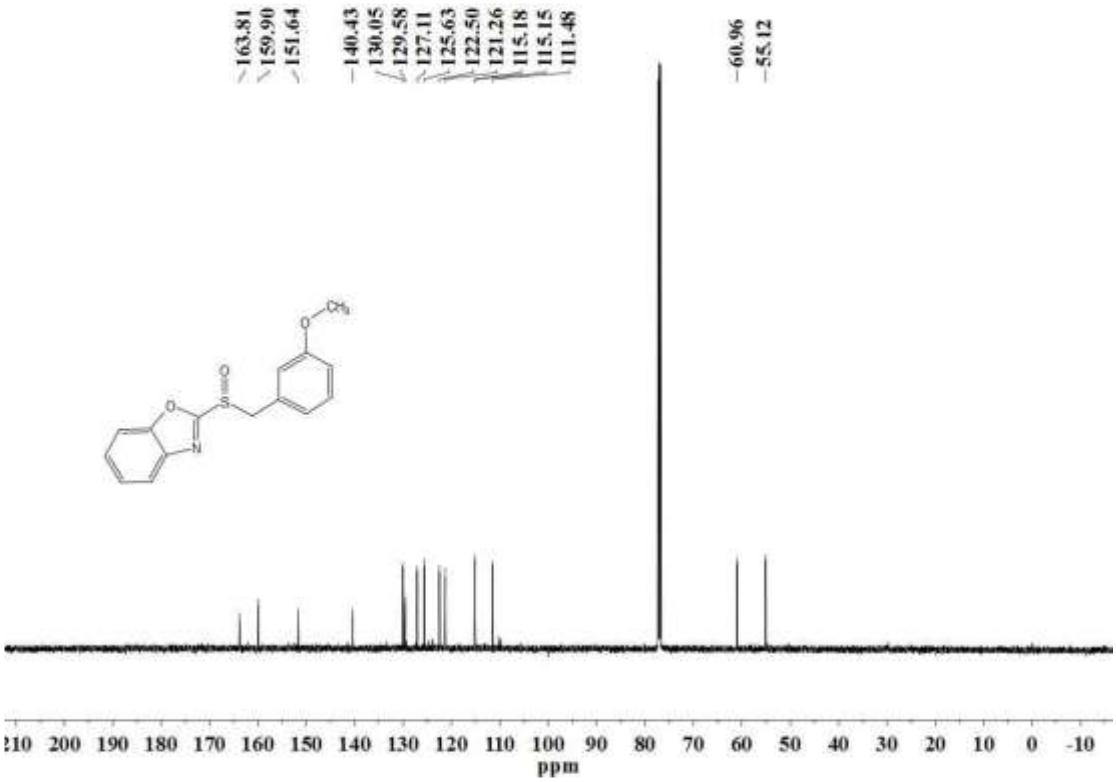

Figure S 23-2. ^{13}C NMR spectrum of compound **5k**.

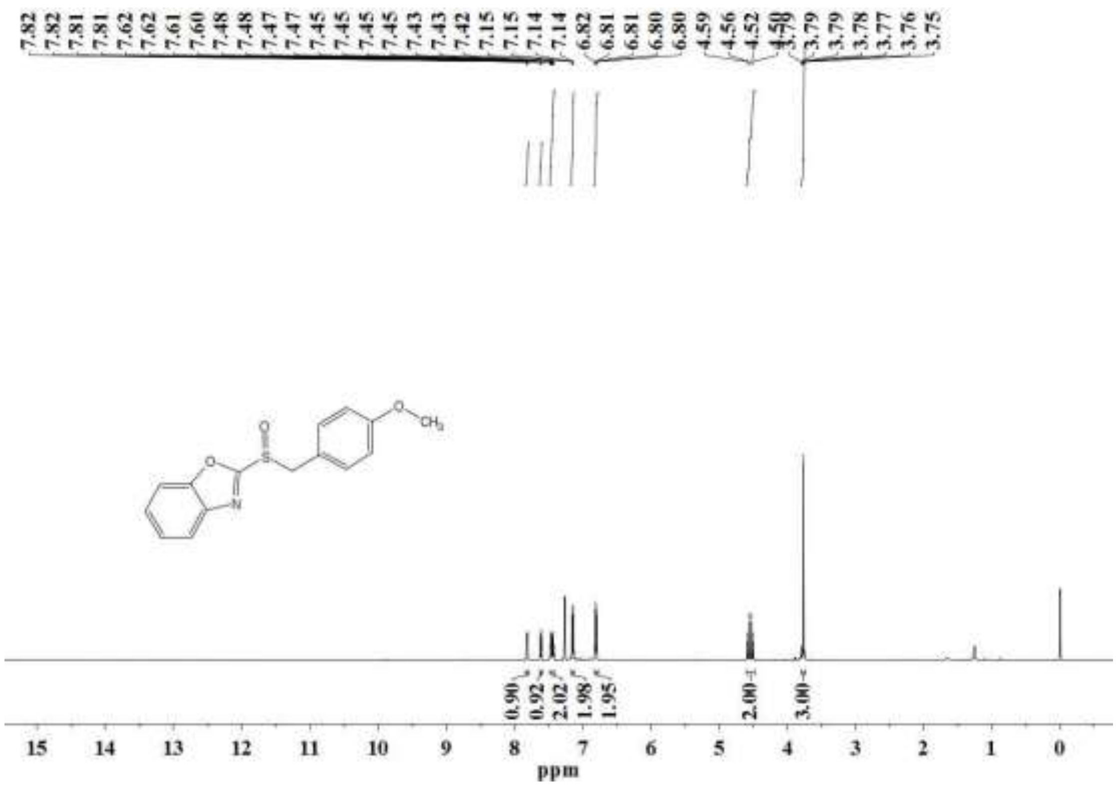

Figure S 24-1. ^{1}H NMR spectrum of compound **5l**.

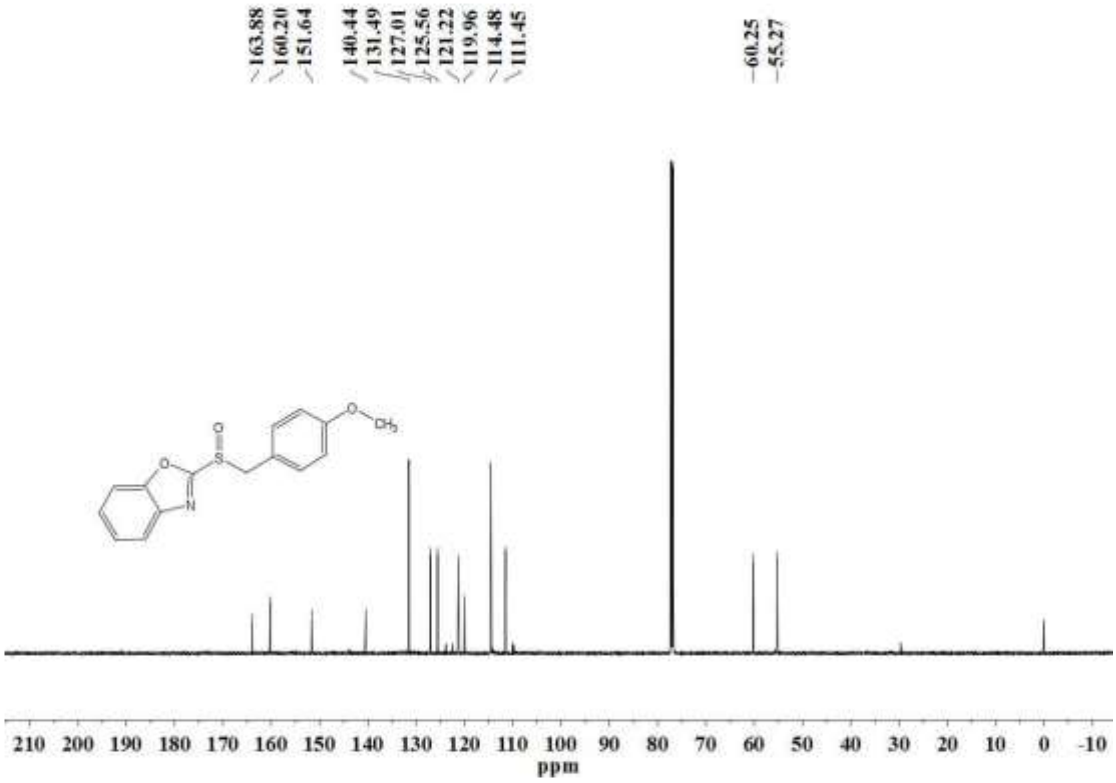

Figure S 24-2. ^{13}C NMR spectrum of compound **5l**.

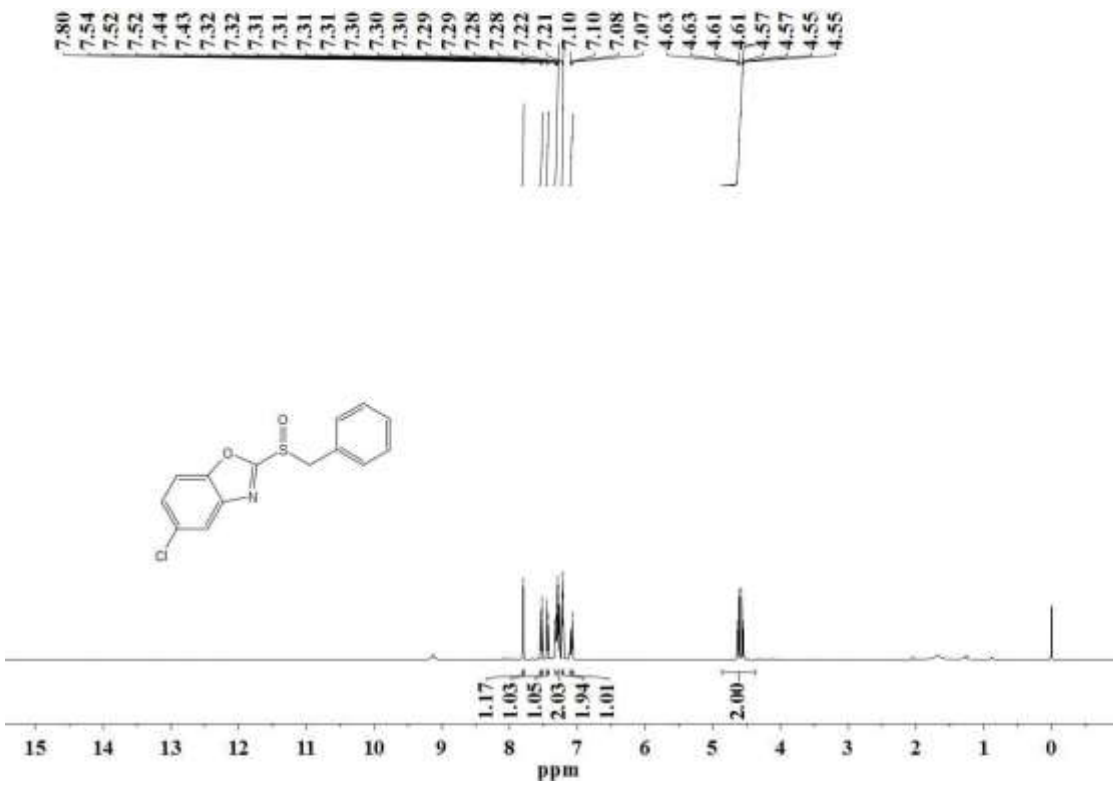

Figure S 25-1. ^{1}H NMR spectrum of compound **6a**.

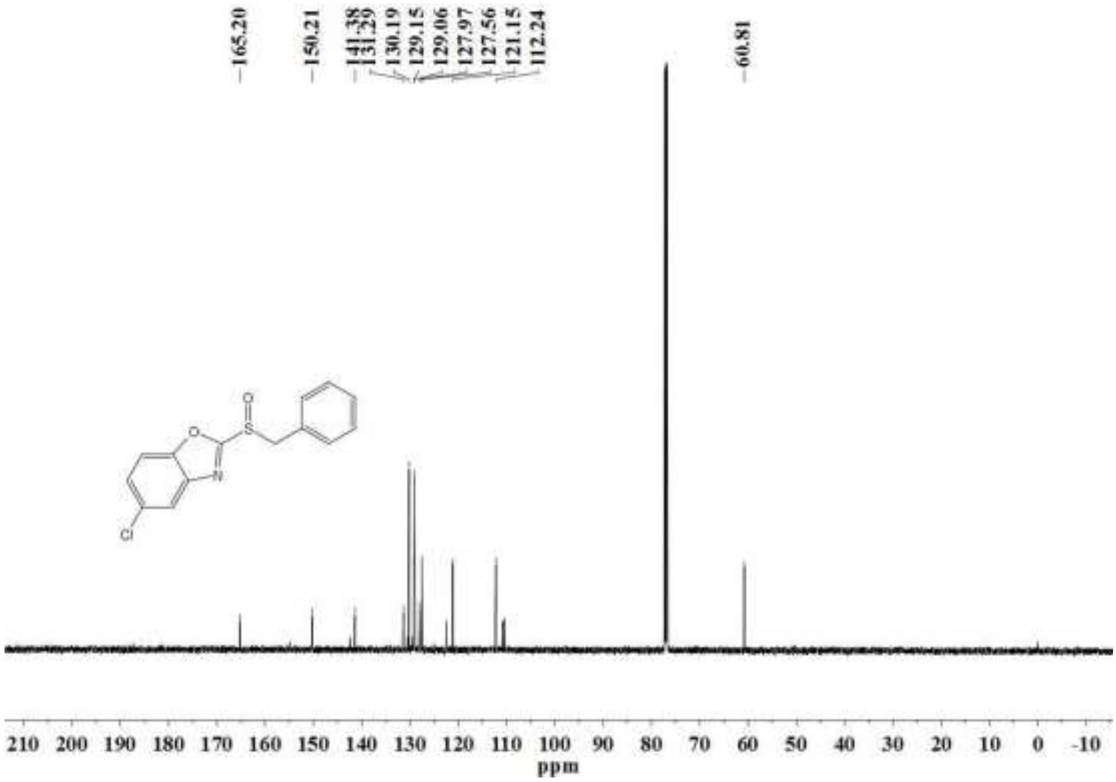

Figure S 25-2. ^{13}C NMR spectrum of compound **6a**.

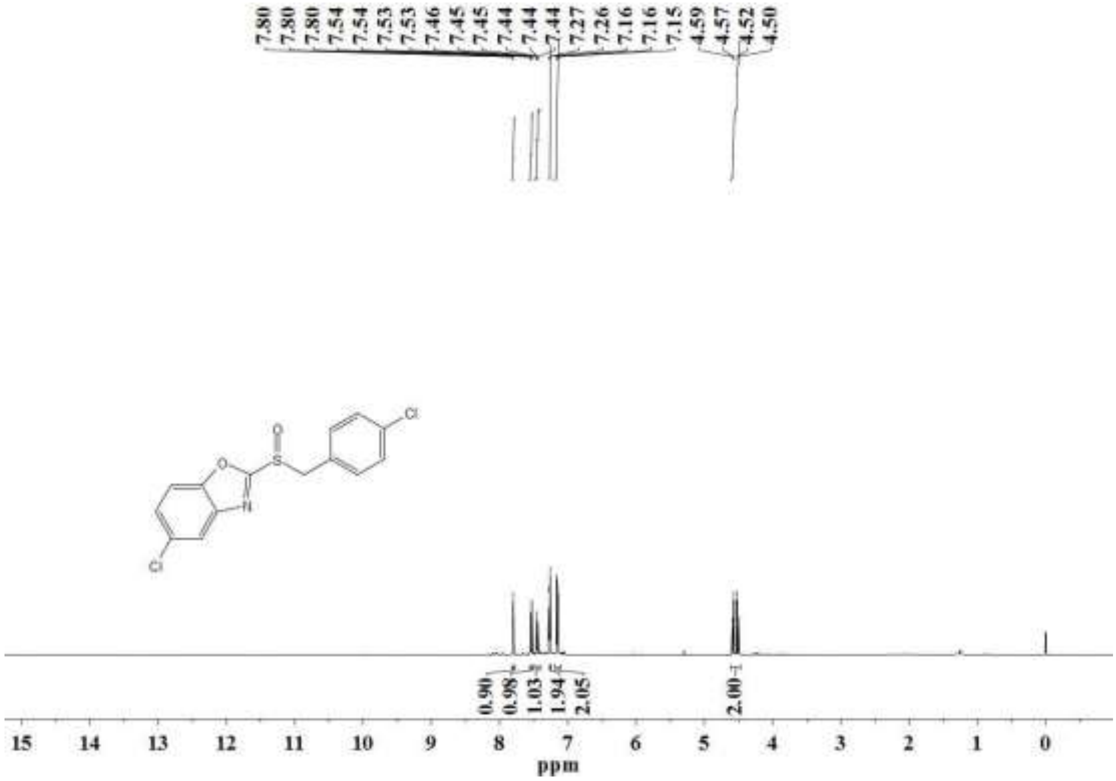

Figure S 26-1. ^{1}H NMR spectrum of compound **6b**.

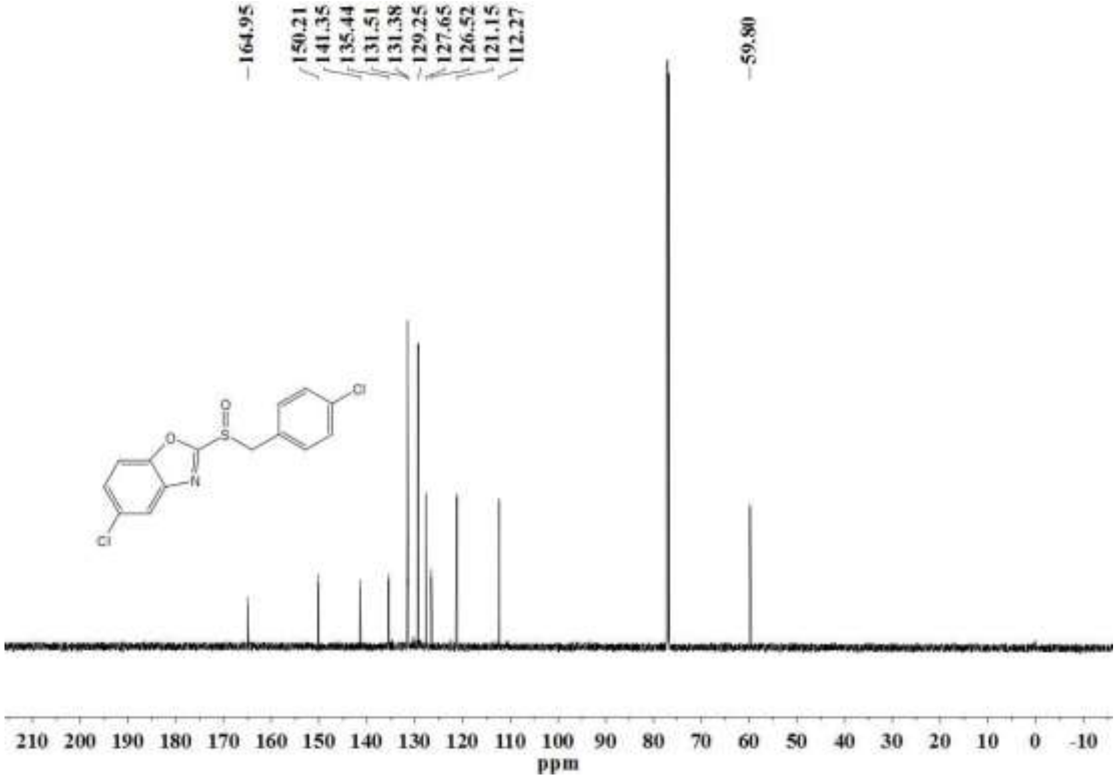

Figure S 26-2. ^{13}C NMR spectrum of compound **6b**.

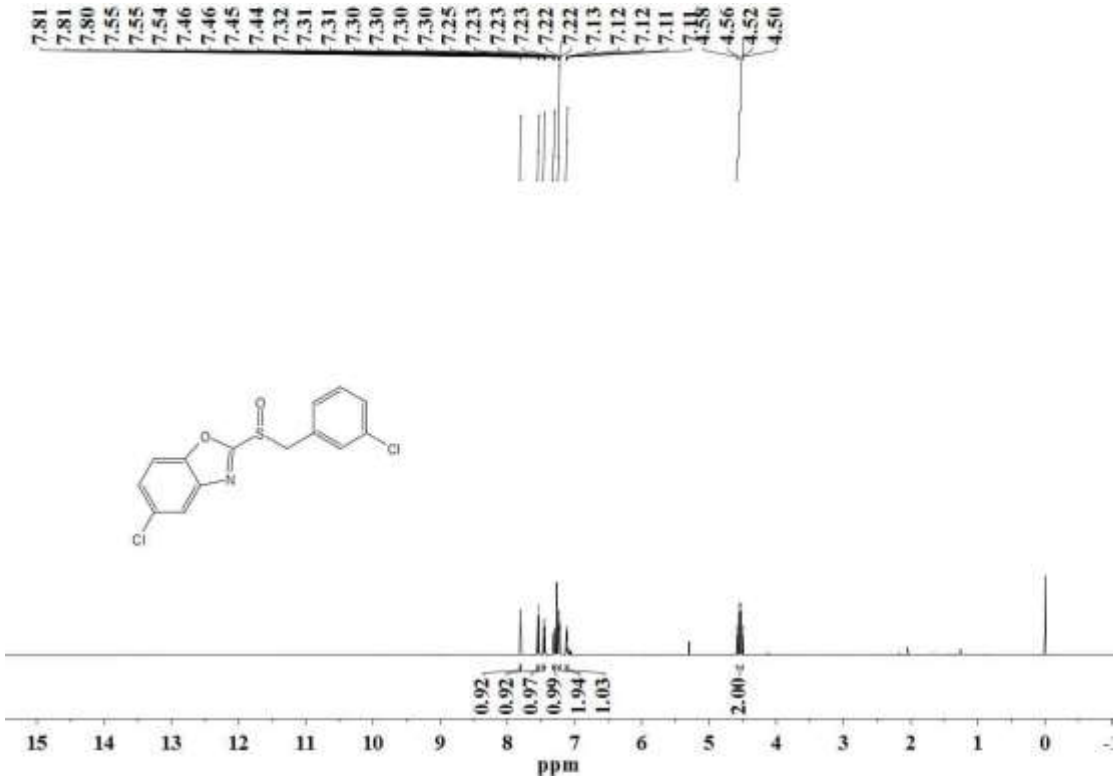

Figure S 27-1. ^{1}H NMR spectrum of compound **6c**.

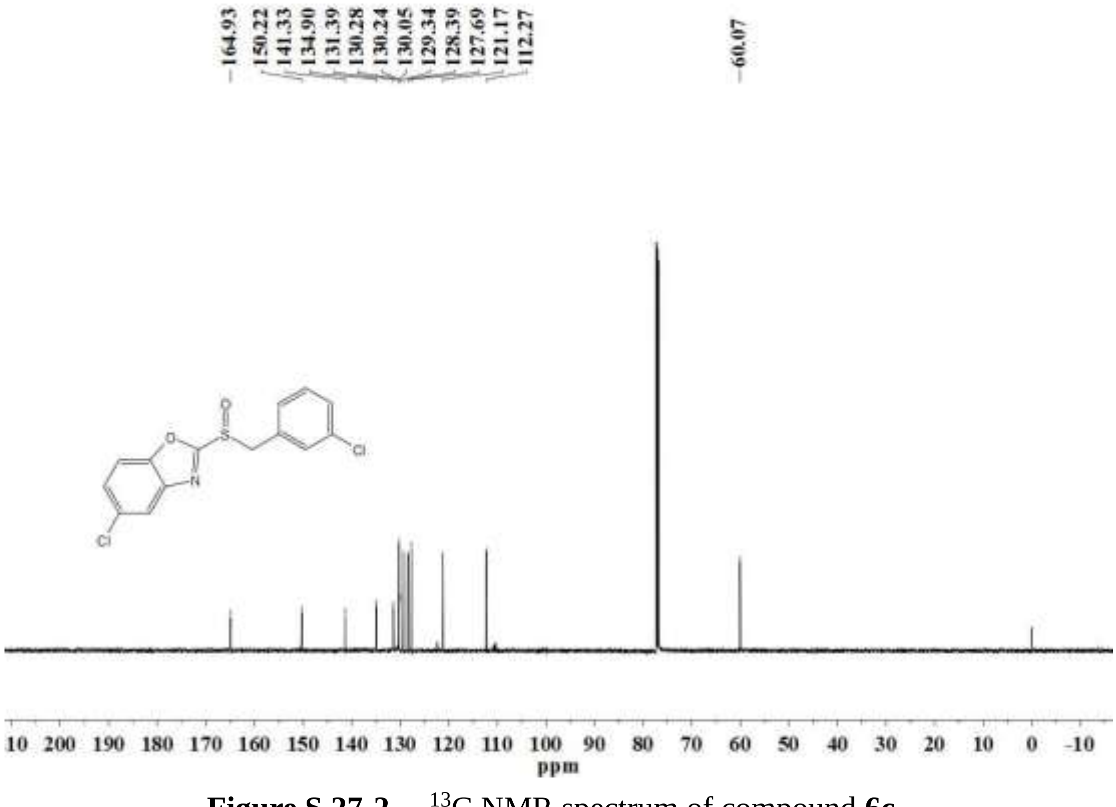

Figure S 27-2. ^{13}C NMR spectrum of compound **6c**.

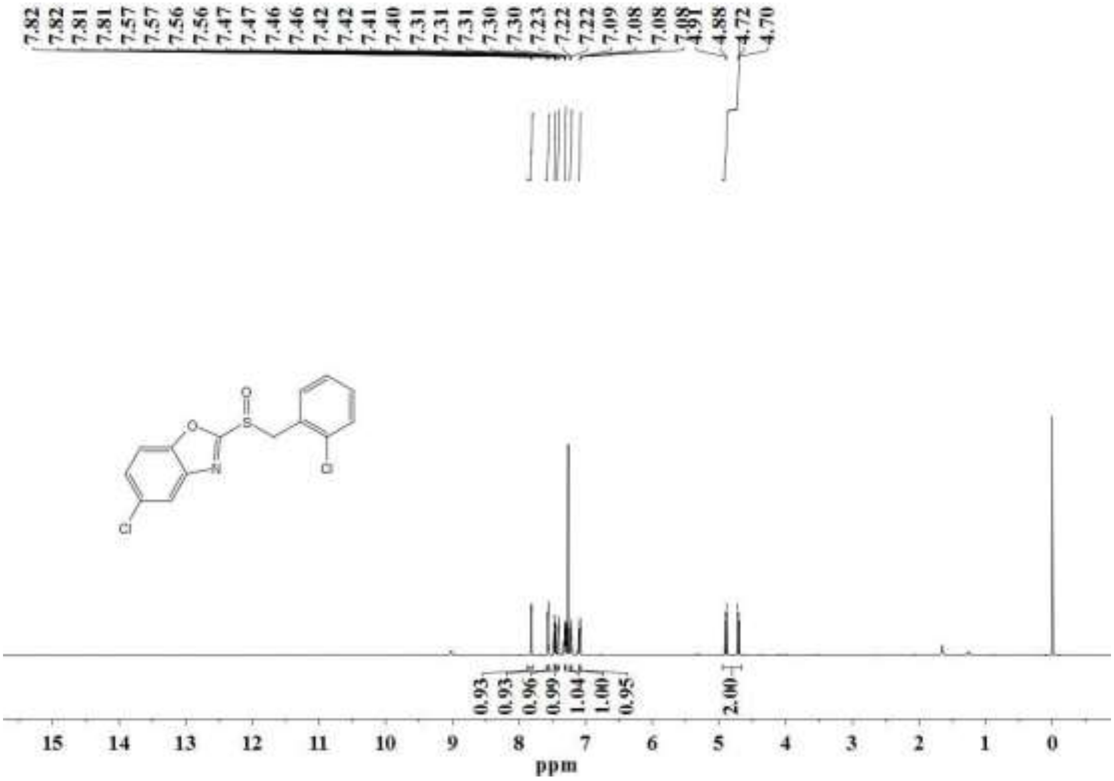

Figure S 28-1. ^{1}H NMR spectrum of compound **6d**.

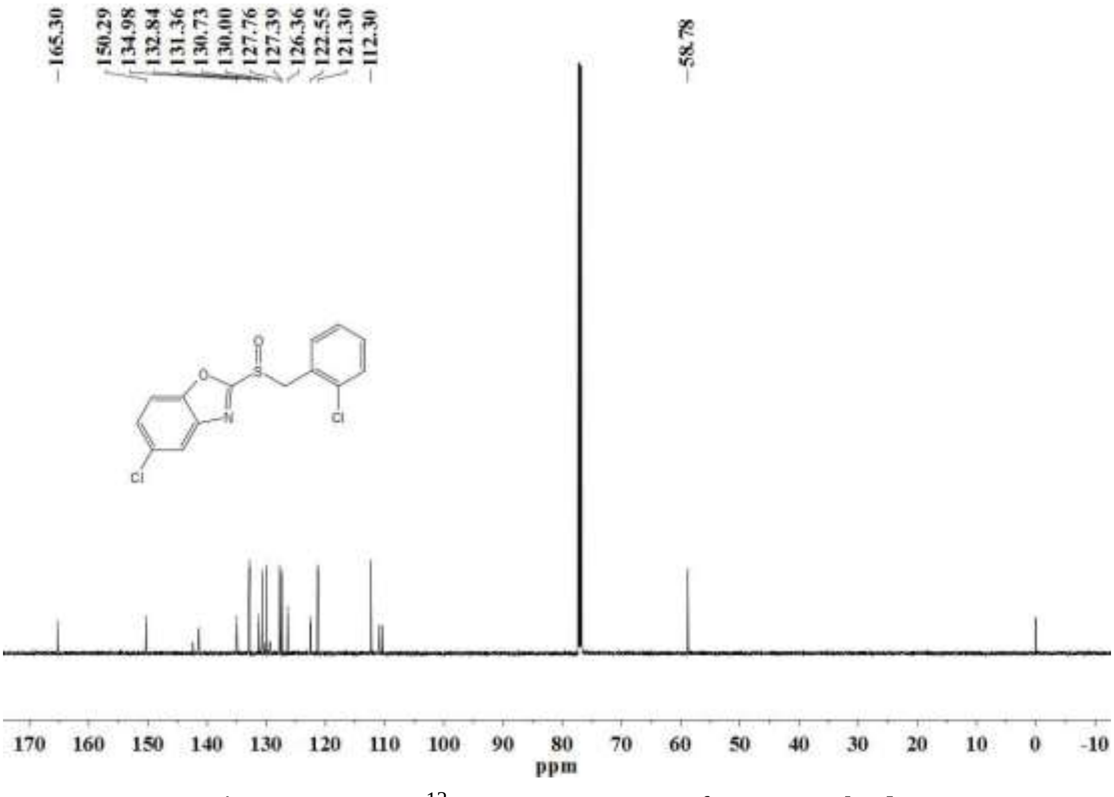

Figure S 28-2. ^{13}C NMR spectrum of compound **6d**.

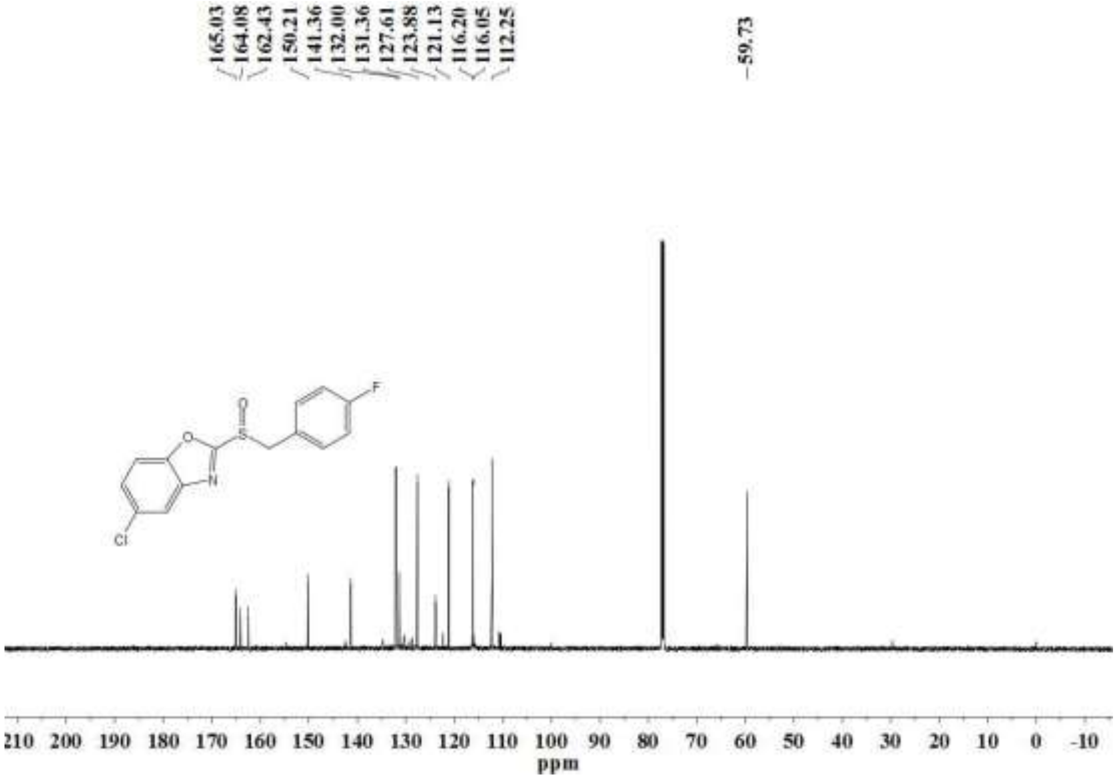

Figure S 29-1. ¹H NMR spectrum of compound **6e**

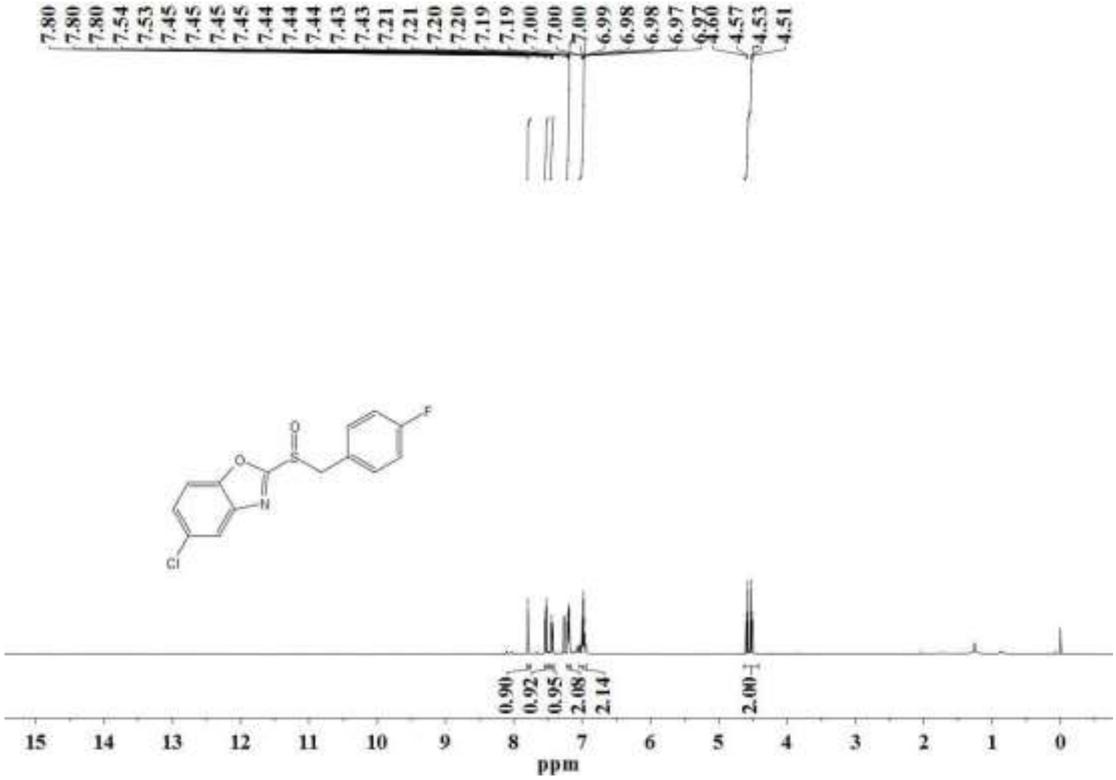

Figure S 29-2. ¹³C NMR spectrum of compound **6e**.

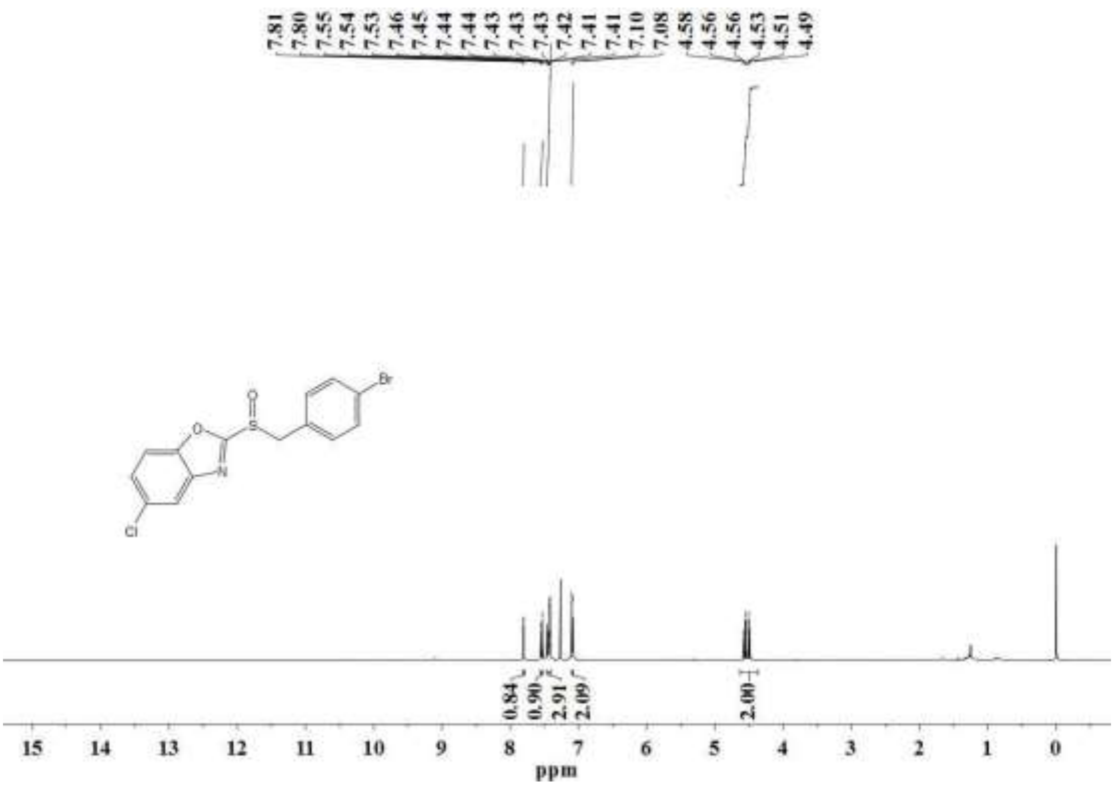

Figure S 30-1. ^{1}H NMR spectrum of compound **6f**.

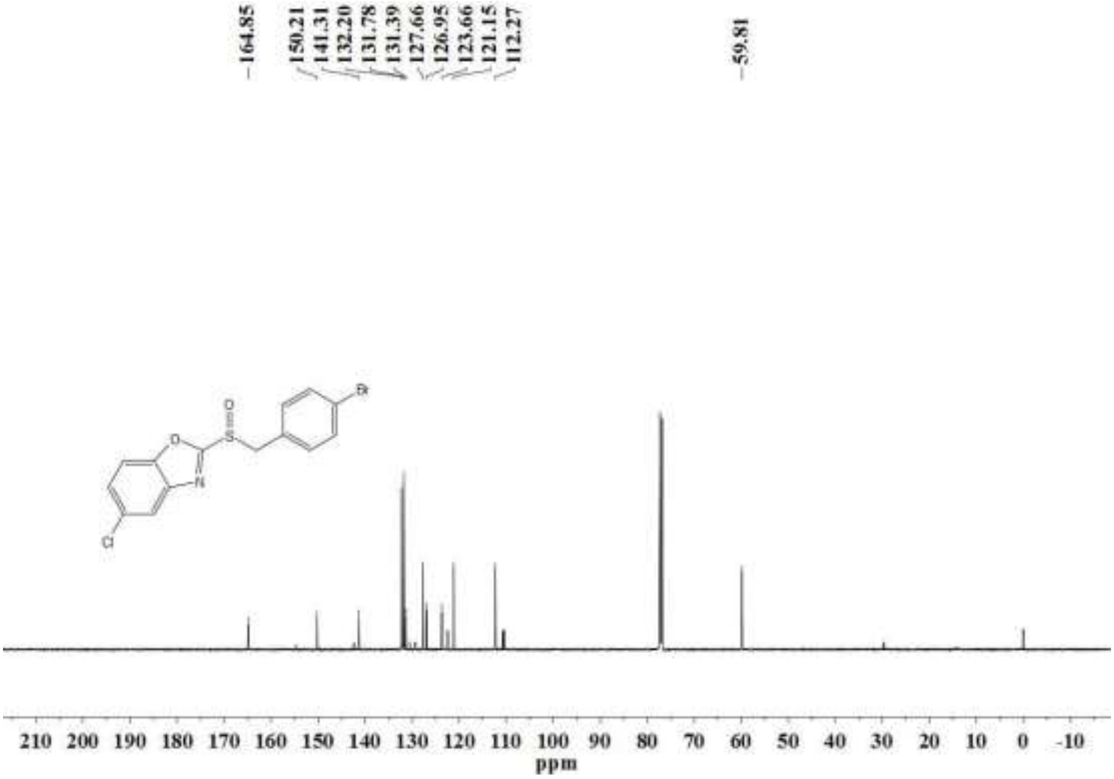

Figure S 30-2. ^{13}C NMR spectrum of compound **6f**.

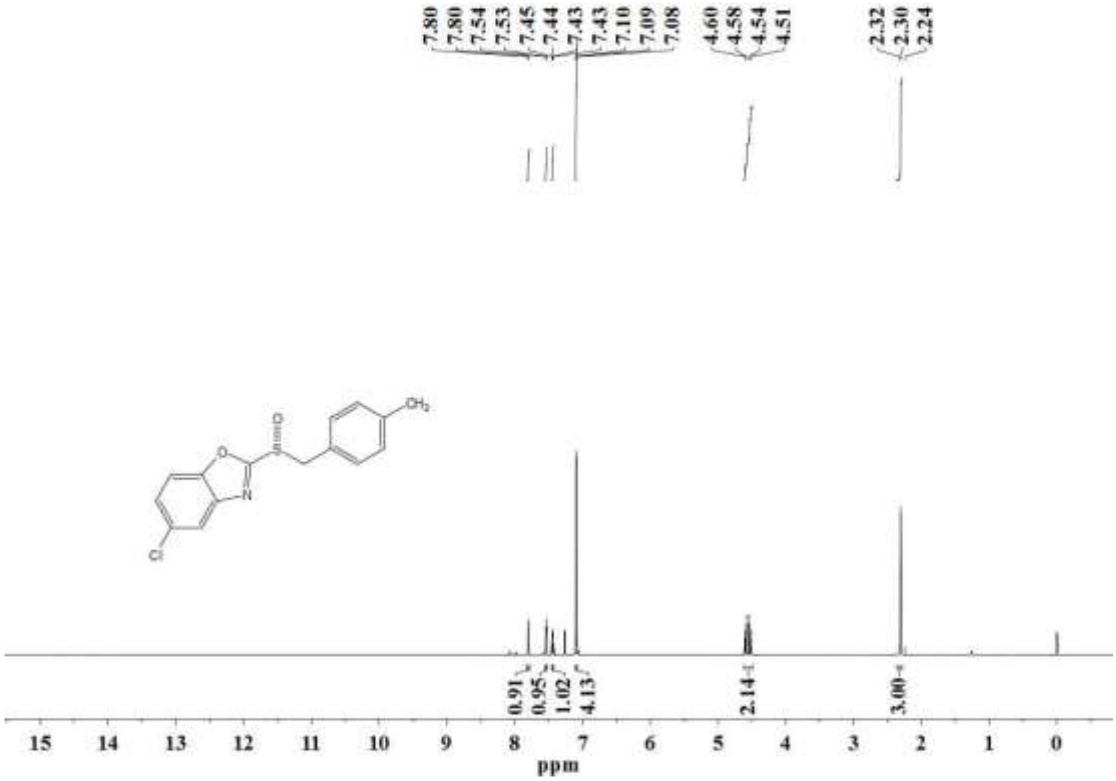

Figure S 31-1. ^{1}H NMR spectrum of compound **6g**.

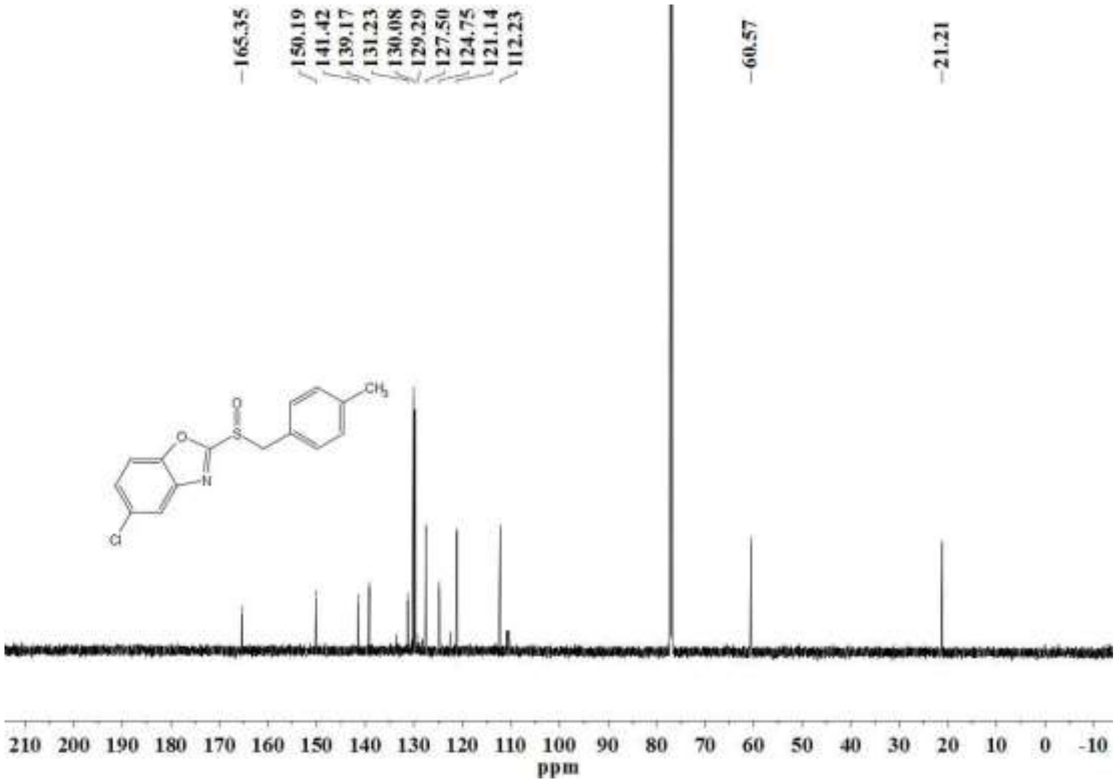

Figure S 31-2. ^{13}C NMR spectrum of compound **6g**.

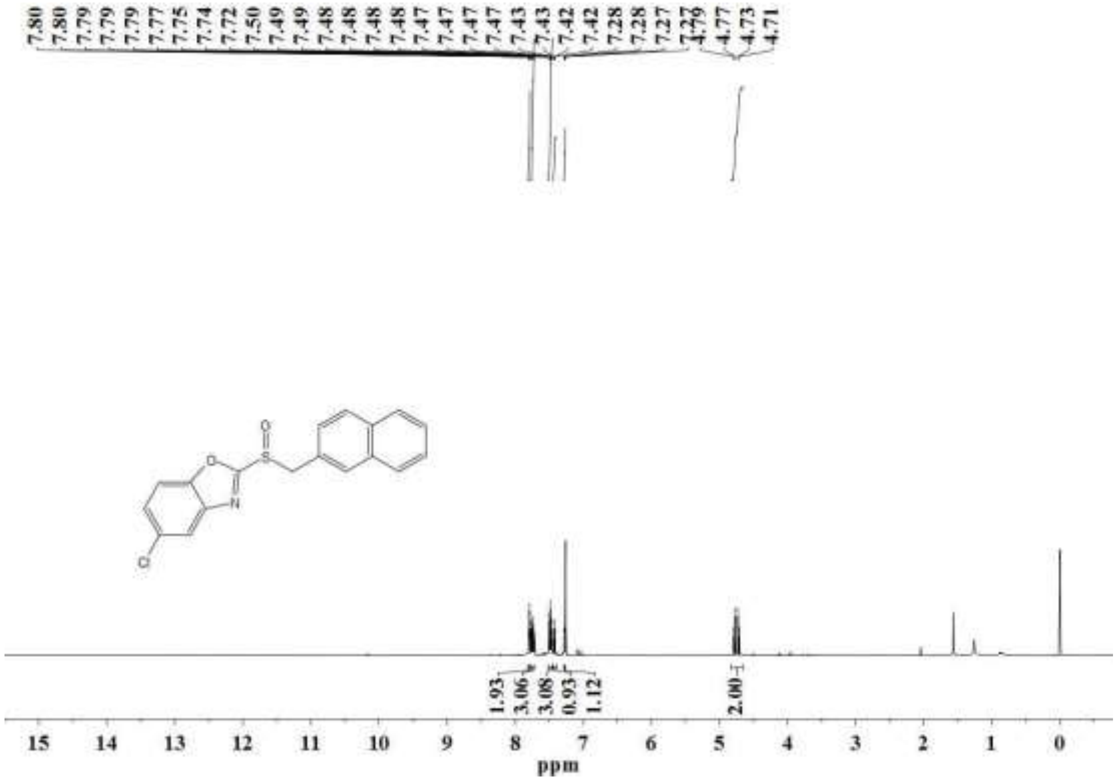

Figure S 32-1. ^{1}H NMR spectrum of compound **6h**.

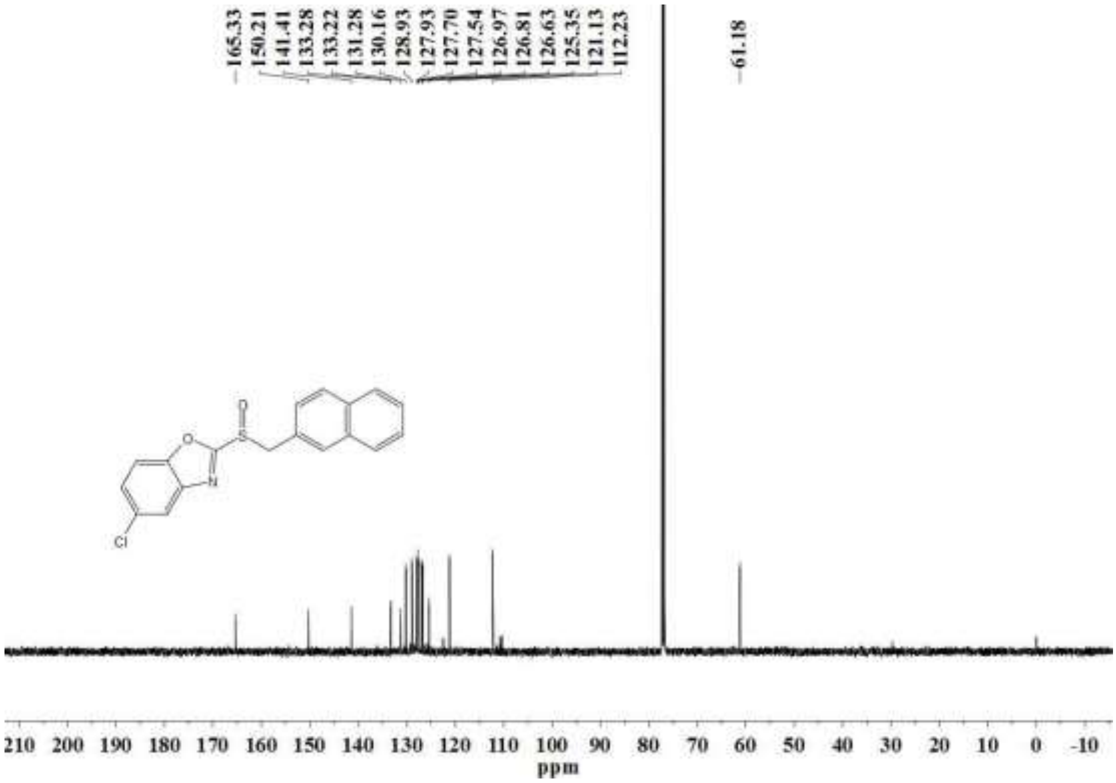

Figure S 32-2. ^{13}C NMR spectrum of compound **6h**.

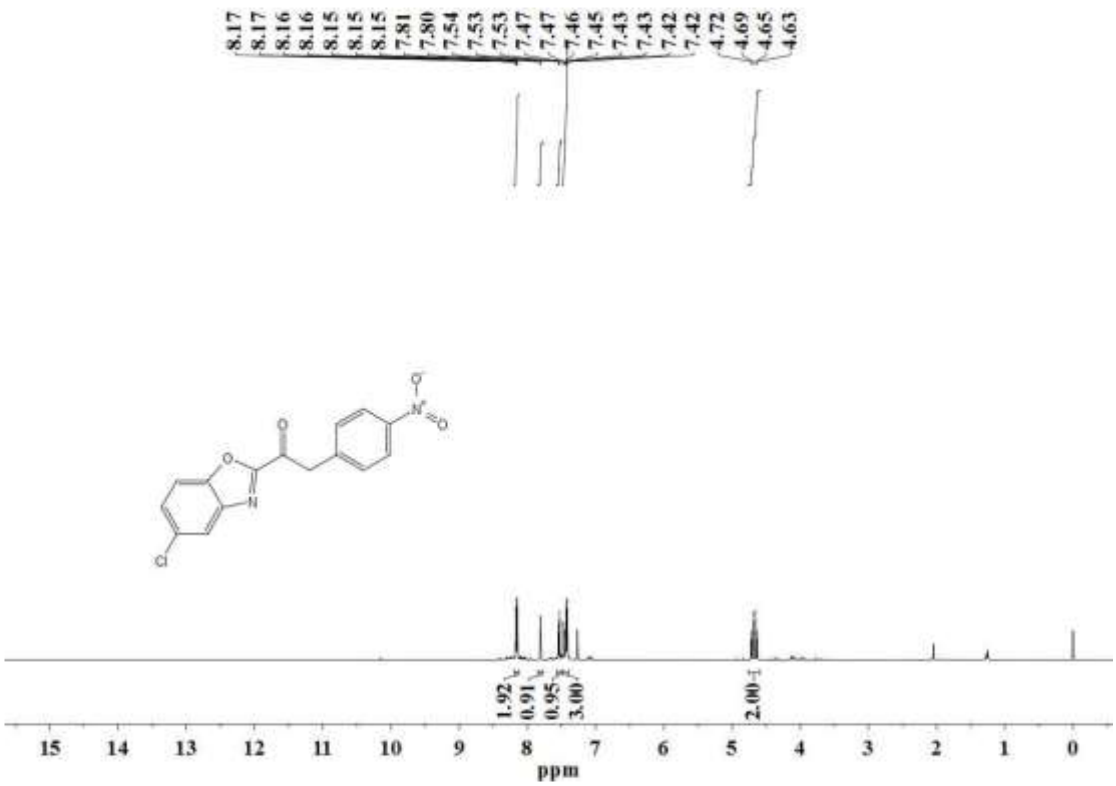

Figure S 33-1. ^{1}H NMR spectrum of compound **6i**.

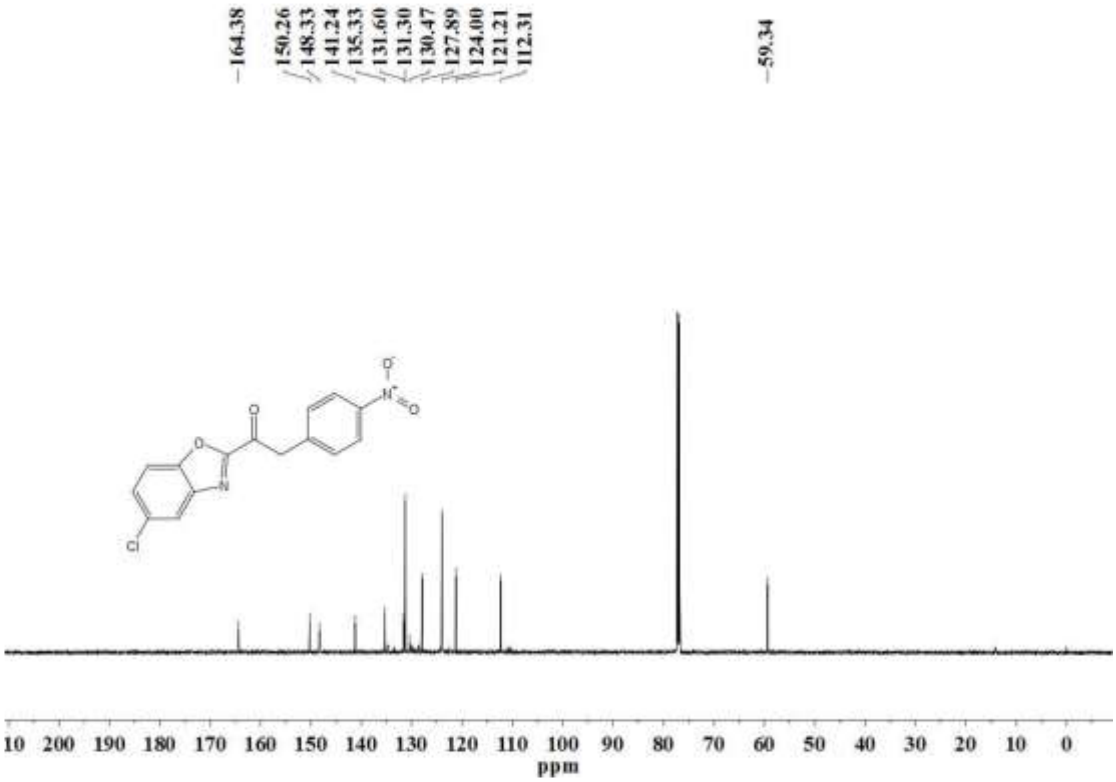

Figure S 33-2. ^{13}C NMR spectrum of compound **6i**.

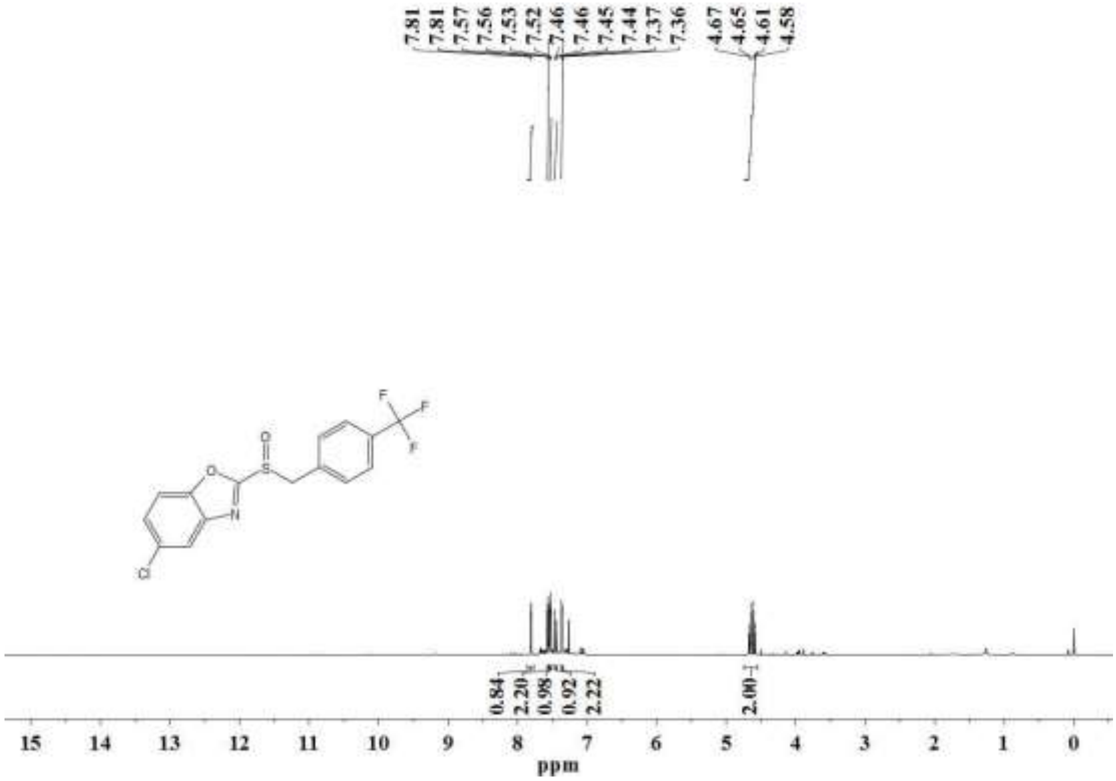

Figure S 34-1. ^{1}H NMR spectrum of compound **6j**.

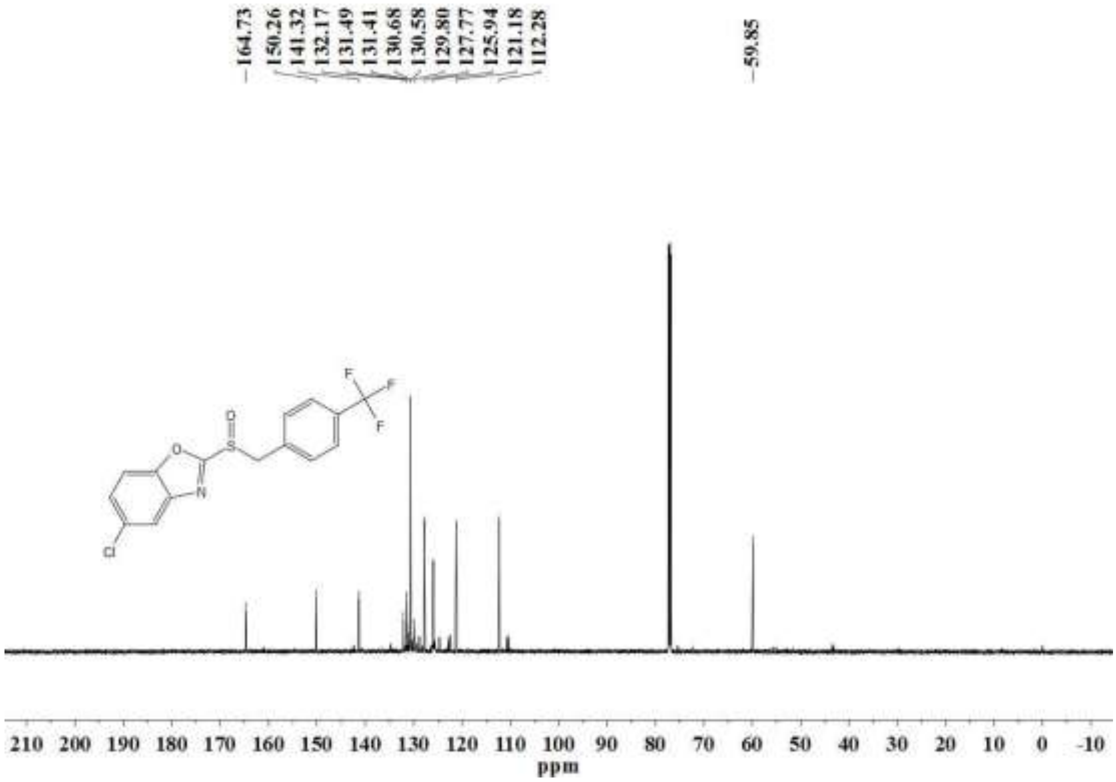

Figure S 34-2. ^{13}C NMR spectrum of compound **6j**.

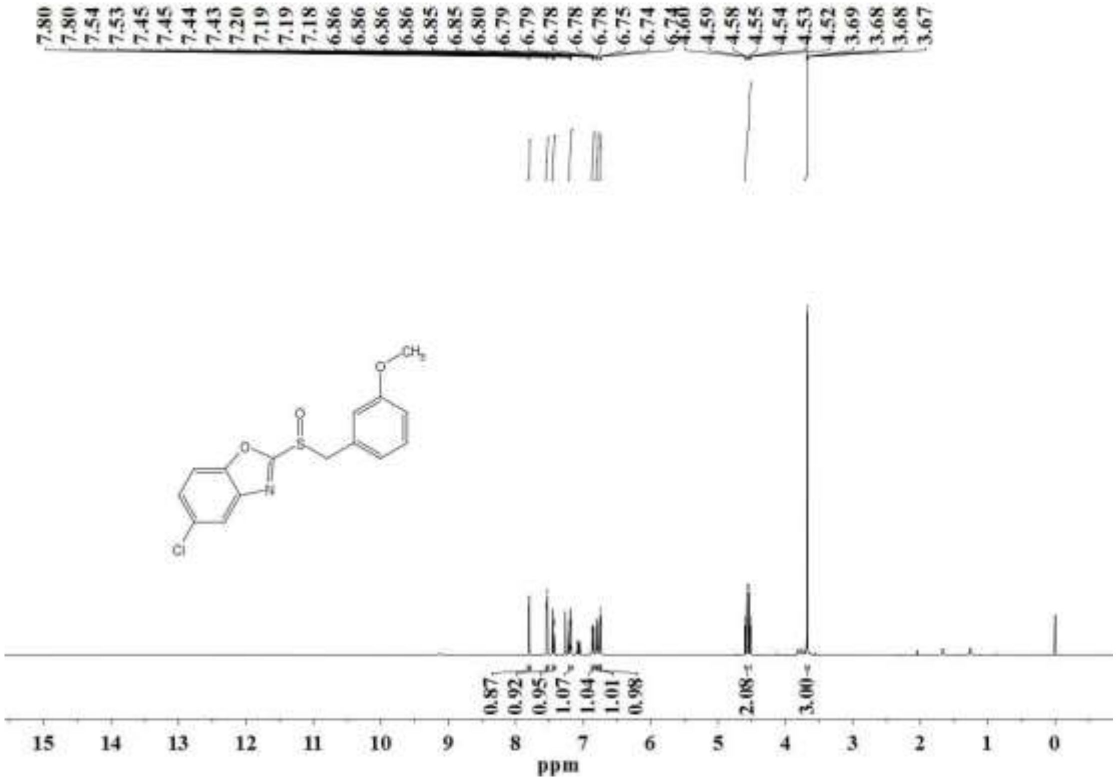

Figure S 35-1. ^{1}H NMR spectrum of compound **6k**.

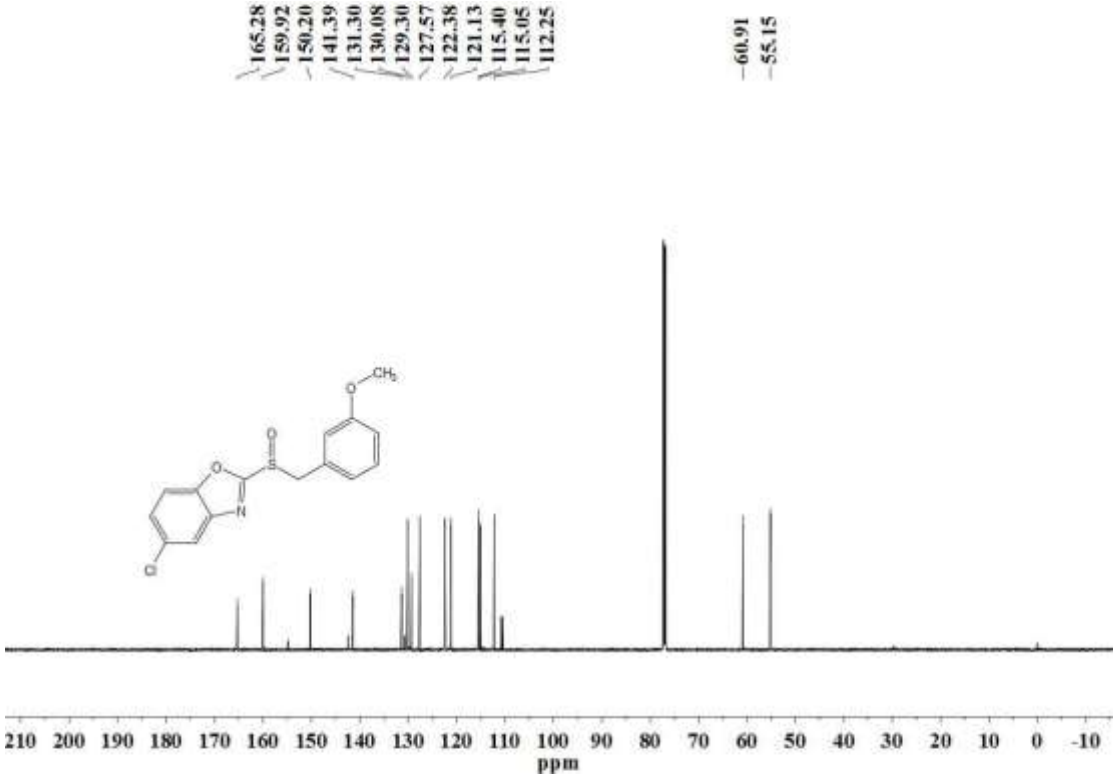

Figure S 35-2. ^{13}C NMR spectrum of compound **6k**.

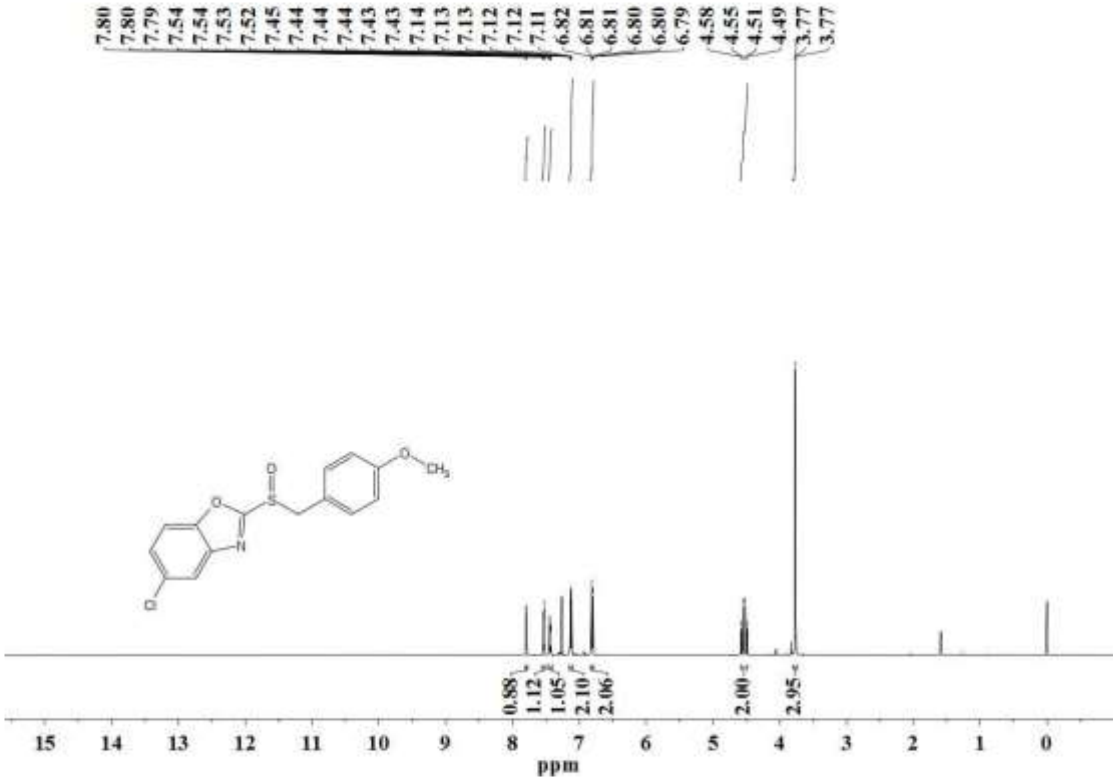

Figure S 36-1. ^{1}H NMR spectrum of compound **6l**.

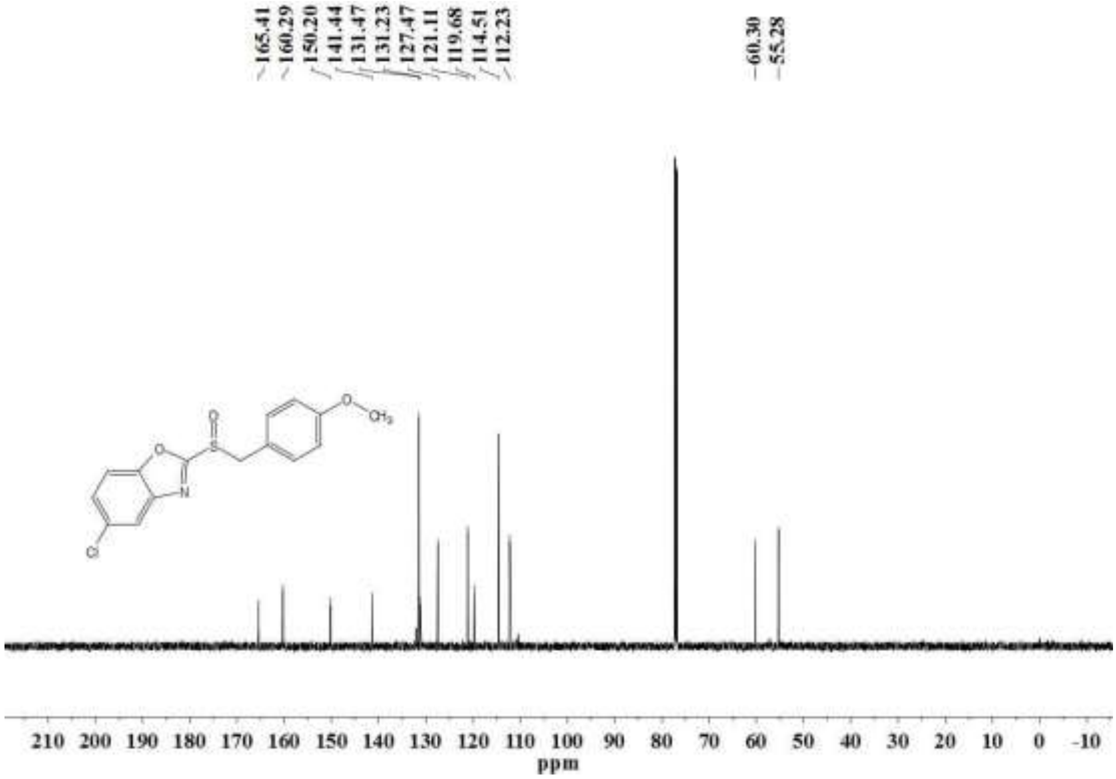

Figure S 36-2. ^{13}C NMR spectrum of compound **6l**.

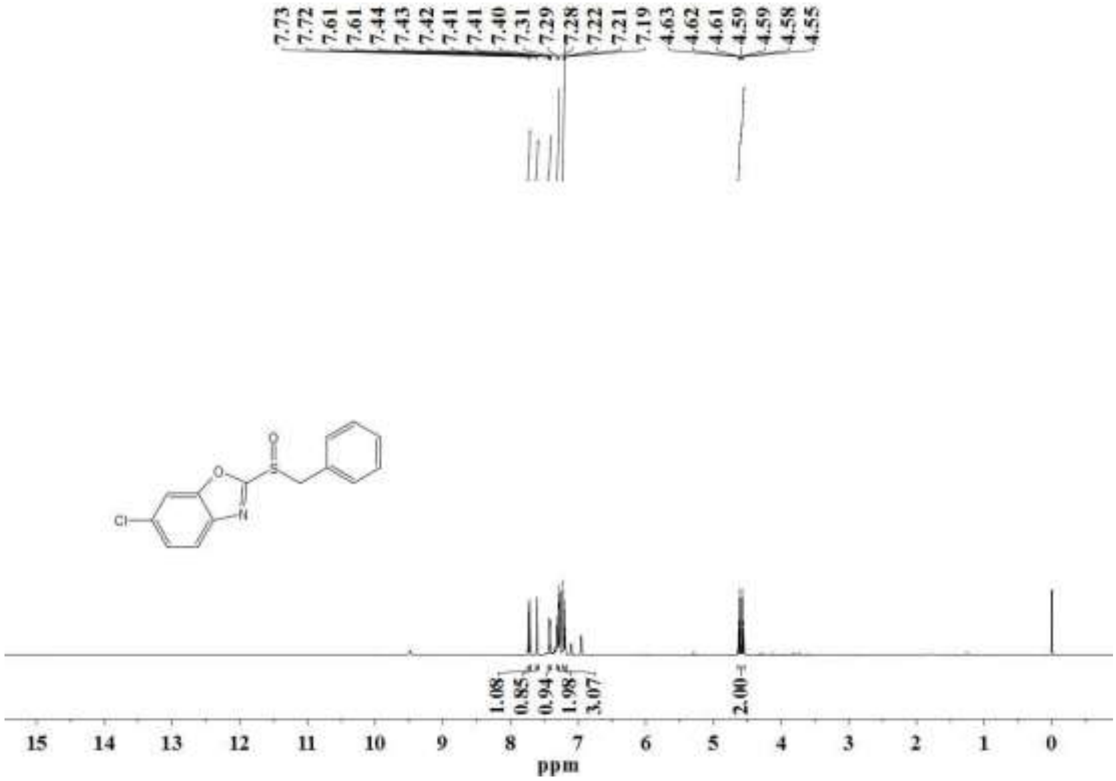

Figure S 37-1. ^{1}H NMR spectrum of compound **7a**.

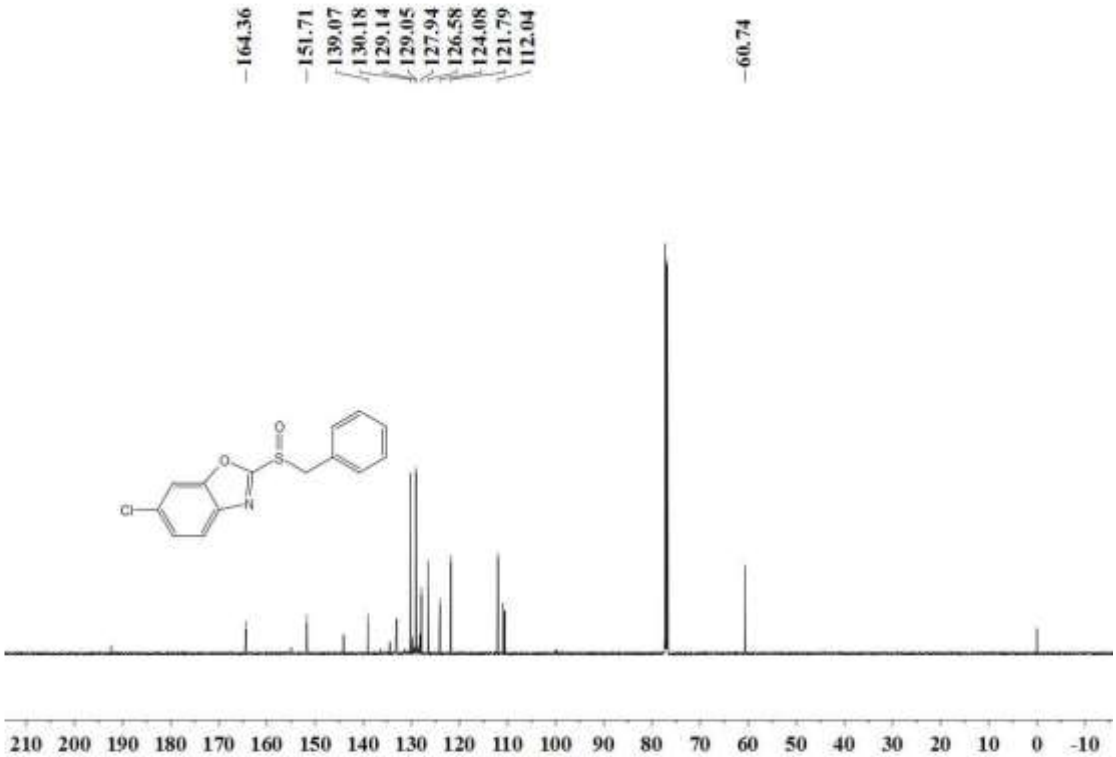

Figure S 37-2. ^{13}C NMR spectrum of compound **7a**.

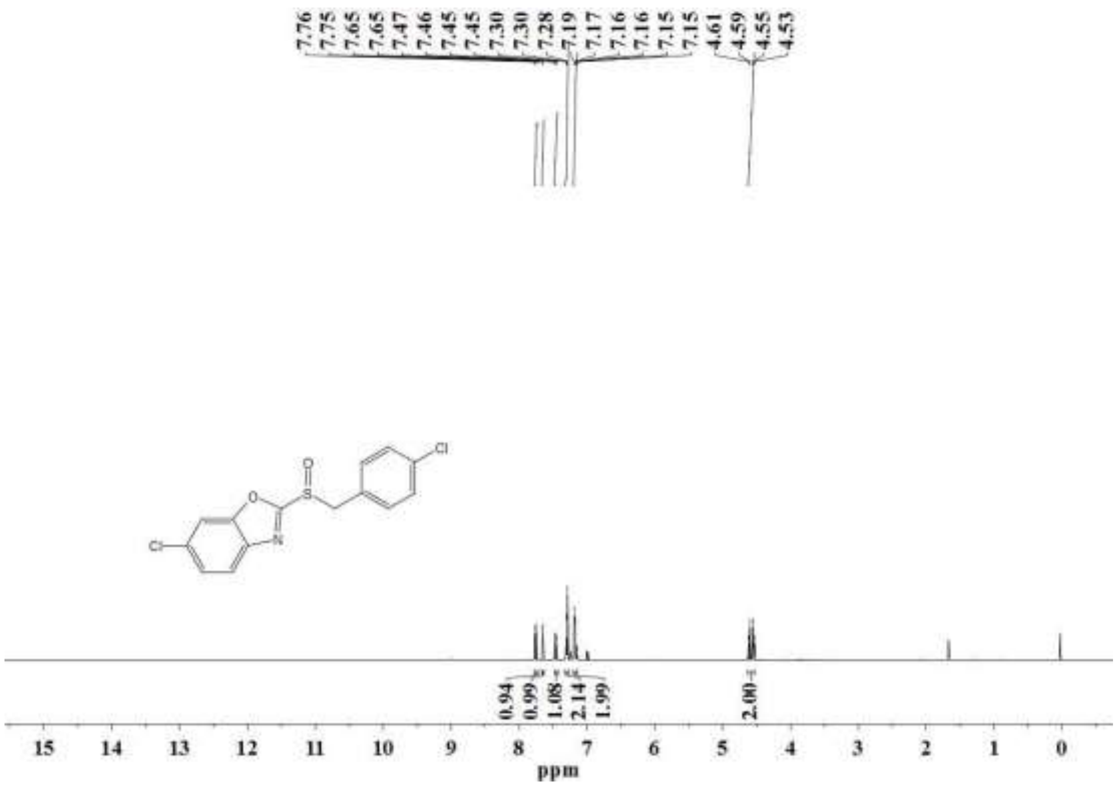

Figure S 38-1. ^{1}H NMR spectrum of compound **7b**.

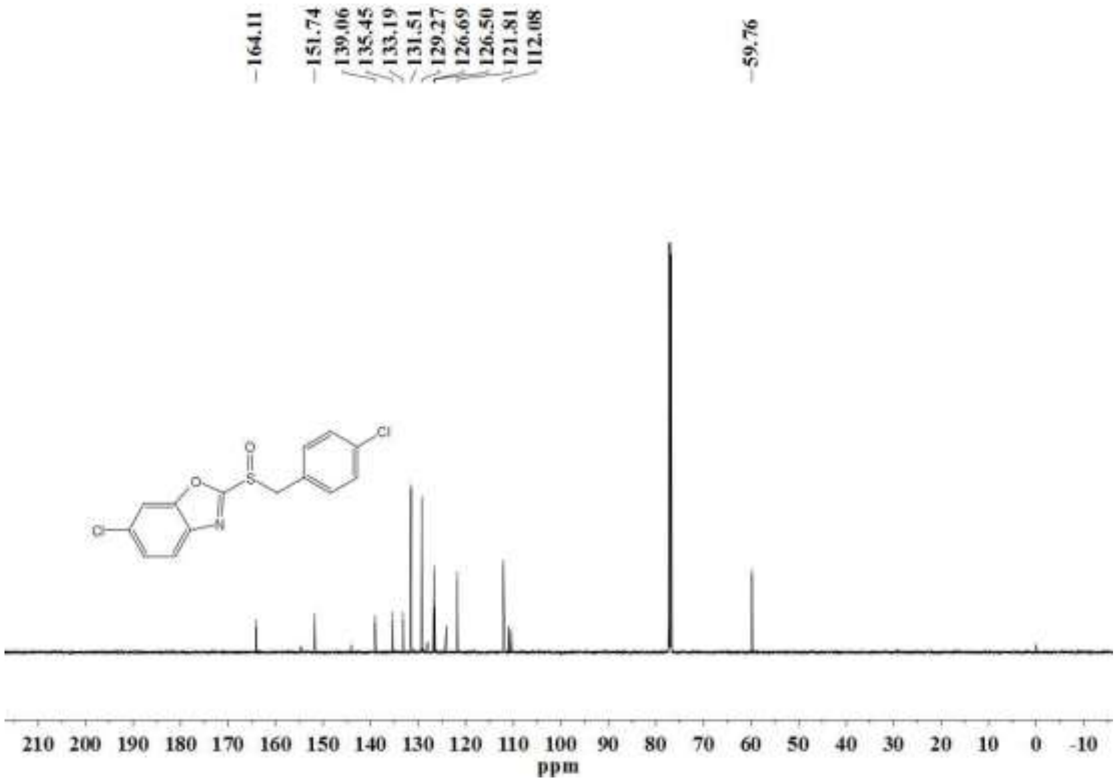

Figure S 38-2. ^{13}C NMR spectrum of compound **7b**.

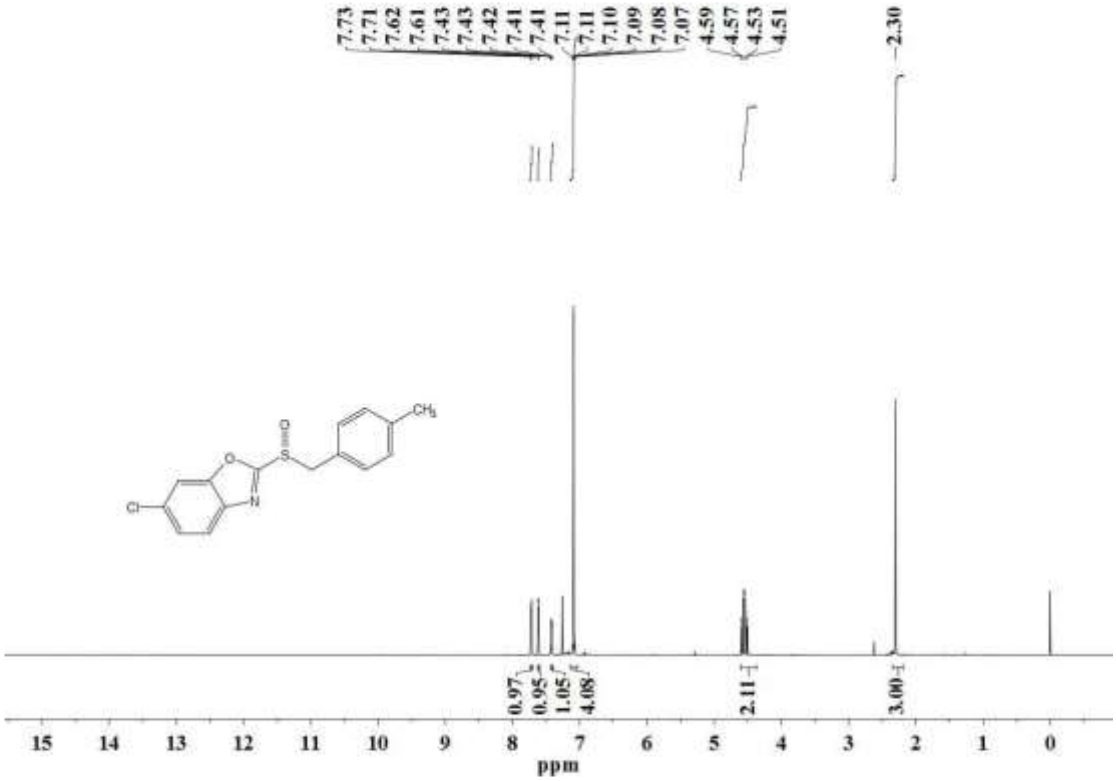

Figure S 39-1. ^{1}H NMR spectrum of compound **7c**.

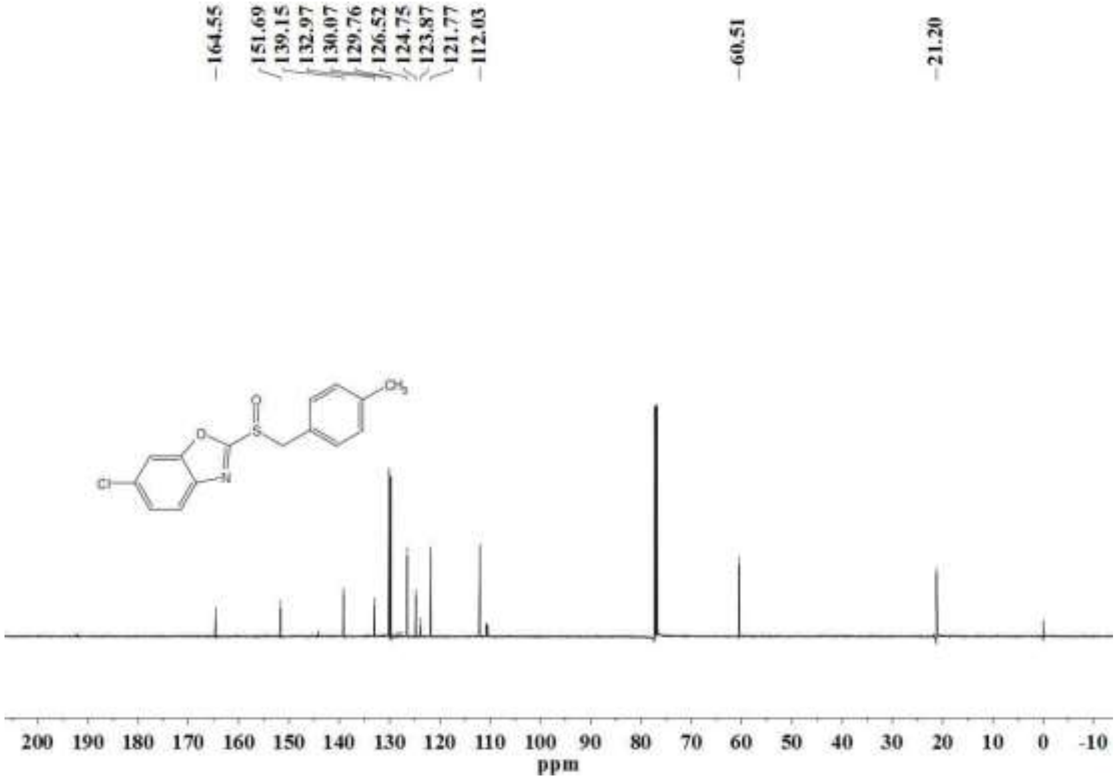

Figure S 39-2. ^{13}C NMR spectrum of compound **7c**.

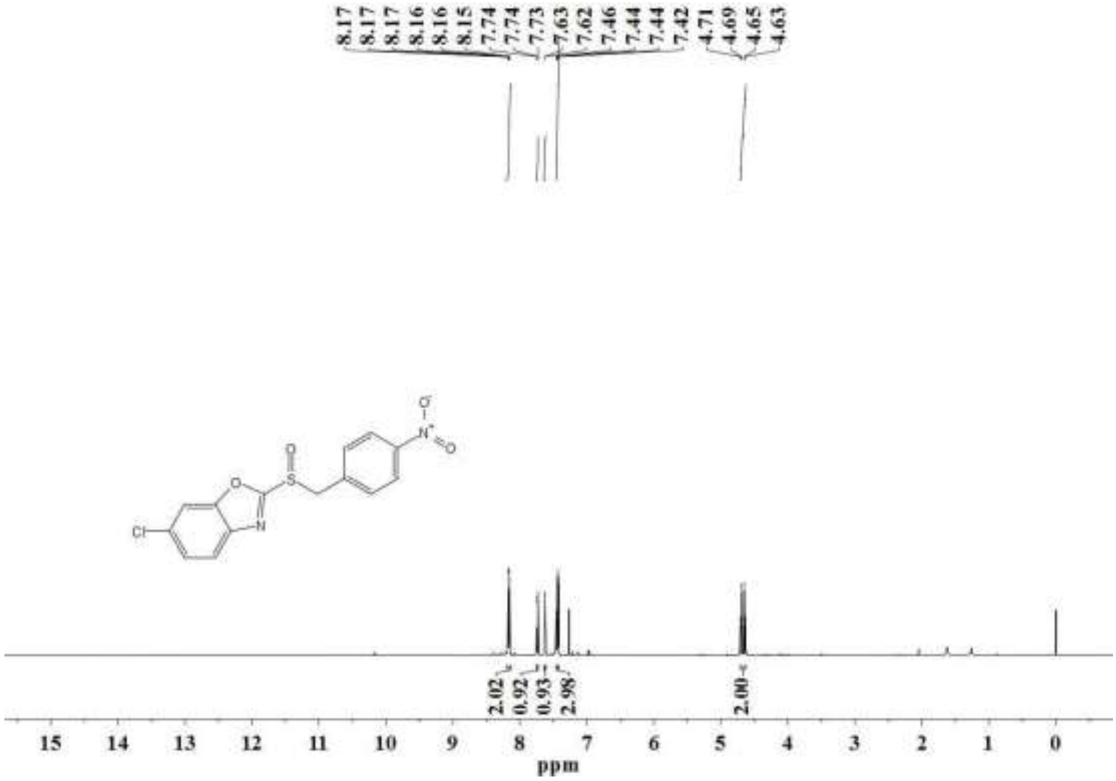

Figure S 40-1. ^{1}H NMR spectrum of compound **7d**.

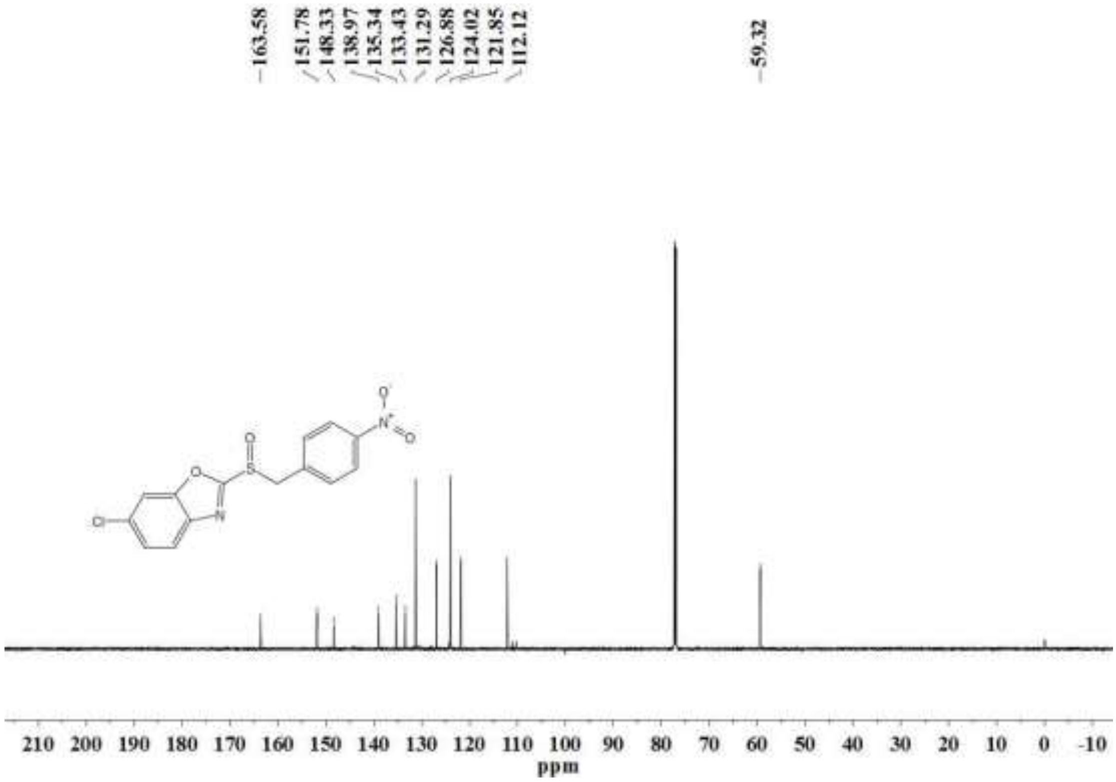

Figure S 40-2. ^{13}C NMR spectrum of compound **7d**.

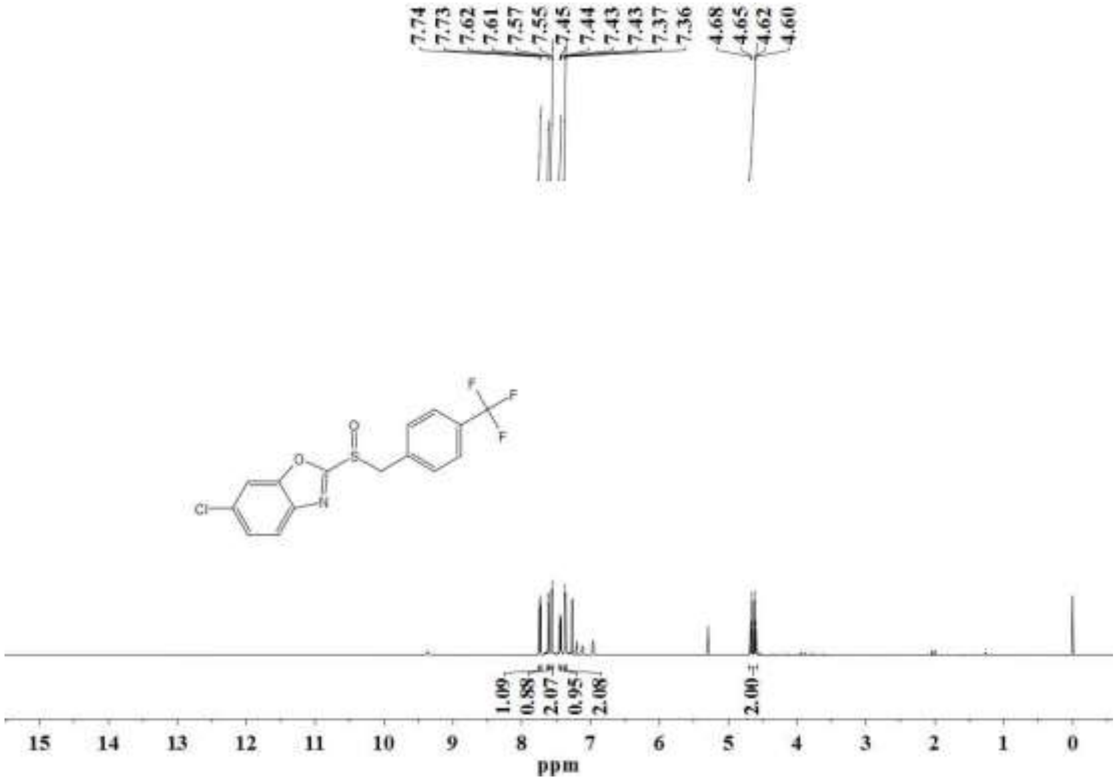

Figure S 41-1. ¹H NMR spectrum of compound **7e**.

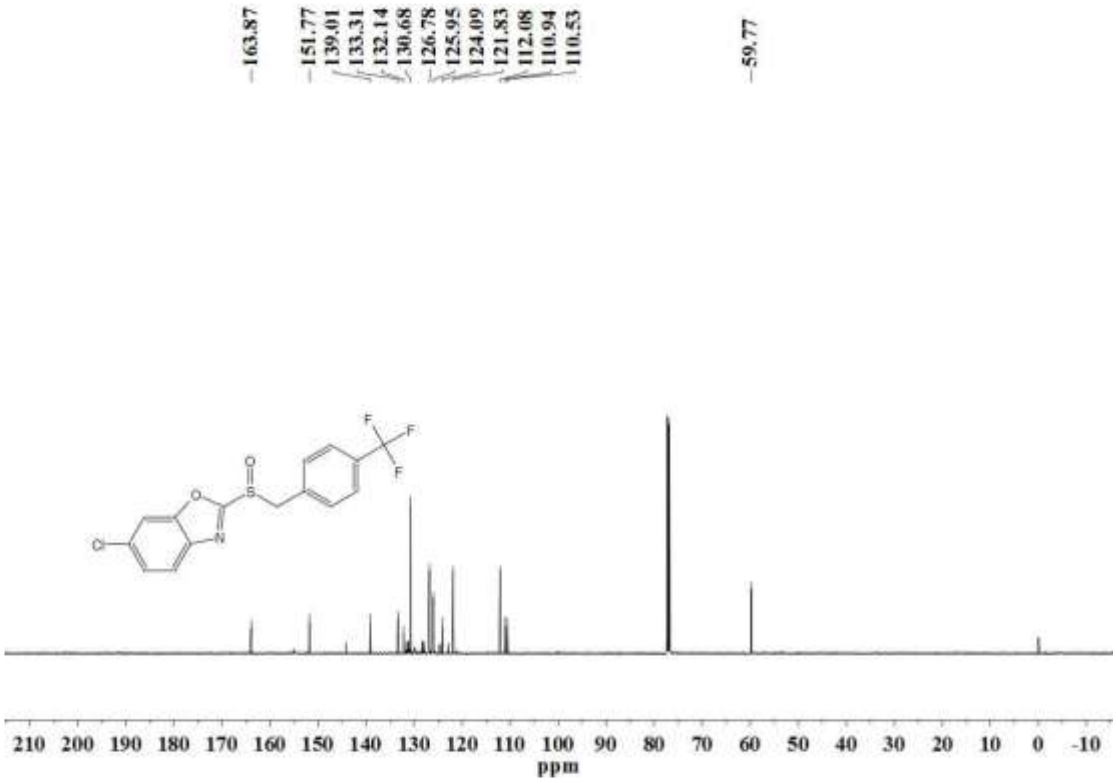

Figure S 41-2. ¹³C NMR spectrum of compound **7e**.

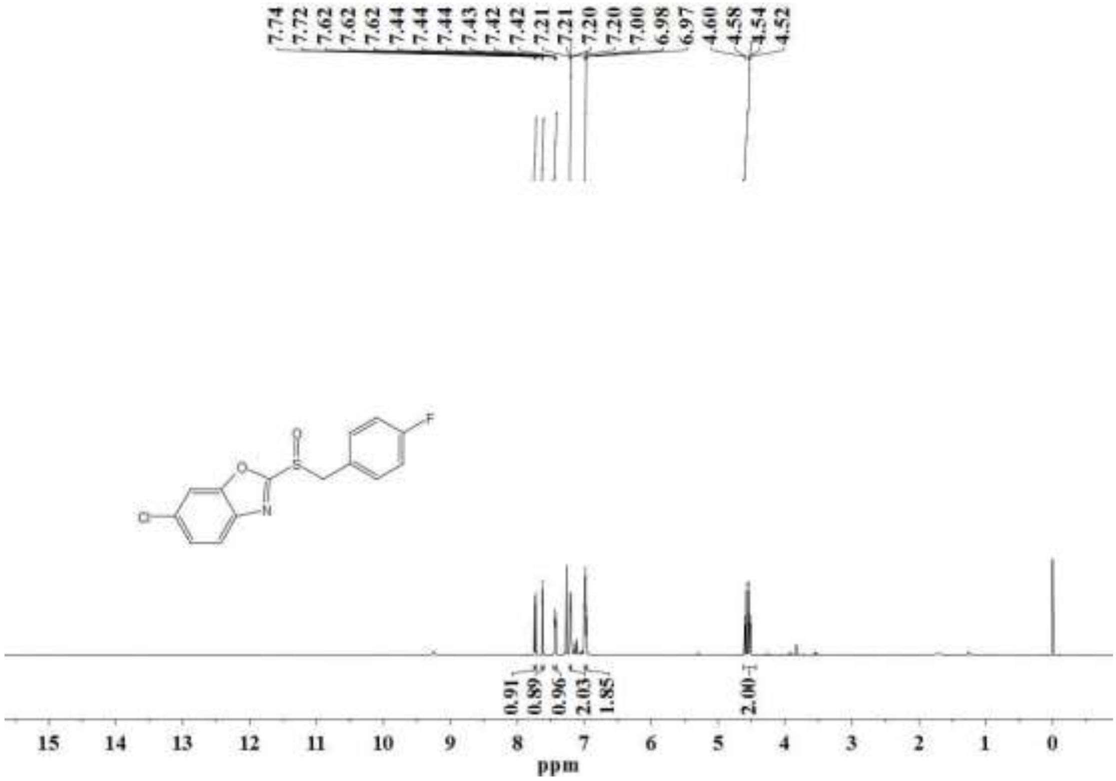

Figure S 42-1. ^{1}H NMR spectrum of compound **7f**.

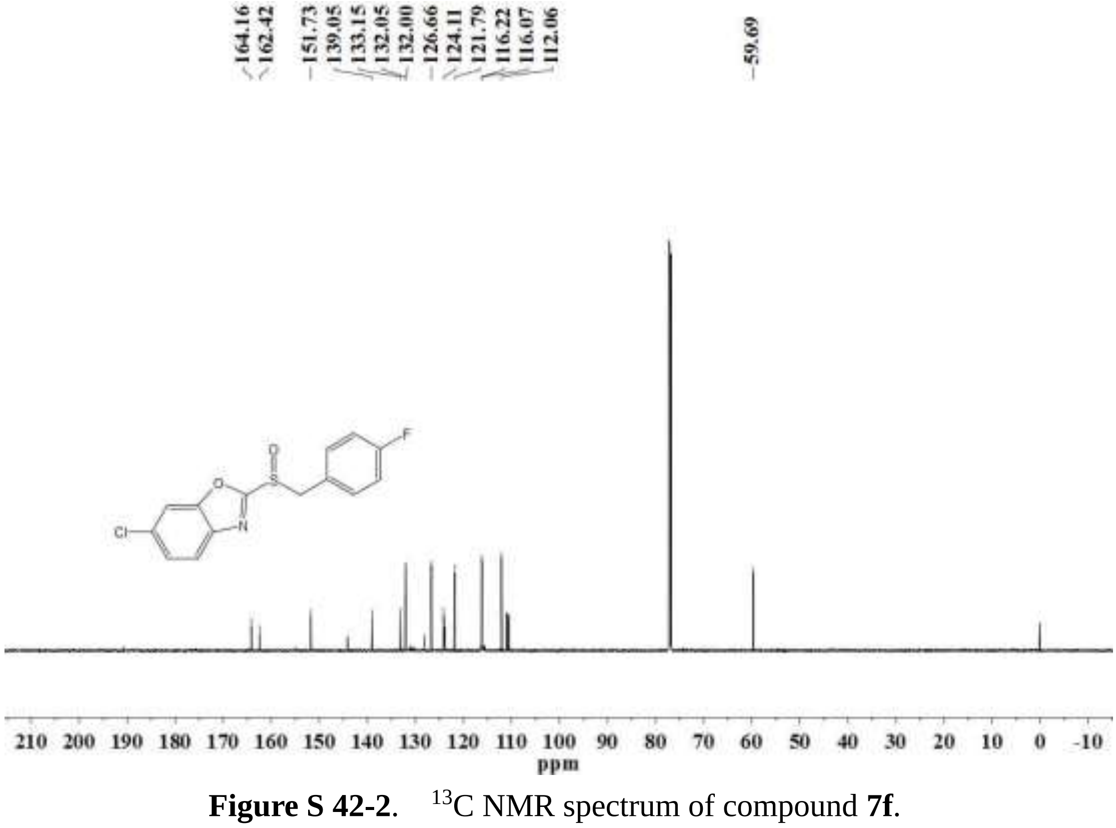

Figure S 42-2. ^{13}C NMR spectrum of compound **7f**.

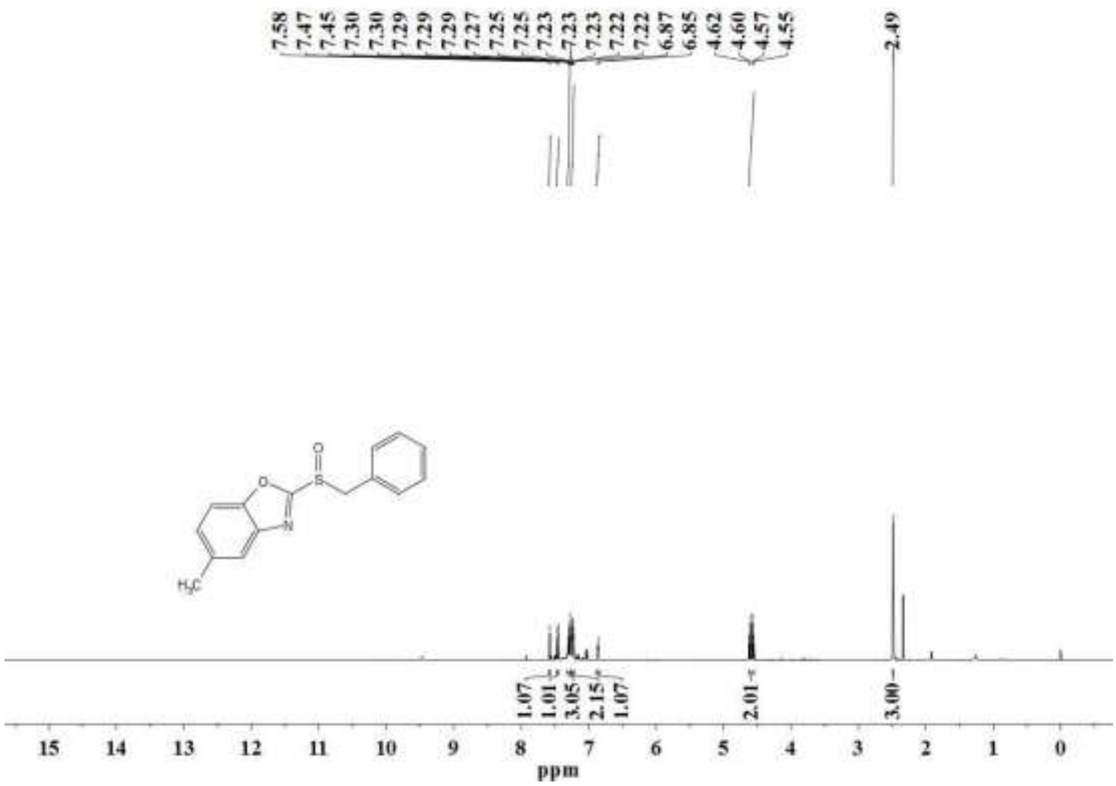

Figure S 43-1. ^{1}H NMR spectrum of compound **8a**.

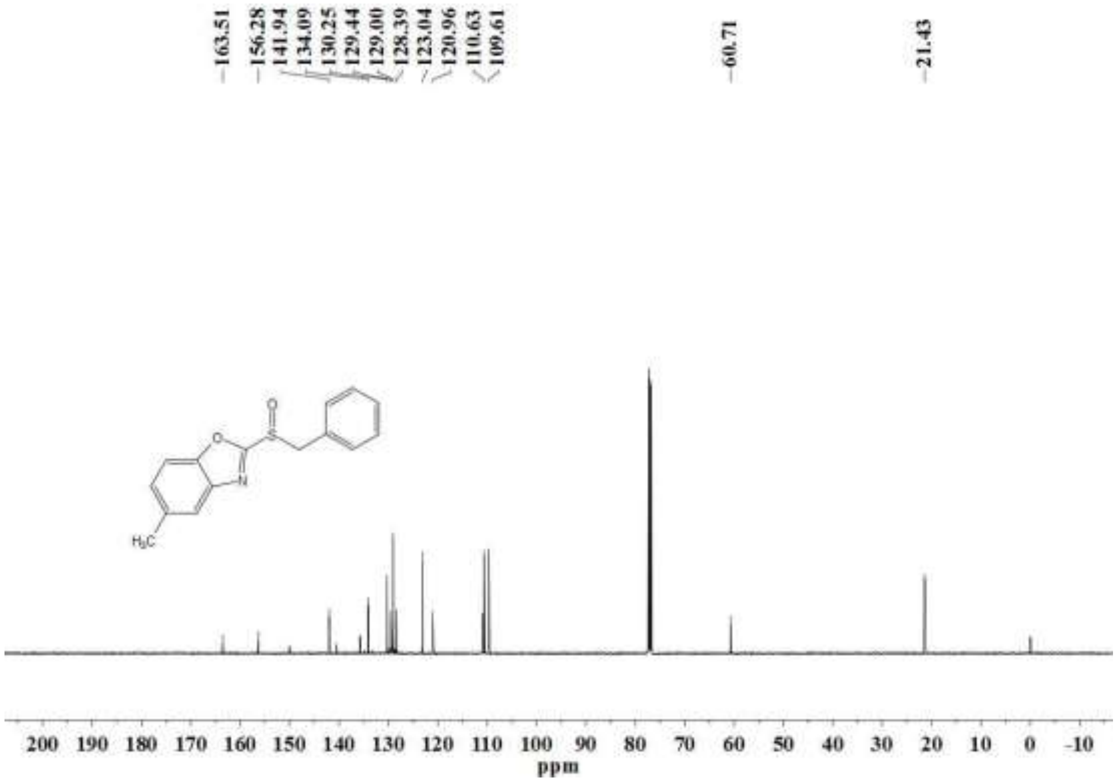

Figure S 43-2. ^{13}C NMR spectrum of compound **8a**.

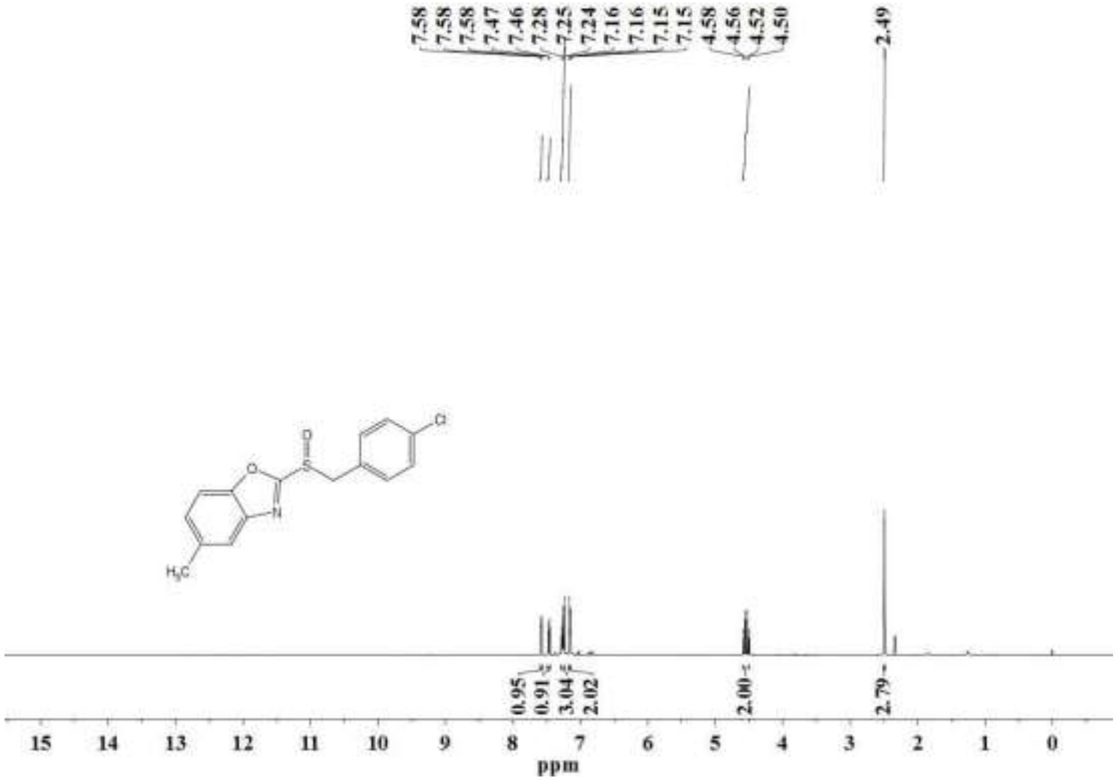

Figure S 44-1. ^{1}H NMR spectrum of compound **8b**.

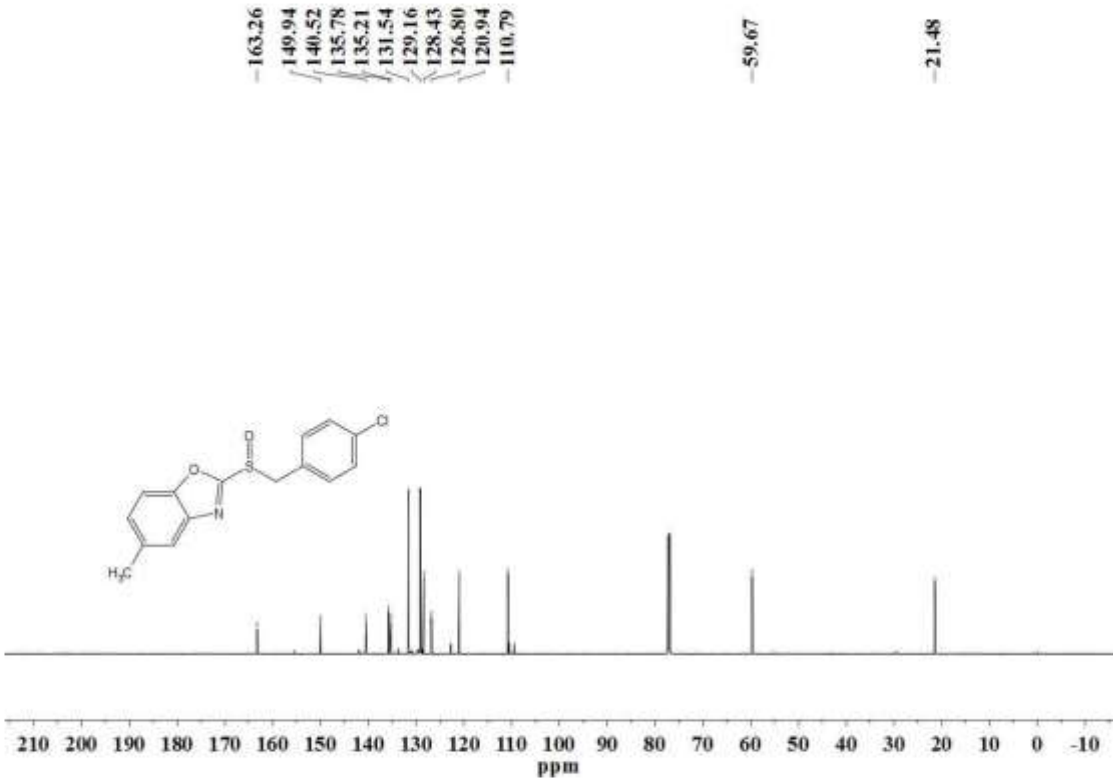

Figure S 44-2. ^{13}C NMR spectrum of compound **8b**.

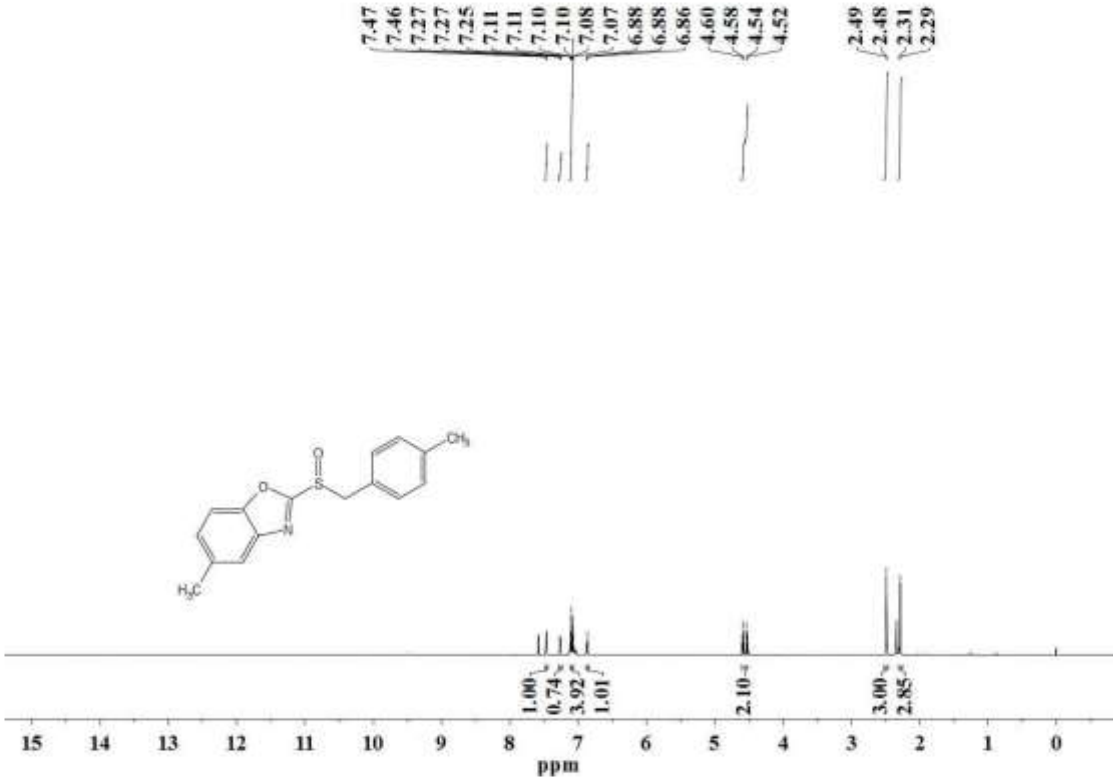

Figure S 45-1. ^{1}H NMR spectrum of compound **8c**.

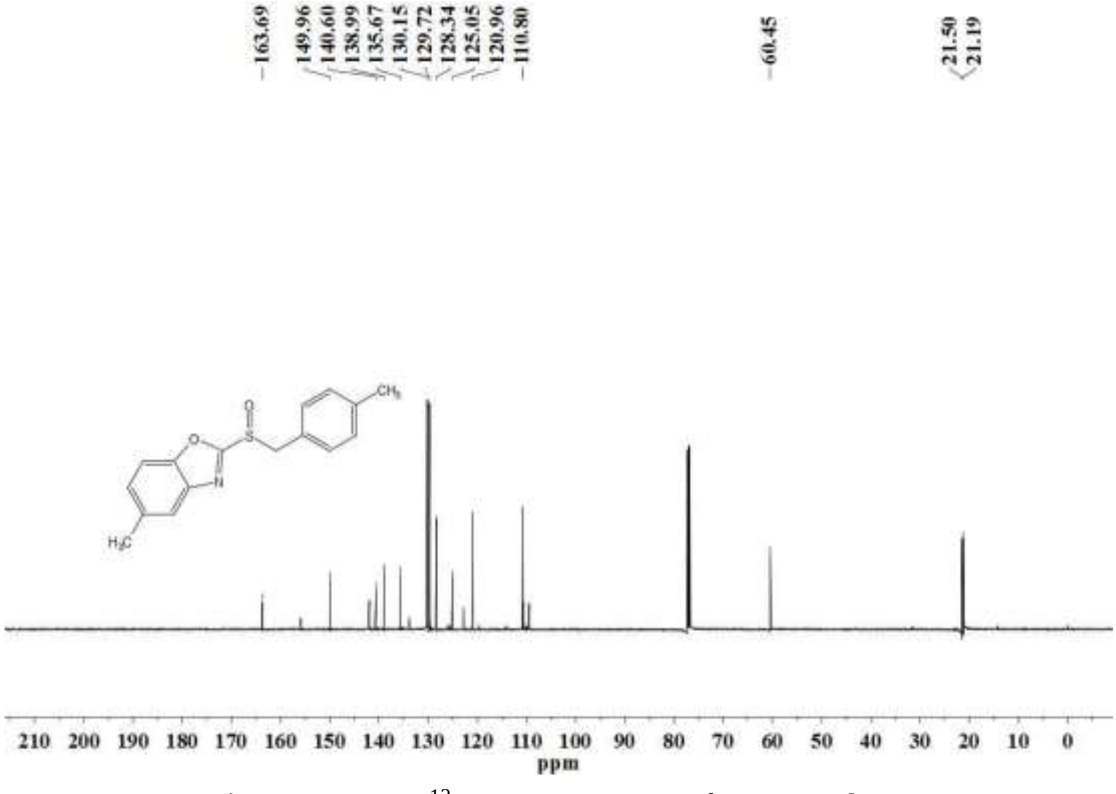

Figure S 45-2. ^{13}C NMR spectrum of compound **8c**.

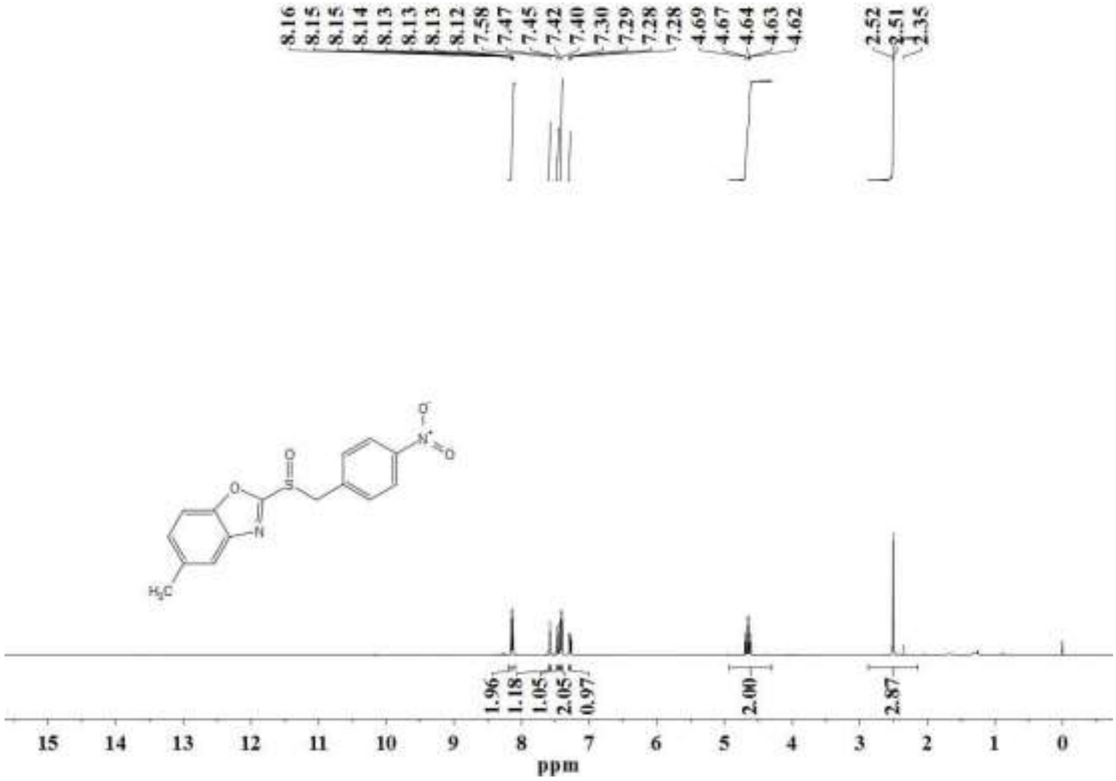

Figure S 46-1. ^{1}H NMR spectrum of compound **8d**.

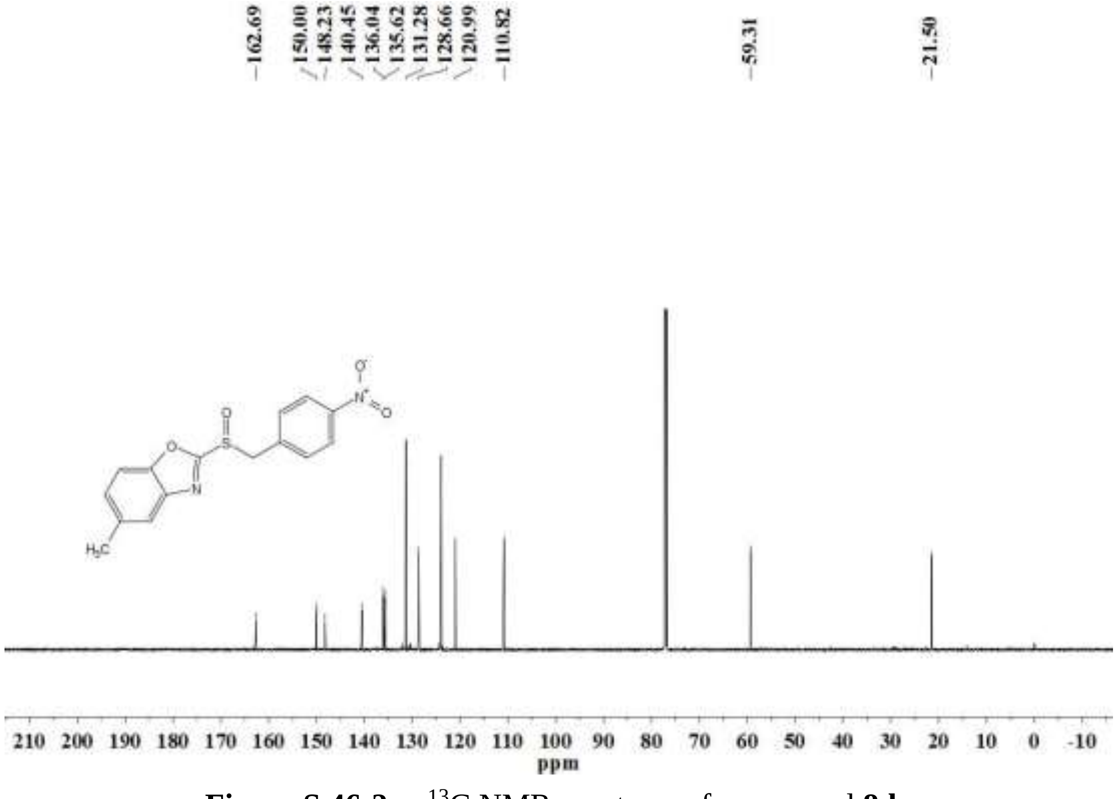

Figure S 46-2. ^{13}C NMR spectrum of compound **8d**.

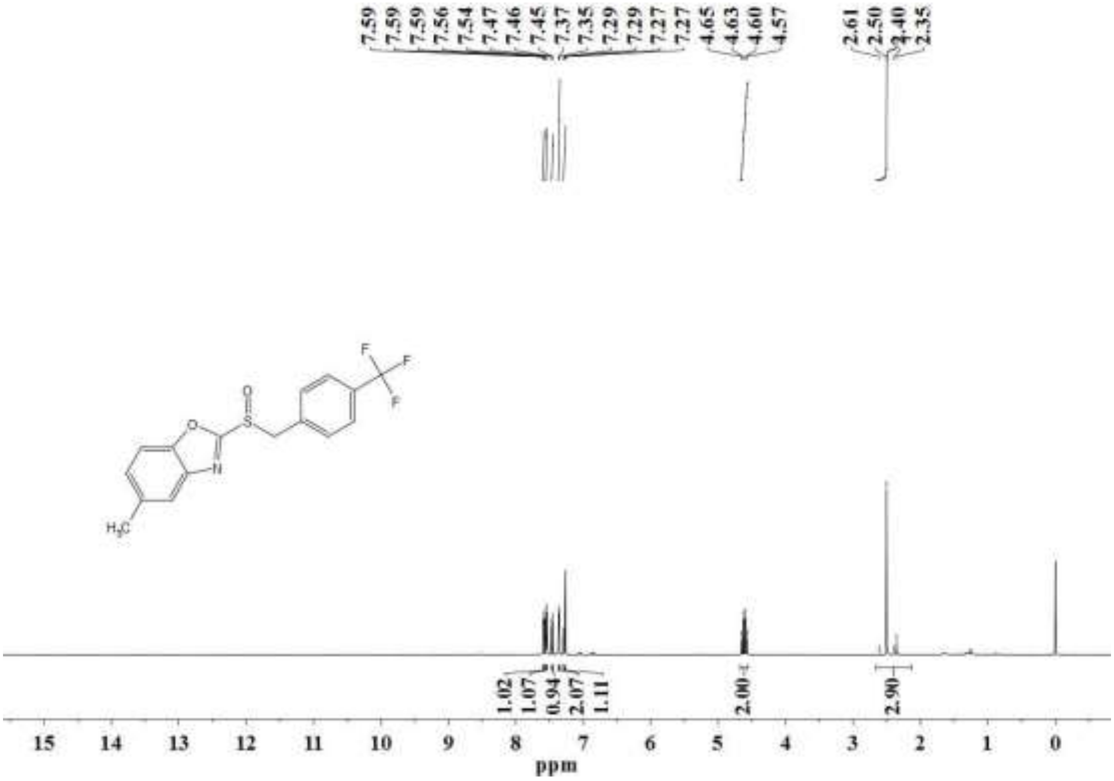

Figure S 47-1. ^{1}H NMR spectrum of compound **8e**.

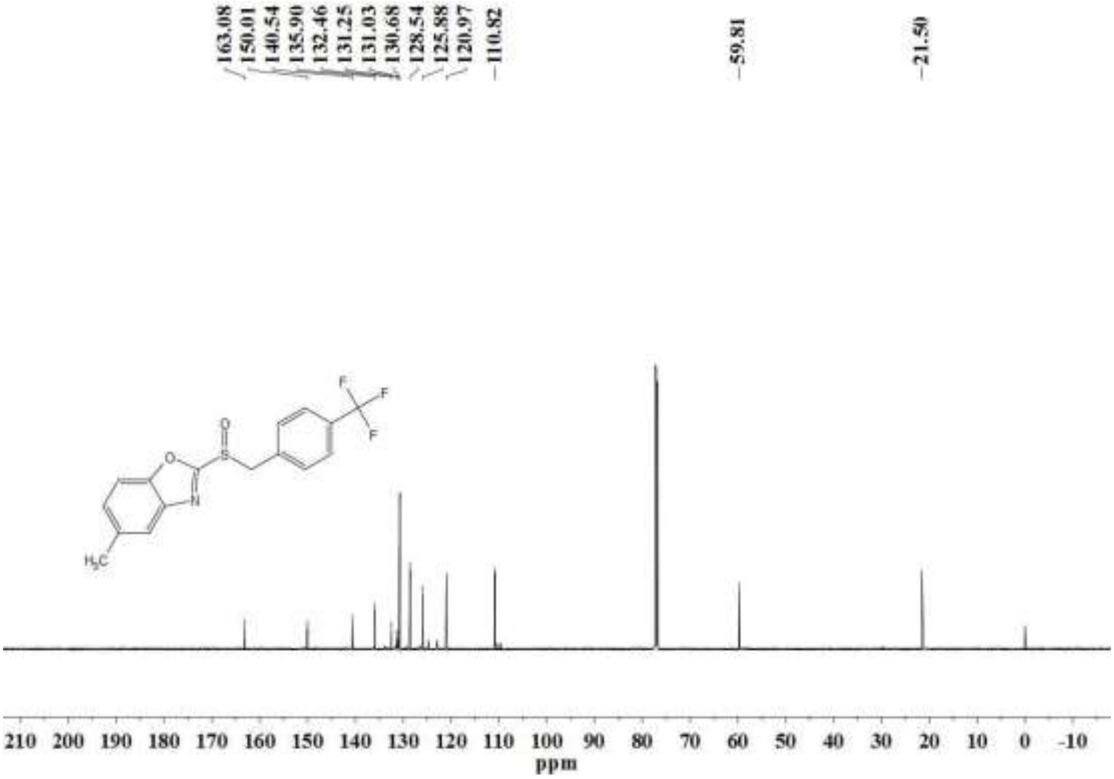

Figure S 47-2. ^{13}C NMR spectrum of compound **8e**.

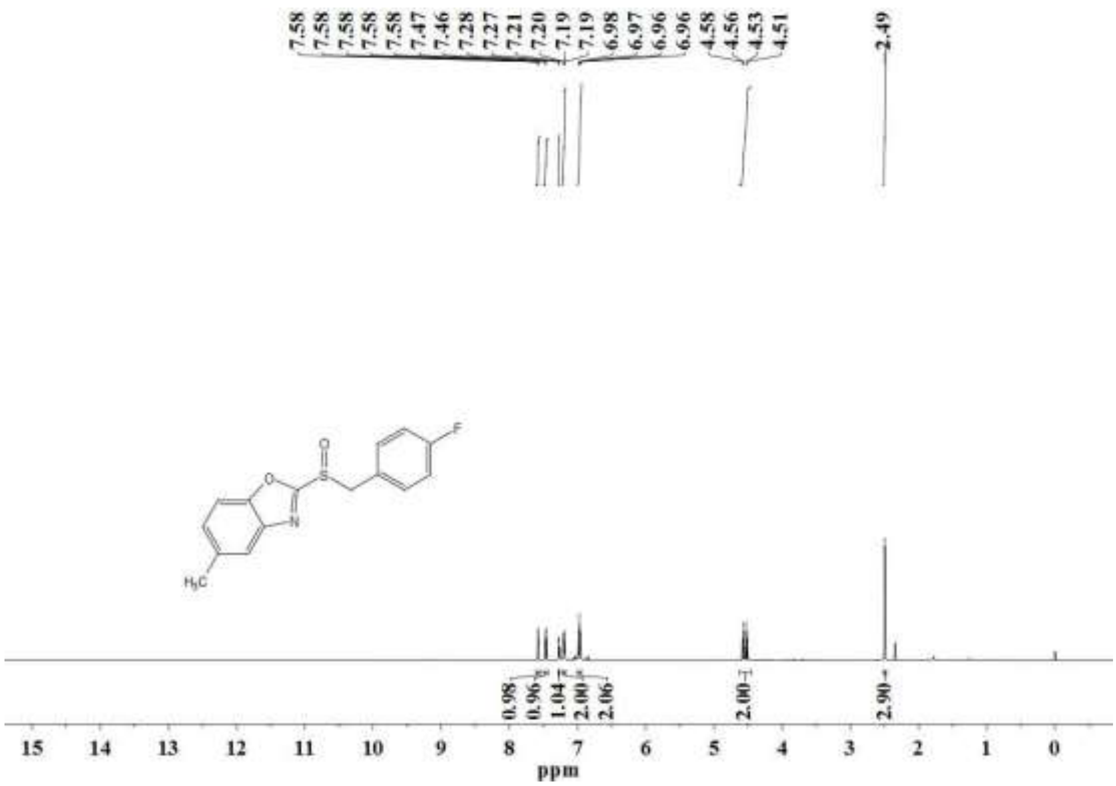

Figure S 48-1. ^{1}H NMR spectrum of compound **8f**.

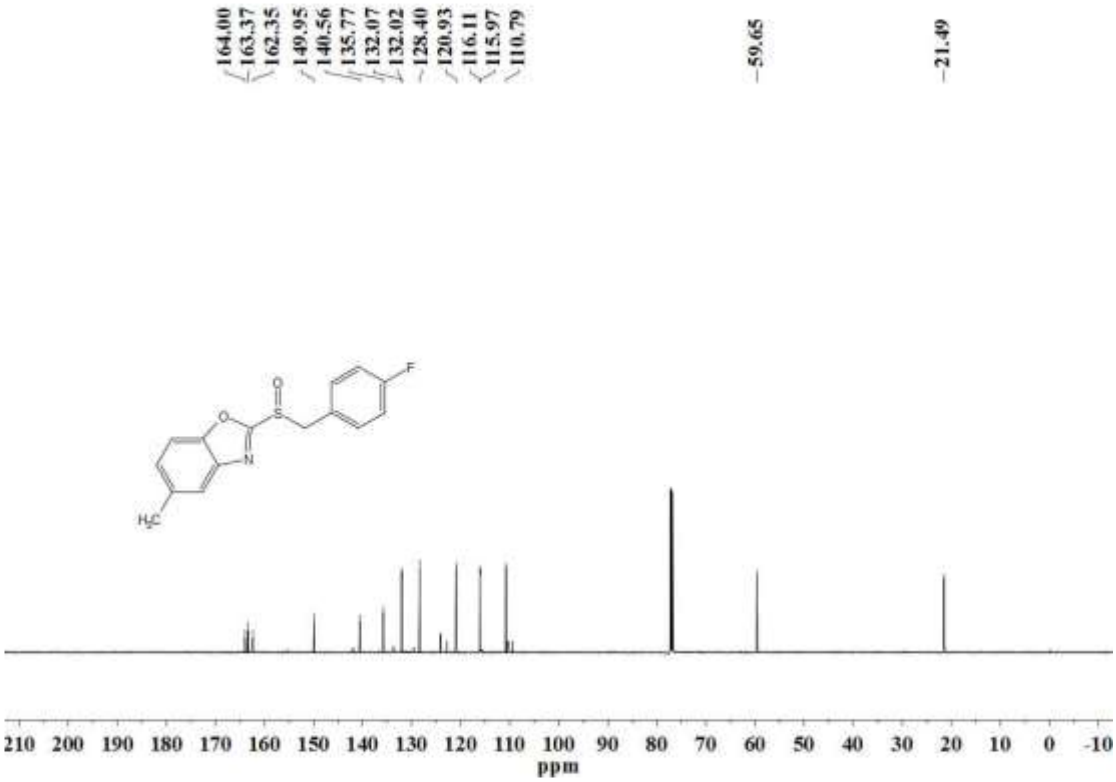

Figure S 48-2. ^{13}C NMR spectrum of compound **8f**.

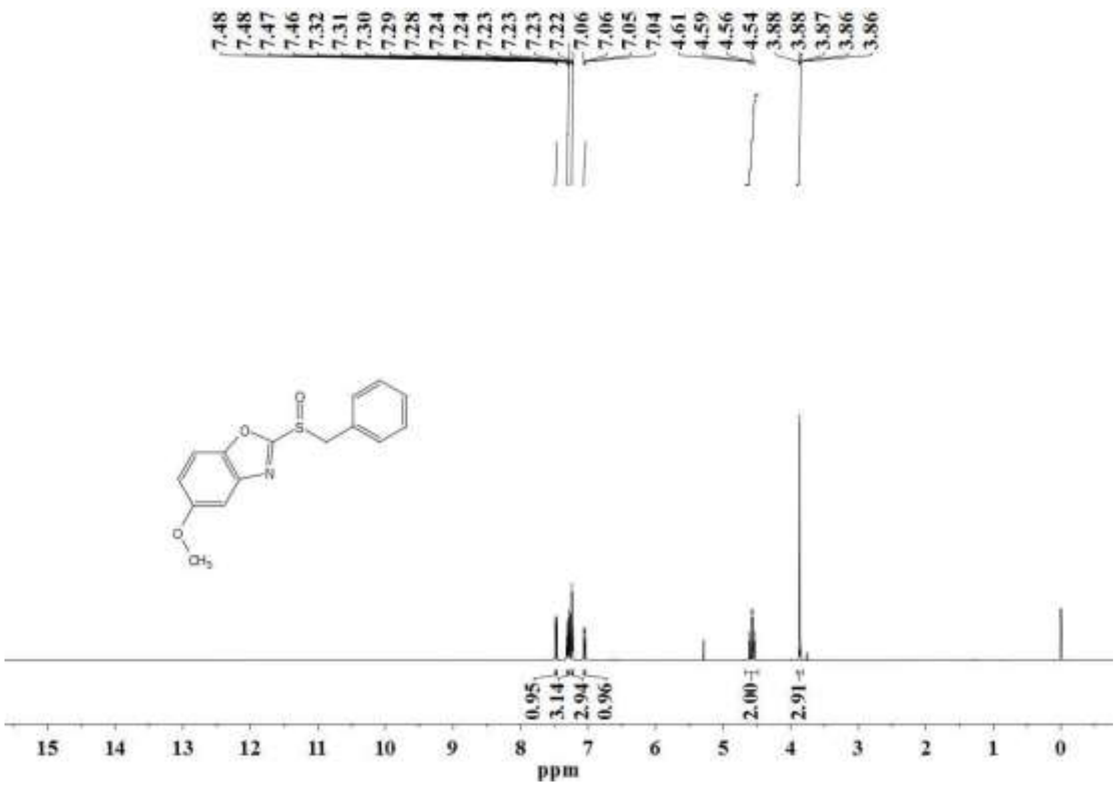

Figure S 49-1. ^{1}H NMR spectrum of compound **9a**.

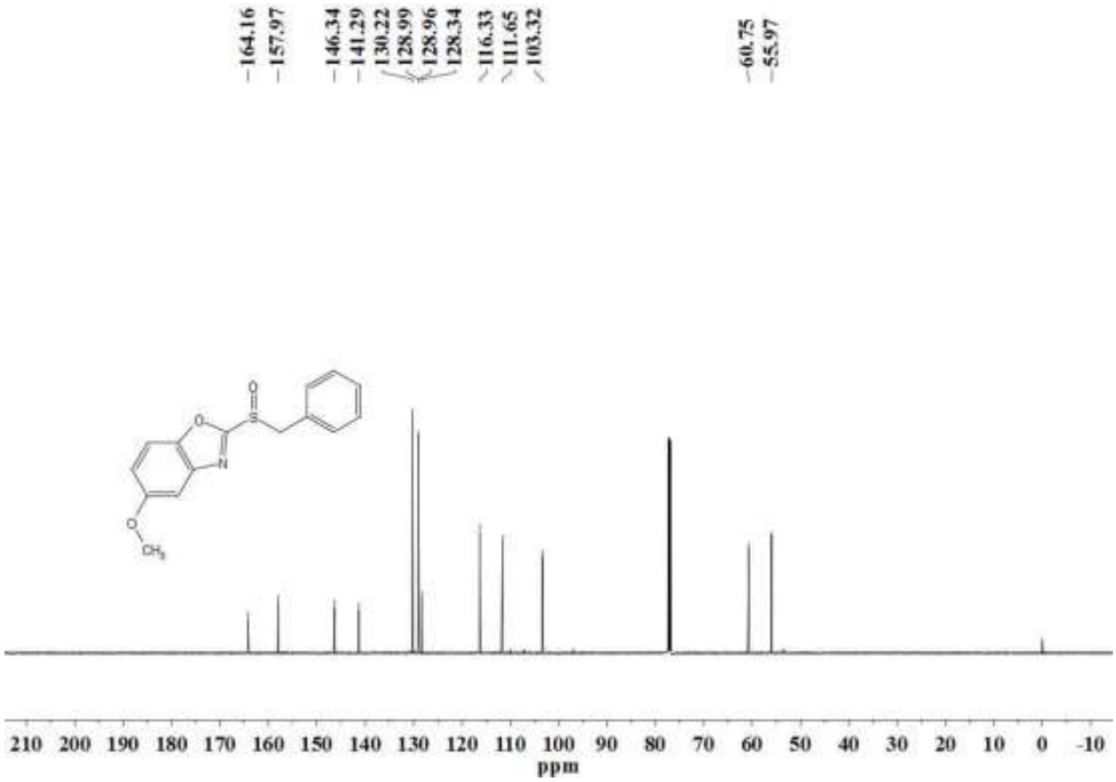

Figure S 49-2. ^{13}C NMR spectrum of compound **9a**.

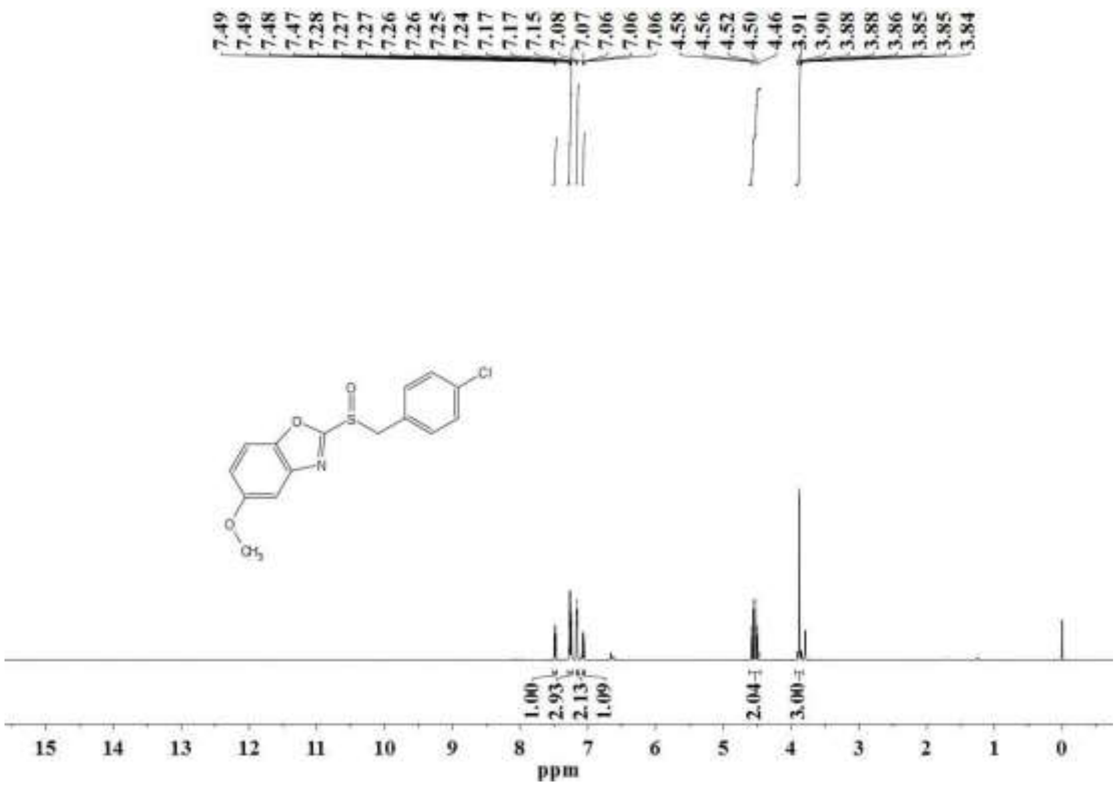

Figure S 50-1. ^{1}H NMR spectrum of compound **9b**.

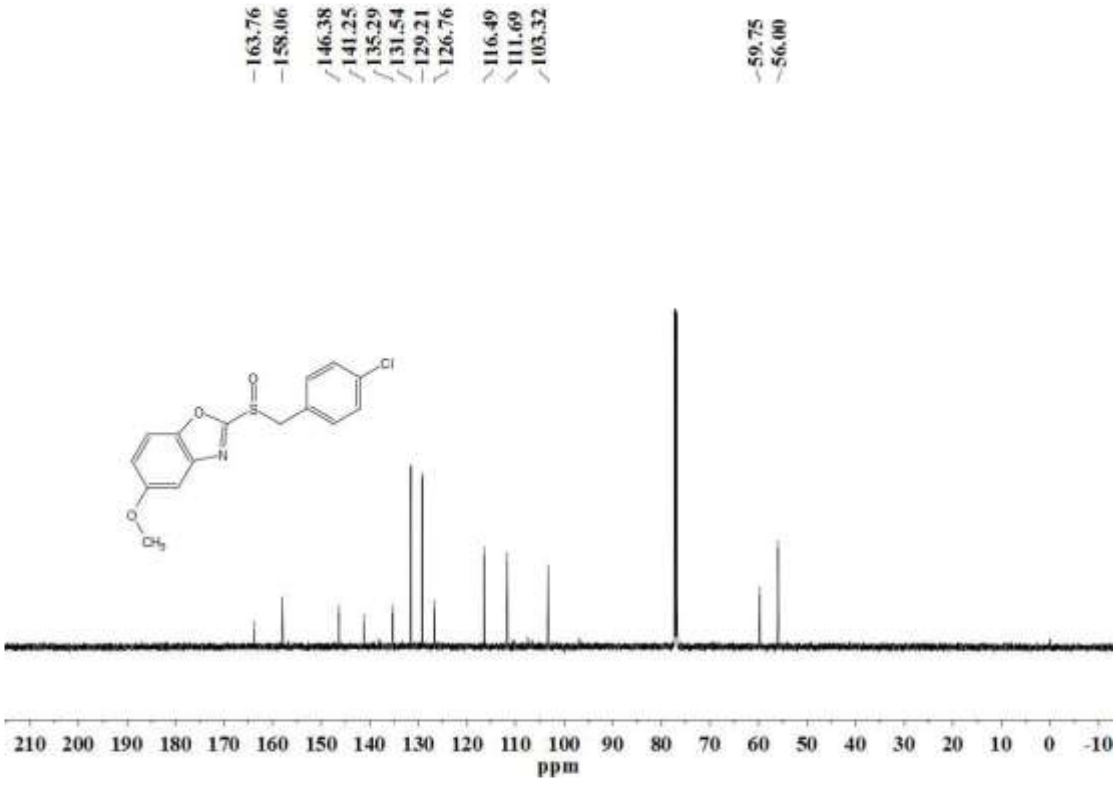

Figure S 50-2. ^{13}C NMR spectrum of compound **9b**.

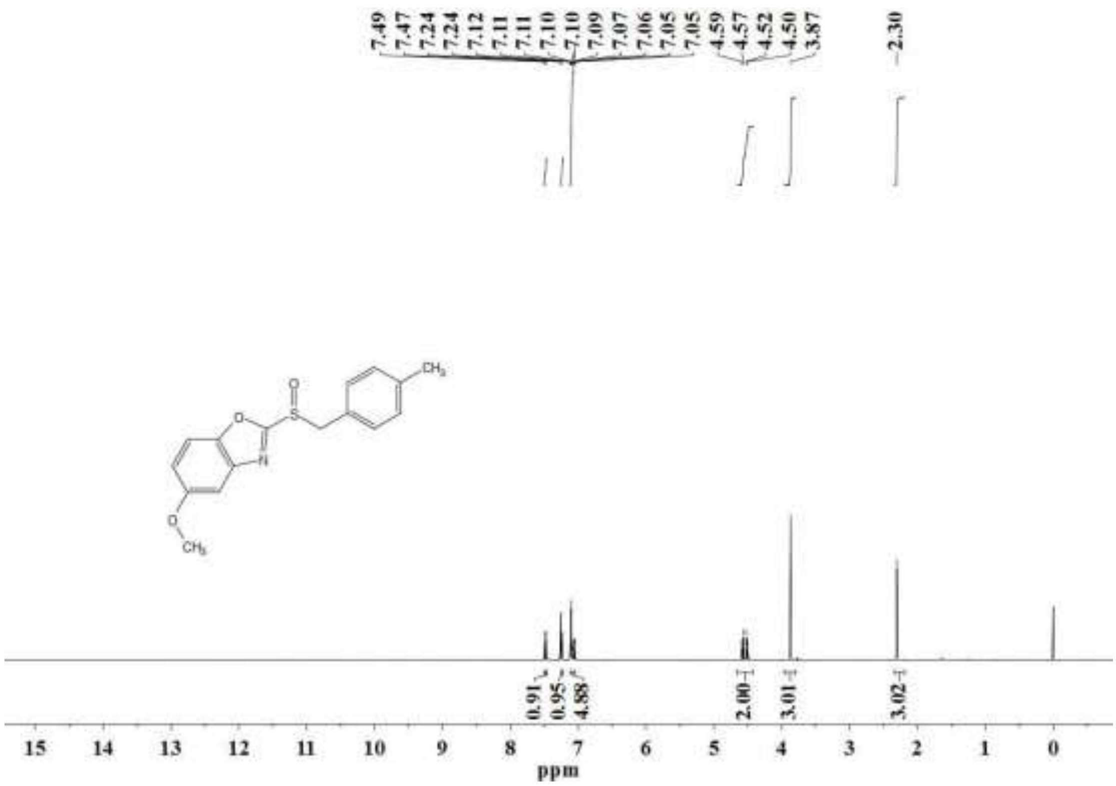

Figure S 51-1. ^{1}H NMR spectrum of compound **9c**.

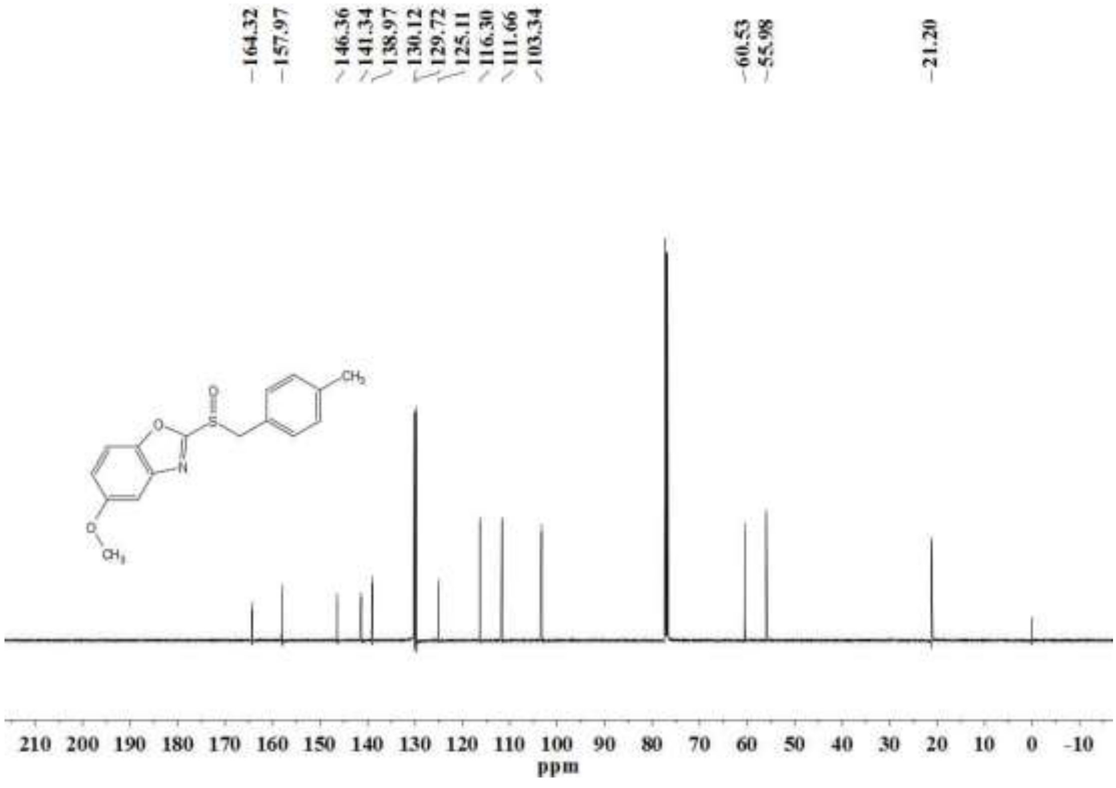

Figure S 51-2. ^{13}C NMR spectrum of compound **9c**.

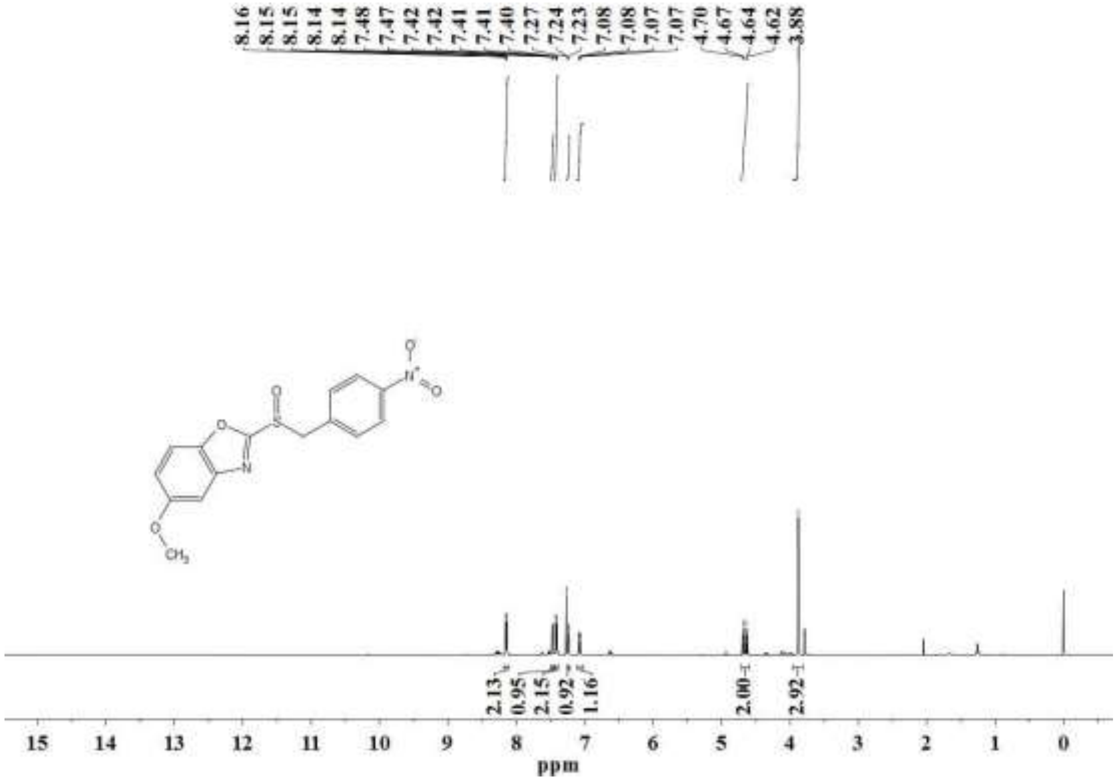

Figure S 52-1. ^{1}H NMR spectrum of compound **9d**.

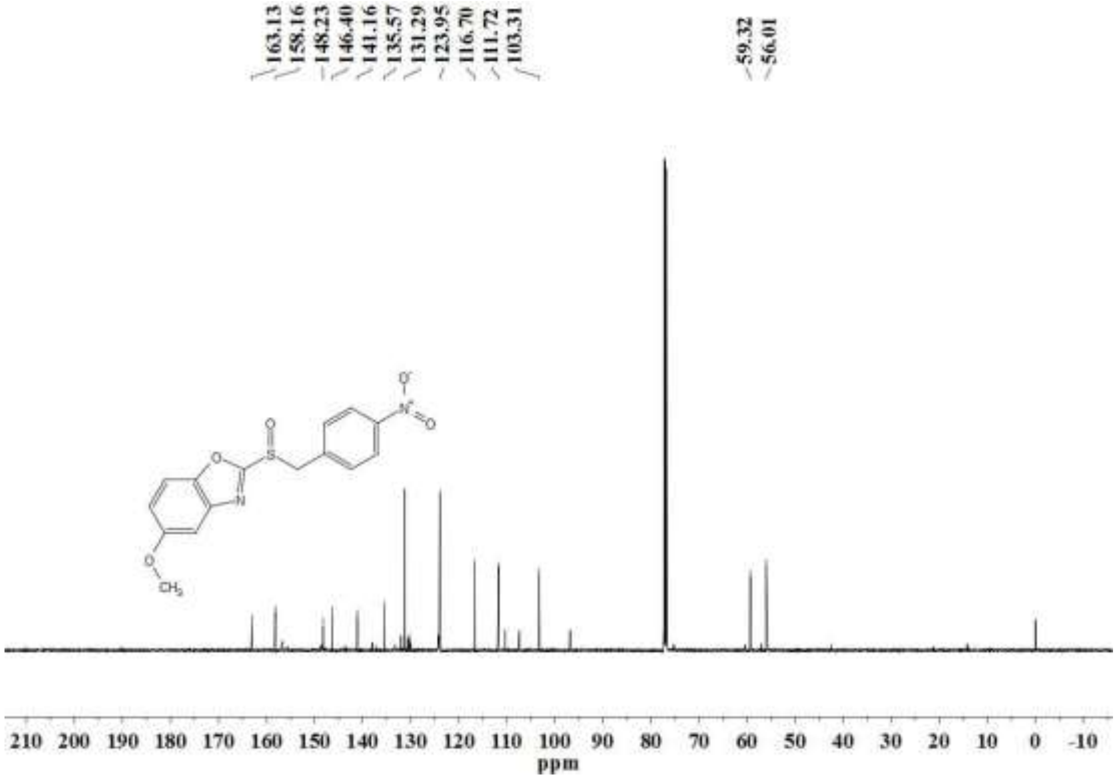

Figure S 52-2. ^{13}C NMR spectrum of compound **9d**.

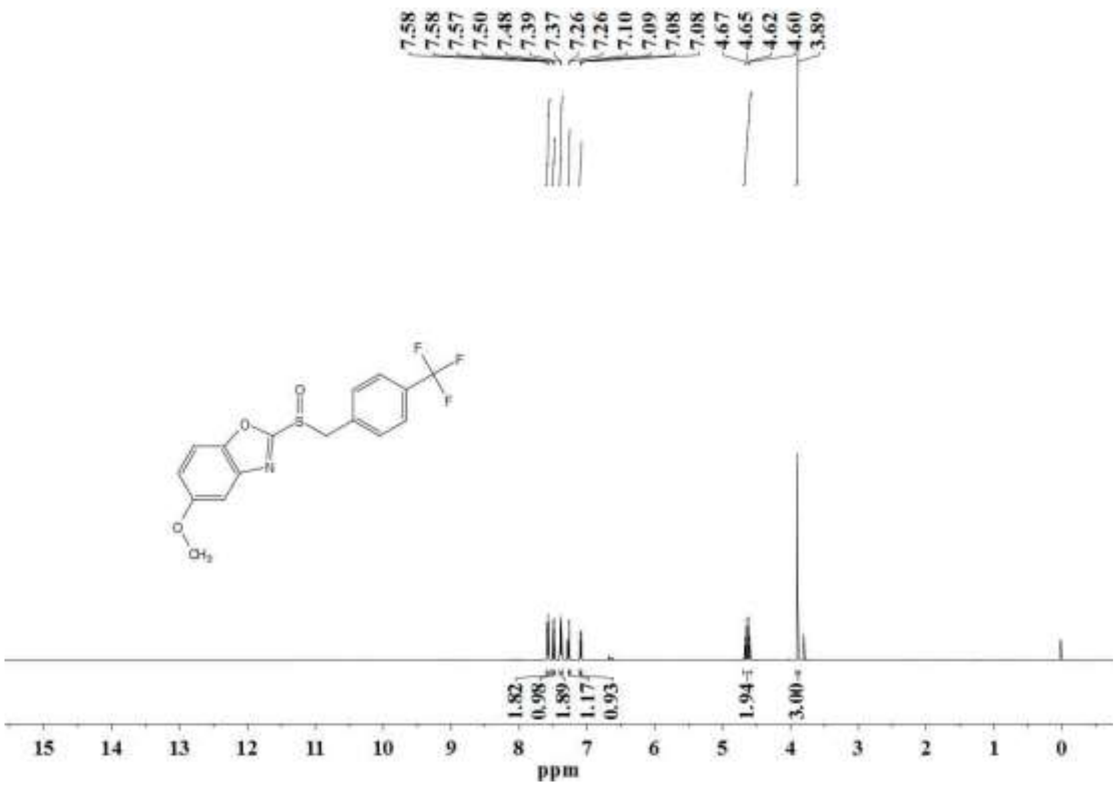

Figure S 53-1. ^{1}H NMR spectrum of compound **9e**.

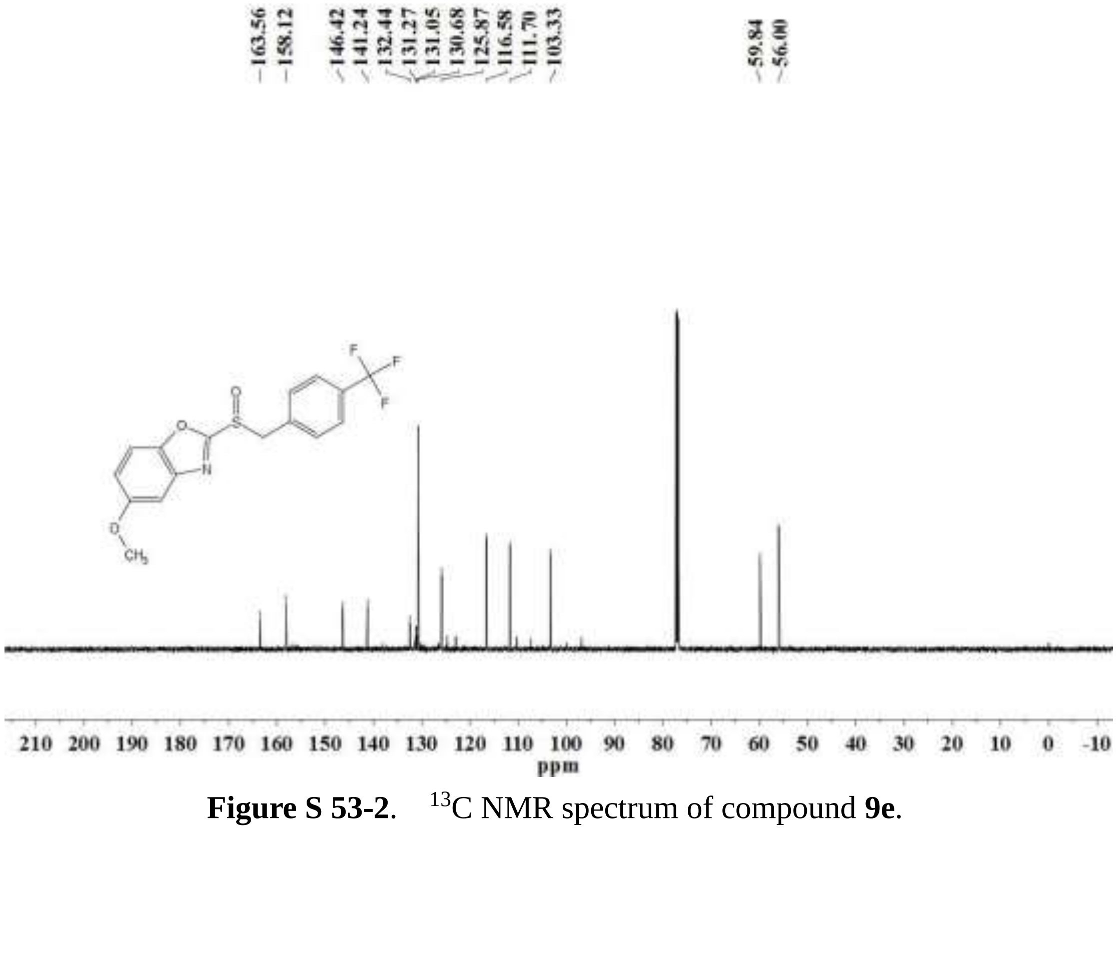

Figure S 53-2. ^{13}C NMR spectrum of compound **9e**.

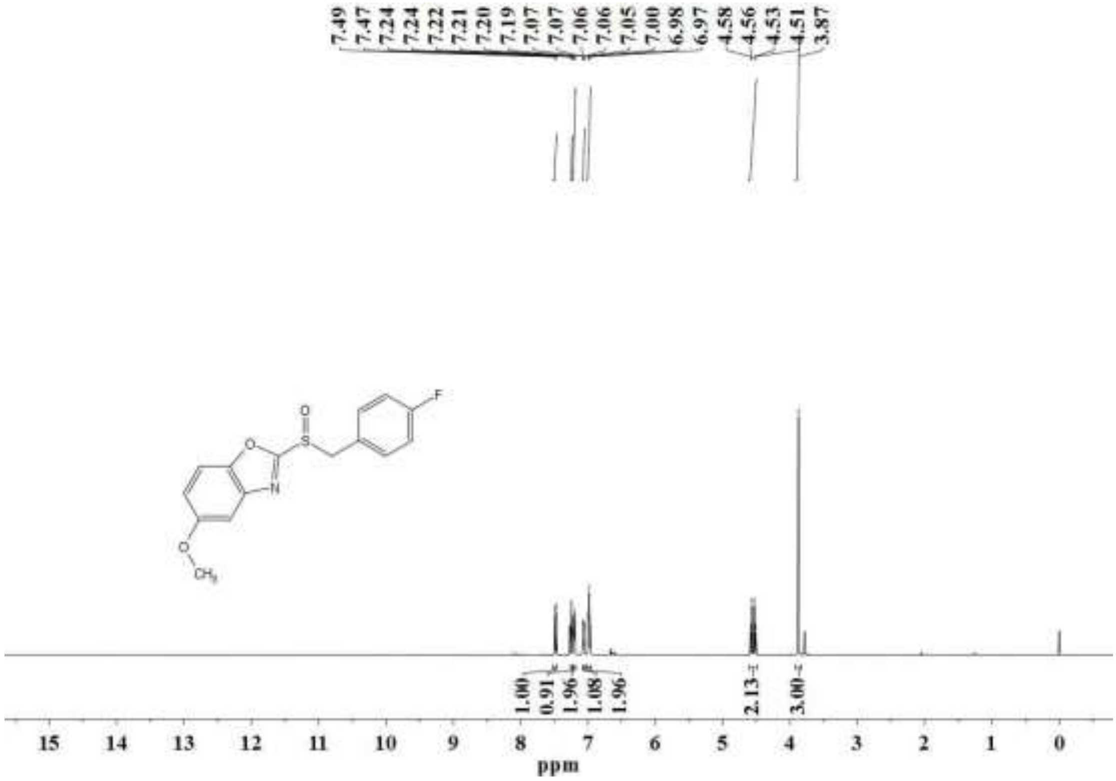

Figure S 54-1. ^{1}H NMR spectrum of compound **9f**.

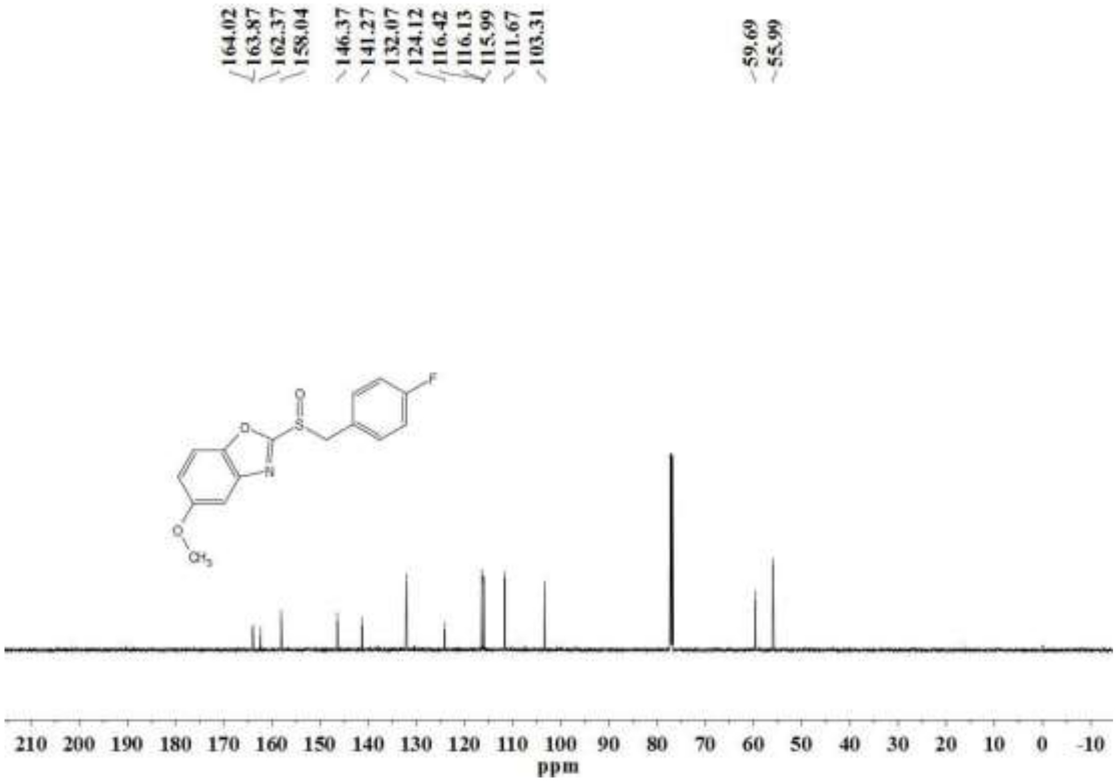

Figure S 54-2. ^{13}C NMR spectrum of compound **9f**.
